# High-quality assembly of the reference genome for scarlet sage, *Salvia splendens*, an economically important ornamental plant

**DOI:** 10.1093/gigascience/giy068

**Published:** 2018-06-19

**Authors:** Ai-Xiang Dong, Hai-Bo Xin, Zi-Jing Li, Hui Liu, Yan-Qiang Sun, Shuai Nie, Zheng-Nan Zhao, Rong-Feng Cui, Ren-Gang Zhang, Quan-Zheng Yun, Xin-Ning Wang, Fatemeh Maghuly, Ilga Porth, Ri-Chen Cong, Jian-Feng Mao

**Affiliations:** 1 Beijing Key Laboratory of Greening Plants Breeding, Beijing Institute of Landscape Architecture, Beijing, 100102, China; 2 Beijing Advanced Innovation Center for Tree Breeding by Molecular Design, National Engineering Laboratory for Tree Breeding, Key Laboratory of Genetics and Breeding in Forest Trees and Ornamental Plants, Ministry of Education, College of Biological Sciences and Technology, Beijing Forestry University, Beijing, 100083, China; 3 Beijing Ori-Gene Science and Technology Co. Ltd, Beijing, 102206, China; 4 Plant Biotechnology Unit, Department of Biotechnology, BOKU-VIBT, University of Natural Resources and Life Sciences, Muthgasse 18, 1190 Vienna, Austria; 5 Département des sciences du bois et de la forêt, Pavillon Charles-Eugène-Marchand, 1030, Avenue de la Médecine, Université Laval, Québec (Québec) G1V 0A6, Canada

**Keywords:** annotation, evolution, reference genome, *Salvia splendens*, scarlet sage; single-molecule real-time sequencing

## Abstract

**Background:**

*Salvia splendens* Ker-Gawler, scarlet or tropical sage, is a tender herbaceous perennial widely introduced and seen in public gardens all over the world. With few molecular resources, breeding is still restricted to traditional phenotypic selection, and the genetic mechanisms underlying phenotypic variation remain unknown. Hence, a high-quality reference genome will be very valuable for marker-assisted breeding, genome editing, and molecular genetics.

**Findings:**

We generated 66 Gb and 37 Gb of raw DNA sequences, respectively, from whole-genome sequencing of a largely homozygous scarlet sage inbred line using Pacific Biosciences (PacBio) single-molecule real-time and Illumina HiSeq sequencing platforms. The PacBio *de novo* assembly yielded a final genome with a scaffold N50 size of 3.12 Mb and a total length of 808 Mb. The repetitive sequences identified accounted for 57.52% of the genome sequence, and  54,008 protein-coding genes were predicted collectively with *ab initio* and homology-based gene prediction from the masked genome. The divergence time between *S. splendens* and *Salvia miltiorrhiza* was estimated at 28.21 million years ago (Mya). Moreover, 3,797 species-specific genes and 1,187 expanded gene families were identified for the scarlet sage genome.

**Conclusions:**

We provide the first genome sequence and gene annotation for the scarlet sage. The availability of these resources will be of great importance for further breeding strategies, genome editing, and comparative genomics among related species.

## Data Description

### Background information

**Figure 1:**
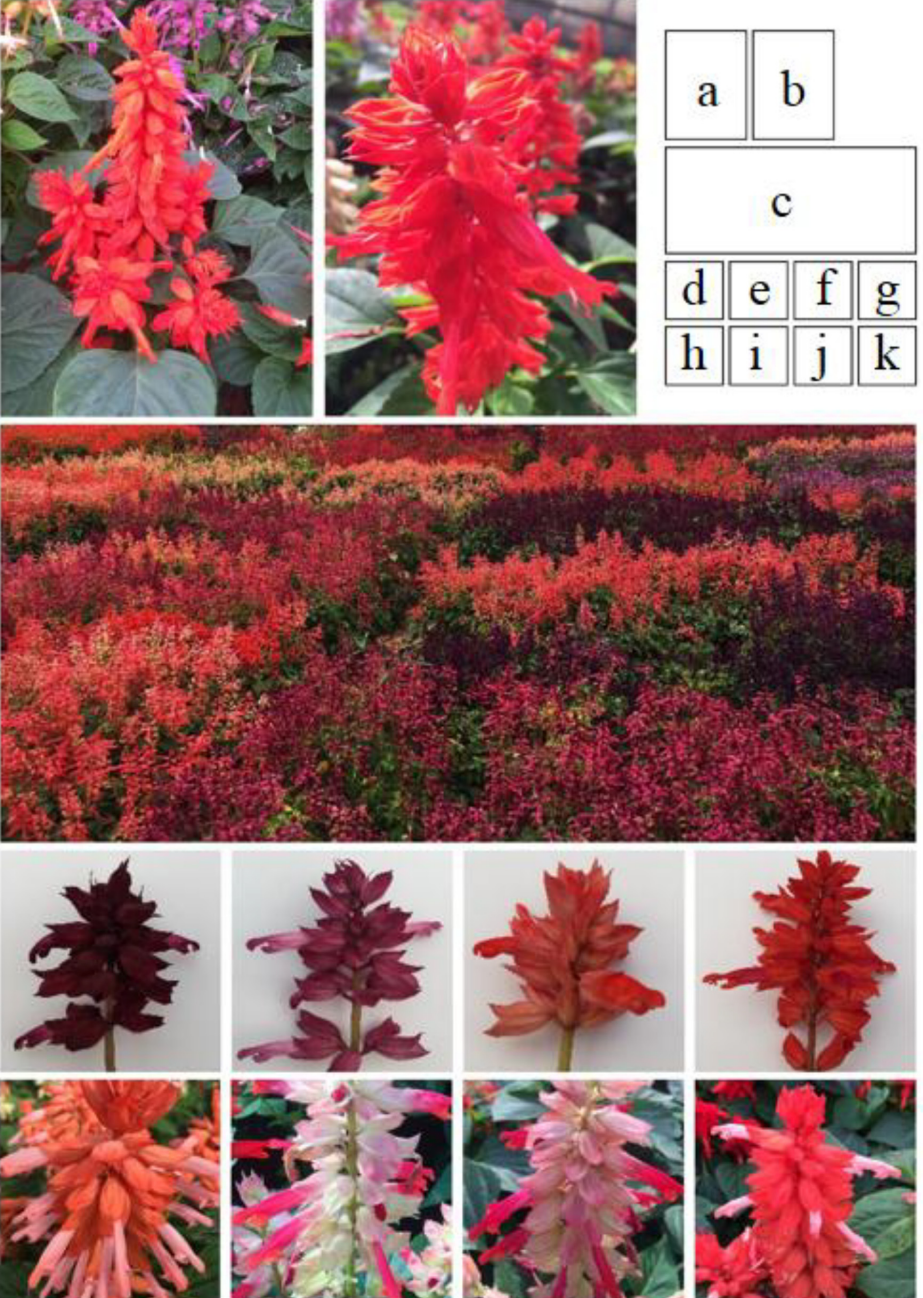
Images of the scarlet sage, *Salvia splendens*. **(a-b)** Flowers of the sequenced cultivar of *S. splendens*, “Aoyunshenghuo (Olympic flame).” **(c)** The scarlet sage with different flower colors in bedding. **(d-k)** The scarlet sage with flowers of different pure colors, or bi-colors.

*Salvia* L., with nearly 1,000 species of shrubs, herbaceous perennials, and annuals, is the largest genus in the mint family (Lamiaceae: Nepetoideae: Mentheae: Salviinae) [1–4]. The genus is widely distributed throughout the world. Many species of this genus are extensively used for culinary purposes, essential oil production, and Chinese herbal remedies, such as *S. officinalis* [3] and *S. miltiorrhiza* (Danshen). Additionally, they are used as ornamental plants valued for their flowers and for their aromatic foliage, such as *S. splendens* (Fig. 1a-k).

*Salvia splendens* (National Center for Biotechnology Information [NCBI] taxon ID:180675), scarlet or tropical sage, is a herbaceous perennial species that is native to Brazil. While it is a perennial in warmer climate zones, it grows as an annual in cooler areas. *Salvia splendens*,characterized by its dense flowers, wide variation of colors (e.g., scarlet, purple, pink, blue, lavender, salmon, yellow green, white, and bicolor), and long-lasting flowering (3–9 weeks or longer), is a very popular bedding plant that is widely cultivated in public gardens all over the world [3, 5]. Additionally, *S. splendens* can provide outstanding visual effects when grown in beds, borders, and containers; and its long life span ranges from late spring to the occurrence first frost. Furthermore, the flower is easy to maintain and fairly free of pests and diseases due to Lamiaceae's characteristic insect-repellent fragrance content [6]. The plant blends nicely with other annuals and perennial plants for the best visual effects in an ensemble setting. In addition, this plant requires little deadheading and it attracts various butterfly species. *Salvia splendens* is a prolific and durable bloomer, thrives in full sun, and survives in a large range of soil moisture regimes.

Traditional breeding activities using phenotypic selection as well as performing targeted variety hybridizations between elite cultivars have resulted in a large number of new cultivars with different performances regarding flowering characteristics (e.g., related to color, flowering time, and flowering period), individual growth performance, height, and tolerance to moisture and temperature extremes. However, little is known about the molecular mechanisms underlying such economically important characteristics for ornamental varieties. To date, few genetic markers [7] are available for marker-assistant breeding and genetic modification.

Here, we present the first high-quality genome assembly for *S. splendens* with a hybrid assembly strategy using Pacific Biosciences (PacBio) single-molecule real-time (SMRT) and Illumina HiSeq short-read sequencing platforms. The genome assembly, its structural and functional annotation, provide a valuable reference for the genomic dissection of the phenotypic variation in *Salvia* and new breeding strategies. This reference genome could also be used in comparative genomics with the recently released *Salvia* genome (*S. miltiorrhiza*) [8,9] and the mint genome (*Mentha longifolia*) [10] to study the biosynthesis of important fragrant and medicinal compounds.

### Plant material

We chose the elite variety *S. splendens*, “Aoyunshenghuo (Olympic flame)” (Fig. 1a-b), for whole-genome sequencing. The variety was originally developed by multiple rounds of selection/selfing of one hybrid to obtain this inbred line. This cultivar is characterized by resistance to drought and high temperatures and by improved performance related to a longer flowering period. It is well adapted to climate conditions across North China and therefore grows well in Beijing. Because of the high homozygosity obtained due to advanced generation selfing, this cultivar shows no phenotypic segregation, a characteristic of important commercial value. Seeds of this cultivar were provided by the Beijing Institute of Landscape Architecture germplasm bank.

### PacBio SMRT sequencing

High-quality high-molecular-weight genomic DNA was extracted from leaves of two soil-grown seedlings (huo1 and huo1_1) following ∼20 kb SMRTbell Libraries” protocol [11]. Plants for DNA extraction were placed in the dark for 48 hours before harvesting the leaf material. DNA was purified using the Mobio PowerClean Pro DNA Clean-Up Kit; quality was assessed using standard agarose gel electrophoresis and Thermo Fisher Scientific Qubit fluorometry. Genomic DNA was sheared to a size range of 15–40  kb using either AMPure beads (Beckman Coulte) or g-TUBE (Covaris) and enzymatically repaired and converted into SMRTbell template libraries as recommended by PacBio. Following this procedure, hairpin adapters were ligated following exonuclease-based digestion (of the remaining damaged DNA fragments and those fragments without adapters at both ends). Subsequently, the resulting SMRTbell templates were size selected using Blue Pippin electrophoresis (Sage Sciences). Templates ranging from 15 to 50  kb were sequenced on a PacBio RS II instrument using P6-C4 sequencing chemistry (25 SMRT cells for individual huo1) and on a PacBio Sequel instrument using S/P2-C2 sequencing chemistry (8 SMRT cells for the other individual, huo1_1). A total of  8,858 ,116 PacBio post-filtered reads were generated. This produced    65,962,079,028 bp (roughly 82x the assembled genome) of single-molecule sequencing data, with an average read length of 7,446 bp (Supplementary Fig. S1 and Table S1).

### Illumina short-read sequencing

DNA was extracted from leaf tissue of the same soil-grown seedlings (huo1 and huo1_1) using the Qiagen DNeasy Plant Mini Kit. Two 500-bp paired-end (PE) libraries (huo1 and huo1_1) were prepared using the NEBNext Ultra DNA Library Prep Kit for Illumina sequencing with an Illumina HiSeq X Ten machine. Short reads were processed with Trimmomatic v0.33 (Trimmomatic, RRID:SCR_011848) [12,13] and Cutadapt v1.13 (cutadapt, RRID:SCR_011841) [14,15] to remove adapter sequences and leading and trailing bases with a quality score below 20 and reads with an average per-base-quality of 20 over a 4-bp sliding window. Reads <70 nucleotides in length after trimming were removed from further analysis. A total of 265.53 million reads were generated. This produced 36.83 Gb (roughly 40x the assembled genome) of raw sequencing data, with an average cleaned read length of 137 bp (Supplementary Table S1).

### Estimation of genome size, heterozygosity, and repeat content

All generated PacBio reads were filtered and corrected with Canu v1.5 (Canu, RRID:SCR_015880) [16]; thereafter, Jellyfish (Jellyfish, RRID:SCR_005491) [17] was used to count the occurrence of k-mers based on the processed data. Finally, gce 1.0.0 [18] was used to estimate the overall characteristics of the genome, such as genome size, repeat contents, and heterozygous rate. In this study,    22,117,819,357 k-mers were generated, and the peak k-mer depth was 31 (Supplementary Fig. S2). The genome size was estimated to be approximately 711 Mb (Supplementary Table S2), and the final cleaned data corresponded to the coverage of about 33-fold. Repeat and error rates were estimated to be 47.99% and 0.27%, respectively, and the heterozygosity rate was 0.06%.

### *De novo* genome assembly

**Figure 2:**
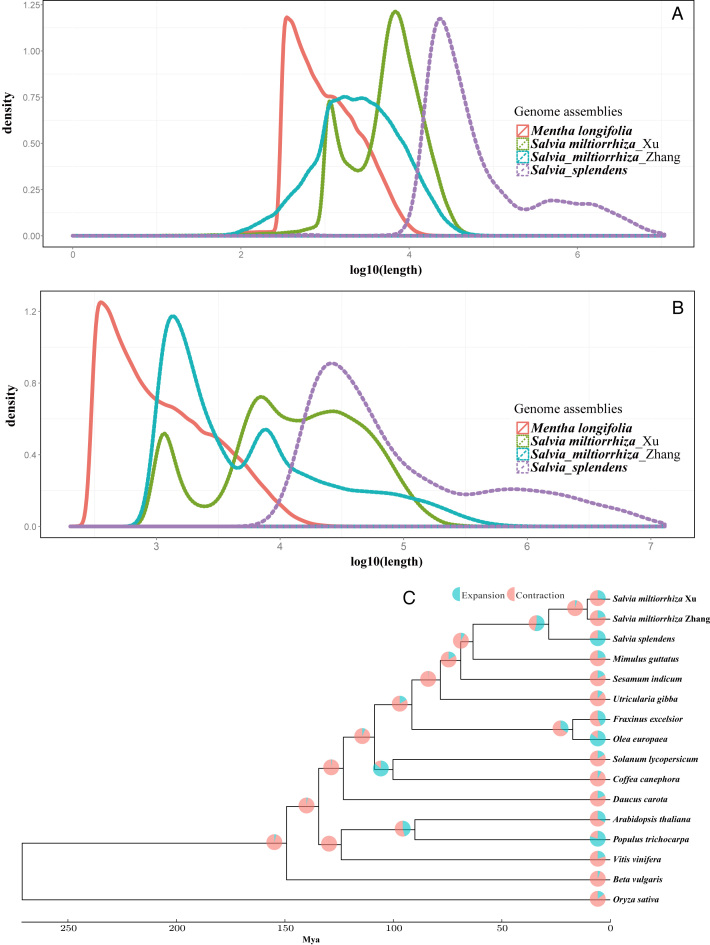
Quality of scarlet sage genome assembly and the phylogenomic inferences. Quality was assessed by comparing the scarlet genome with the recently released genomes of related species. Length distribution of contigs **(A)** and scaffolds **(B)**. **(C)** Phylogenetic tree, divergence time, and profiles of gene families that underwent expansion or contraction. *Salvia miltiorrhiza* Zhang [15] and *S. miltiorrhiza* Xu [15] are two genome assemblies reported for *S. miltiorrhiza*.

The*de novo* assembly was conducted as follows in a progressive manner. First, primary assemblies were generated from PacBio long reads of the 31 Gb from the “huo1” sequenced individual by four overlap-layout-consensus–based assemblers, Canu (produced assembly v0.1), MECAT 1.1 (assembly v0.2) [19], FALCON v0.7 (Falcon, RRID:SCR_016089) [20,21] after Canu correction (v0.3), and SMARTdenovo 1.0.0 [22] after Canu correction (v0.4) (Supplementary Table S3). Based on the size of the assembled genome, the total number of assembled contigs, N50, the L50, maximum length of the contigs, and the completeness of the genome assembly as assessed by using Benchmarking Universal Single-Copy Orthologs (BUSCO) criteria v2.0.1 (BUSCO, RRID:SCR_015008) [23] (1,440 single-copy orthologs of the Viridiplantae database) with the BLAST E-value cutoff of 10–5, the assembly (v0.1) from Canu was chosen for further polishing and scaffolding. In this selected primary assembly, the assembled genome size was 808 Mb distributed across 2,306 contigs with N50 of 2.06 Mb, L50 of 109, and maximum contig length of 8.88 Mb. We also confirmed, on average, 92.1% gene completeness in this assembly (Supplementary Table S3). In the following steps, the arrow algorithm v2.2.1 [24] was used to further improve the assembly based on PacBio long reads (v1.0), after which SSPACE-LongRead 1.1 [25] and SSPACE-standard 3.0 (SSPACE, RRID:SCR_005056) [26] were used for subsequent scaffold assembly based on PacBio long reads of 35 Gb from the second sequenced individual “hou1_1” and Illumina short reads, respectively. Finally, after scaffold processing and subsequent gap filling with SOAPdenovo and GapCloser (GapCloser, RRID:SCR_015026) [27] (v1.1), arrow v2.2.1 algorithm (based on PacBio long reads) and Pilon (Pilon, RRID:SCR_014731) (based on Illumina short reads, and run two times, parameters for Pilon: –changes –diploid –dumpreads), we obtained the final genome assembly (v1.2). Mapping of Illumina reads was done using Bowtie2 v2.3.0 (Bowtie, RRID:SCR_005476) [28]. We detected   400,170 single-nucleotide polymorphisms (SNPs), 96,854 insertions, and  62,637 deletions, respectively, for the first pilon run. Subsequently, there was a greatly decreased number of variants for the second pilon run ( 40,465 SNPs, 6,935 insertions, and 9,976 deletions, respectively). In this final assembly, we gained an assembled genome size of 808 Mb characterized by 2,204 contigs and 1,525 scaffolds (with contig N50 of 2.27 Mb and scaffold N50 of 3.12 Mb) and by gene completeness of 92.2% (Table 1 and Supplementary Table S3). This assembly represents the highest continuity and completeness among the recently released genome assemblies for the *Salvia* genus [8,9] and for mint [10], as it was examined by length distribution plotting of contigs and scaffolds, as shown in Fig. 2A, B.

**Table 1:** Statistics of the final genome assembly of the scarlet sage

	Contig		Scaffold	
	Size (bp)	Number	Size (bp)	Number
**Total size**	807,514,799	-	809,159,598	-
**Total number**	-	2,204	-	1,525
**N10**	6,529,455	10	8,157,631	9
**N50**	2,267,074	100	3,123,266	73
**N90**	265,262	456	433,303	324
**Max**.	10,812,588	-	12 , 944,193	-
**Min**.	500	-	9,495	-
**Mean**	366,386	-	530,596	-
**Median**	38,049	-	48,557	-
**Gap**	-	-	1,644,799 (0.2%)	679
**GC (Guanine Cytosine) content**	38.84%	-	38.76%	-

### DNA repeats annotation

RepeatModeler v1.0.10 (RepeatModeler, RRID:SCR_015027) [29] was used to *de novo* identify and classify repeat families in the genome assembly. Subsequently, the outputs from the RepeatModeler and RepBase [30] library were combined and used as the repeat library for subsequent RepeatMasker (RepeatMasker, RRID:SCR_012954) (v4.0.7, rmblast-2.2.28) [31] analyses, which was used to fully discover and identify repeats within the assembled genome. In summary, 57.52% of the genome was annotated as repeats, among which we found 1.08% simple repeats and 40.35% known transposable elements (TE). Long terminal repeats (LTRs) constituted the greatest proportion (26.49%) of the genome, and DNA TE made up 11.91% of the genome. Gypsy (18.15% of the genome) and Copia (7.92%) TEs were the largest components of LTRs. The results of repeat annotations are summarized in Supplementary Table S4.

### RNA sequencing, transcriptome assembly, and functional annotation

RNA was extracted from the two cultivated lines with different flower colors (red and purple) using tissue obtained from roots, shoots, leaves, calyxes, and corollas. Frozen tissue from all samples was ground manually using a mortar and pestle, and RNA was isolated using the NEBNext Poly(A) mRNA Magnetic Isolation Module. RNA quality was assessed using an Agilent 2100 BioAnalyzer. Sequencing libraries were prepared using the NEBNext Ultra RNA Library Prep Kit for Illumina; 150 bp PE sequencing was performed using an Illumina HiSeq X Ten.

A total of 1,344 million raw reads from RNA sequencing were processed by Trimmomatic and Cutadapt and aligned to the genome assembly with HiSat2 v2.1.0 (HiSat2, RRID:SCR_015530) [32]. Base quality was checked with FastQC (FastQC, RRID:SCR_014583) [33] before and after data cleaning. Respective statistics of RNA sequencing data are shown in Supplementary Table S1. Reference genome–guided transcriptome assemblies were independently prepared with Cufflinks v2.1.1 (Cufflinks, RRID:SCR_014597) [34], StringTie v1.3.3b (StringTie, RRID:SCR_016323) [35], and Trinity v2.0.6 (Trinity, RRID:SCR_013048) [36]. *De novo* assembly was generated using Trinity. Then, transcriptome assemblies were combined and further refined using CD-HIT v4.6 [37], and finally,  192,169 unique transcripts were obtained. The summary of the transcriptome assemblies is shown in Supplementary Table S5.

AUGUSTUS v3.2.3 (Augustus, RRID:SCR_008417) [38] was used for *ab initio* gene prediction, using model training based on coding sequences from *Arabidopsis thaliana* and *S. miltiorrhiza* (with two sets of proteins from independent genome annotation [8,9]). Then, transcripts from RNA sequencing were aligned to the repeat-masked reference genome assembly with BlastN and TblastX from BLAST v2.2.28+ (NCBI BLAST, RRID:SCR_004870) [39] (E-value cutoff of 10–5). Protein sequences from *A. thaliana* and *S. miltiorrhiza* were aligned to the repeat-masked reference genome assembly with BlastX (E-value cutoff of 10–5). After optimization with Exonerate v2.4.0 [40,41], gene model predictions were prepared using the MAKER package v2.31.9 (MAKER, RRID:SCR_005309) [42] provided within AUGUSTUS. To assess the quality of the gene prediction, annotation edit distance (AED) scores were generated for each of the predicted genes as part of the MAKER pipeline. The putative function for each identified gene was assessed by performing a BLAT (BLAST-like alignment tool) (BLAT, RRID:SCR_011919) [43] search of the peptide sequences against the UniProt database (UniProt, RRID:SCR_002380) [44]. Protein annotation against PFAM (Pfam, RRID:SCR_004726) [45] and InterProScan (InterProScan, RRID:SCR_005829) [46] ID were also conducted using the scripts provided in the MAKER package. The completeness of gene annotation was checked using BUSCO (1,440 single-copy orthologs of the Viridiplantae database) with a BLAST E-value cutoff of 10^−5^.

A total of 54,008 genes could be predicted, with average lengths of gene regions, genes (exons and introns), coding DNA sequence, and exons of 3,430.43 bp, 1,696.34 bp, 1,293.62 bp, and 265.94 bp, respectively (Supplementary Table S6). The comparisons among genomes from related species regarding lengths of genes, exons, and introns are shown in Fig. 2. The distribution of AED tagged by MAKER is shown in Supplementary Fig. S3, in which about 97% of the annotated genes ( 52,338 genes) had an AED <0.5 (Supplementary Table S6), indicating that the annotation is well supported. The result from BUSCO assessment of the quality of the genome assembly and annotation is shown in Supplementary Table S7. We identified 92.08% of the universal single-copy genes (1,326 genes of the total 1,440 genes), supporting the high quality of the genome assembly. Among the 1,326 BUSCO conserved single-copy genes detected in the scarlet genome, 466 genes were found to be single copies, while 860 genes were duplicated (Supplementary Table S7).

The predicted genes were annotated against several functional databases, including the NCBI nonredundant protein database (NR; [47]), the Swiss-Prot protein database [48, 44], the Translated EMBL-Bank (part of the International Nucleotide Sequence Database Collaboration, TrEMBL, [49]) [44], the protein families database (Pfam; [50]), the Cluster of Orthologous Groups for eukaryotic complete genomes (KOG) database [51], the KO (the Kyoto Encyclopedia of Genes and Genomes, Orthology) database [52,53], and Gene Ontology (GO) [54,55]. It was found that 94.67% of all predicted genes could be annotated with the following protein related databases: NR (94.60%), Swiss-Prot (63.40%), TrEMBL (93.50%), Pfam (82.10%), KOG (90.05%), KO (37.40%), and GO (78.80%) (Supplementary Table S8).

### Identification of orthologous genes and phylogenetic inference

To analyze gene families, we downloaded the protein sequences of 15 genome assemblies of 14 additional species (*Salvia miltiorrhiza* [8,9], *Fraxinus excelsior* [56], *Olea europaea* [57], *Mimulus guttatus* [58], *Utricularia gibba* [59], *Sesamum indicum* [60], *Coffea canephora* [61], *Solanum lycopersicum* [62], *Daucus carota* [63], *Vitis vinifera* [64], *Arabidopsis thaliana* [65], *Populus trichocarpa* [66], *Oryza sativa* [67], and *Beta vulgaris* [68]) (Supplementary Table S9). Orthologous and paralogous gene clusters were identified among species using OrthoMCL v2.0.9 [69]. Recommended settings were used for all-against-all BLASTP comparisons (Blast+ v2.3.056) [39] and OrthoMCL [26] analyses.

A total of  35,808 OrthoMCL families were built based on effective database sizes of all vs all BLASTP with an E-value of 10^–5^ and a Markov chain clustering default inflation parameter. We identified 1,306 gene families (3,797 genes) that were specific to the scarlet sage genome when compared with the other 15 genomes (Supplementary Table S10), and we detected  10,770 gene families that have expanded in the scarlet sage lineage using CAFE v4.0 [70,71] (Fig. 2C). The expanded gene families were enriched for 60 significant (*q*<0.05) GO terms of three functional categories, i.e., BP (Biological Process), CC (Cellular Component), and MF (Molecular Function)(Supplementary Table S11), and one KEGG (Kyoto Encyclopedia of Genes and Genomes) pathway (amino acid metabolism) (Supplementary Table S12) significant at *q* <0.05. Also, 3,579 genes and 78 gene families were detected to be contracted and found to have rapidly evolved within the scarlet sage genome (Fig. 2C). Subsequently, 134 orthologous proteins among the 16 analyzed genomes were acquired and aligned with MUSCLE v3.8.31 (MUSCLE, RRID:SCR_011812) [72] using default settings. A maximum likelihood phylogenetic tree was then generated using the concatenated amino acid sequences in PhyML v3.0 (PhyML, RRID:SCR_014629) [73] with the GTR+G+I model. The divergence time was estimated with r8s v1.81 [74] and calibrated against the timing of divergence between *A. thaliana* and *V. vinifera* (124 Mya) [75] as well as against the *A. thaliana* and *P. trichocarpa* divergence time (90 Mya) [76]. The phylogenetic analysis identified the close relationship among the three *Salvia* genomes; their divergence time was estimated to be about 28.21 Mya (Fig. 2C).

### Secondary metabolic pathways: gene annotations, gene clusters, and comparative genomics

The mint family is recognized as providing promising sources of bioactive secondary metabolites [77]. In fact, a diverse variety of bioactive secondary metabolites can be found with a wide range of pharmacological activities including antimicrobial, antispasmodic, carminative, antioxidant, antiulcer, cytoprotective, heptoprotective, cholagogue, chemo-preventive, anti-inflammatory, and antidiabetogenic. Here, we obtained enzymatic annotations for coding genes by using the E2P2 package v3.1 [78]. Then, we mapped genes to flavonoid and menthol biosynthesis pathways by querying the Plant Metabolic Network (v12.5) [79,80]. Regarding the flavonoid biosynthesis pathway, we found an abundance of genes encoding annotated enzymes in this pathway, especially of note the 41 genes for flavanone synthase I (EC: 1.14.11.9) (Supplementary Fig. S5 and Supplementary File 1). With respect to menthol biosynthesis, certain genes are still lacking annotations for enzymes such as (+)-pulegone reductase (EC: 1.3.1.81), (-)-isopiperitenone reductase (EC: 1.3.1.82), and menthol-dehydrogenase (lacking EC number) (Supplementary Fig. S6 and Supplementary File 1). However, this pathway mapping analysis provides a highly valuable reference for the genetic dissection of key metabolic genes for the scarlet sage.

The presence of metabolic gene clusters for secondary metabolic pathways is common in bacteria and filamentous fungi and is also widely reported in plants [81–83]. Using the newly created and robust computational tool kit, plantSMASH [84], we identified 85 gene clusters potentially related to secondary metabolic biosynthesis in the scarlet sage genome, as reported here, and 23 gene clusters in the *S. miltiorrhiza* genome [8]. The genomic position, gene composition, and functional annotation of the identified gene clusters are summarized in Supplementary Table S13 and Supplementary Files 2 and 3. The gene clusters were found to be potentially related to the biosynthesis of alkaloids, saccharides, polyketides, terpenes, and lignans. It was previously reported that physical clustering of terpene synthase genes (TPS) and cytochrome P450 mono-oxygenase genes is frequently associated with consecutive enzymatic actions in terpenoid biosynthesis [85]. Interestingly, we detected eight such gene clusters within the scarlet sage genome but none in the *S. miltiorrhiza* genome, which could be due, in part, to the draft status of the genome assembly for *S. miltiorrhiza*. Furthermore, significant gene co-expression across different organs was detected for one TPS gene and two of four P450 genes located in a single gene cluster (i.e., cluster 63; Supplementary Table S13 and Supplementary File 2). Evidence for moderate or significant co-expression among clustered genes was revealed and is shown in Supplementary File 2.

Based on the collinearity elucidated by former OrthoMCL analyses, a comparative genomic study between the scarlet sage and *S. miltiorrhiza* genomes revealed six pairs of gene clusters that share synteny between these two congeneric plants, and two blocks from the scarlet sage share synteny with one block from *S. miltiorrhiza* (Supplementary Fig. S7). Among the shared synteny blocks, four could be related to saccharide, one to lignan, and another to polyketide biosynthesis. The smaller number of gene clusters detected for *S. miltiorrhiza* and, subsequently, fewer shared synteny blocks of metabolic gene cluster between these two species may be partially attributed to the present state of the *S. miltiorrhiza* genome assembly, which is 100 times more fragmented than that of the scarlet sage. Thus, here, we provide a starting point for comparative genomics among plant species within the mint family.

In summary, we presented the draft assembly for the scarlet sage genome using a PacBio long-read dominated strategy that was responsible for obtaining the high-quality sequence assembly. Also, the almost complete homozygosity within the sequenced inbred line's genome was a key factor for the high continuity gained in this study. The novel genome data generated in the present study will provide a valuable resource for studying the molecular underpinnings of the various phenotypic variations found within *Salvia*sp. and sets the foundation for molecular-informed breeding strategies and genome editing approaches for this valued ornamental flowering plant. Moreover, this genome assembly is useful for comparative genomic studies among related species.

## Supplementary Material

GIGA-D-18-00028_Original_Submission.pdf

GIGA-D-18-00028_Revision_1..pdf

Response_to_Reviewer_Comments_Original_Submission.pdf

Reviewer_1_Report_(Original_Submission) -- Fritz Sedlazeck 2/21/2018 Reviewed

Reviewer_2_Report_(Original_Submission) -- Stephen Tsui 2/22/2018 Reviewed

Reviewer_2_Report_(Revision_1) -- Stephen Tsui 5/12/2018 Reviewed

Reviewer_3_Report_(Original_Submission) -- Robert van Buren 2/26/2018 Reviewed

Additional Files

## Availability of supporting data

The genome assembly, annotations, and other supporting data are available via the *GigaScience* database GigaDB [86]. The raw sequence data have been deposited in the Short Read Archive under NCBI BioProject ID PRJNA422035.

## Additional files

**Figure S1**. Length distribution of PacBio subreads.

**Figure S2**. K-mer frequency distribution at k-mer size of 17. A k-mer refers to an artificial sequence division of K nucleotides. From k-mer frequency, genomic characteristics (genome size, repeat structure and heterozygous rate) could be estimated. Peaks at depths of 31 and 62 were annotated with dash lines.

**Figure S3**. Distribution of AED scores from gene prediction. AED, Annotation Edit Distance, AED = 0 indicates perfect agreement between annotation and the evidence; AED = 1 indicates no evidence support for annotation.

**Figure S4**. Length distribution of annotated genes, exons and introns. **a-c** for annotated genes, exons and introns from different genome assemblies.

**Figure S5**. Flavonoid biosynthesis pathway. Flavonoid biosynthesis pathways by querying the Plant Metabolic Network (https://www.plantcyc.org/), enzymatic coding genes of the scarlet sage were shown for key reactions.

**Figure S6**. Menthol biosynthesis pathway. Menthol biosynthesis pathways by querying the Plant Metabolic Network (https://www.plantcyc.org/), enzymatic coding genes of the scarlet sage were shown for key reactions.

**Figure S7**. Shared synteny addressed for metabolic gene clusters between Salvia genomes. a-f: display of the different pairs of synteny blocks. Genes are colored along the contigs/scaffolds to compare between scarlet sage and *Salvia miltiorrhiza* Zhang [8], with metabolic genes highlighted with olive drab color, other homologous genes are shown in grey.

**Table S1**. Summary of PacBio and Illumina sequencing data generated in the present study. IDs of the study, sample, library and accession in NCBI SRA and sequencing platform, material origins of the sequenced DNA or RNA, the statistics of the raw and cleaned data are shown.

**Table S2**. Estimation of genome characteristics based on 17-mer statistics.

**Table S3**. Statistics of the different versions of the genome assembly of the scarlet sage. NA: data not available; * statistics for contigs/scaffolds.

**Table S4**. Summary of the annotated interspersed repeats in the genome assembly of the scarlet sage.

**Table S5**. Summary of the transcriptome assemblies.

**Table S6**. Summary of the annotated genes. AED: Annotation Edit Distance; gene regions (including UTRs, exons and introns); genes (including 5’, 3’ UTRs, exons and introns).

**Table S7**. Summary of BUSCO evaluation of gene prediction.

**Table S8**. Summary of functional annotation of predicted genes.

**Table S9**. Genomic data used for gene families analyses. Origins, download links, assembly versions, genome properties and references of 15 analyzed genomes are shown.

**Table S10**. Summary of gene family analyses. Unique groups and genes, single-copy and duplicated groups and genes are summarized for the 16 analyzed genomes of 15 plant species.

**Table S11**. GO enrichment of expanded gene families. (A) “Category” is the Gene Ontology (GO) term ID; (B) “p_value” is the over represented *P*-value indicating the observed frequency of a given term among analyzed genes is equal to the expected frequency based on the null distribution; i.e., lower *P*-values indicate stronger evidence for overrepresentation; (C) “q_value” is the Benjamini and Hochberg adjusted *P*-value, (D) “numEPInCat” is the number of expanded gene families in the corresponding GO category; (E) “numInCat” is the number of detected gene families in the corresponding GO category; (F) “Term” is the GO term; (G) “Ontology” indicates which ontology the term comes from. 60 significant (q<0.05) GO-terms of three different functional categories are indicated in bold.

**Table S12**. KEGG enrichment of expanded gene families. (A) “KO category” is the KEGG Orthology (KO) category ID; (B) “p_value” is the over represented *P*-value indicating the observed frequency of a given term among analyzed genes is equal to the expected frequency based on the null distribution; i.e., lower *P*-values indicate stronger evidence for overrepresentation; (C) “q_value” is the Benjamini and Hochberg adjusted *P*-value, (D) “numEPInCat” is the number of expanded gene families in the corresponding KO category; (E) “numInCat” is the number of detected gene families in the corresponding KO category; (F) “Pathway” is the KEGG pathway; (G) “Class” indicates which KEGG class the pathway comes from. One significant (q<0.05) KEGG pathway is indicated in bold.

**Table S13**. Summary of metabolic gene clusters detected in genomes of *Salvia miltiorrhiza* and *S. splendens*. (A) “Genome” denotes the genome origination; (B) “Cluster” is the code for a certain gene cluster detected; (C) “Record” denotes the contig/scaffold ID from where the gene cluster was detected; (D) “Type” denotes the functional assignment for the gene cluster; (E) “From”, “To” and “Size” denote the genomic position and range of the gene cluster; (F) “Core domains” denote the domain annotation for the metabolic genes in the cluster; (G) “CD-HIT Cluster” indicate the number of genes in the cluster; (H) “Gene cluster genes” is showing the ID of genes in the cluster.

**Supplementary File 1**. Genes (Gene ID, name and EC number) mapped to flavonoid and menthol biosynthesis pathways.

**Supplementary File 2**. Structure of a metabolic gene cluster (polyketide synthesis) and gene expression patterns of *Salvia splendens*. Genomic position, gene composition, functional annotation of gene cluster are shown, also including a heatmap of tissue specific expression of the genes within the presented cluster is shown. HG: root of red flower (individual); HJ: stem of red flower (individual); HY: leave of red flower (individual); HE: calyx of red flower (individual); HHG: corolla of red flower (individual); ZG: root of purple flower (individual); ZJ: stem of purple flower (individual); ZY: leave of purple flower (individual); ZE: calyx of purple flower (individual); ZHG: corolla of purple flower (individual).

**Supplementary File 3**. Structure of a metabolic gene cluster (alkaloid synthesis). Genomic position, gene composition, functional annotation of gene cluster were shown.

## Abbreviations

AED: annotation edit distance; BUSCO: Benchmarking Universal Single-Copy Orthologs; GO: Gene Ontology; LTR: long terminal repeat; Mya: million years ago; NCBI: National Center for Biotechnology Information; PE: paired end; SMRT: single-molecule real-time; SNP: single-nucleotide polymorphism; TE: transposable element; TSP: terpene synthase genes.
