# Supplementary material for: High-quality assembly of the reference genome for scarlet sage, Salvia splendens, an economically important ornamental plant
Source: Gigascience. 2018 Jun 19;7(7):giy068. doi: 10.1093/gigascience/giy068 (PMC6030905; doi:10.1093/gigascience/giy068)
Supplement: GIGA-D-18-00028_Original_Submission.pdf [file giy068_giga-d-18-00028_original_submission.pdf]

GigaScience

High quality assembly of the reference genome for scarlet sage, Salvia splendens, an economically important ornamental plant

--Manuscript Draft--

**Manuscript Number:**	GIGA-D-18-00028
**Full Title:**	High quality assembly of the reference genome for scarlet sage, Salvia splendens, an economically important ornamental plant
**Article Type:**	Data Note
**Funding Information:**	Beijing Key Laboratory of Green Plants Breeding — Dr. Ri-Chen Cong<br>Fundamental Research Funds for the Central Universities (YX2013-41) — Mr. Jian-Feng Mao
**Abstract:**	Background: Salvia splendens Ker-Gawler, scarlet or tropical sage, is a tender herbaceous perennial widely introduced and seen in public gardens all over the world. With few molecular resources, breeding is still restricted to traditional phenotypic selection, and the genetic mechanisms underlying phenotypic variation still remain unknown. Hence, a high quality reference genome will be very valuable for marker assisted breeding, genome editing or molecular genetics.<br>Findings: We generated 66 gigabases (Gb) and 37 Gb of raw DNA sequences, respectively, from whole-genome sequencing of a largely homozygous scarlet sage inbred line using PacBio Single-Molecule Real-Time (SMRT) and Illumina HiSeq sequencing platforms. PacBio de novo assembly yielded a final genome with a scaffold N50 size of 3.12 megabases (Mb), and a total length of 808 Mb. The repetitive sequences identified accounted for 57.52% of the genome sequence and 54,008 protein-coding genes were predicted collectively with ab initio and homology-based gene prediction from the masked genome. The divergence time between S. splendens and S. miltiorrhiza was estimated with 28.21 million years ago (Mya). Moreover, 3,797 species-specific genes and 1,187 expanded gene families were identified for the scarlet sage genome.<br>Conclusions: We provide the first genome sequence and gene annotation for the scarlet sage. The availability of these resources will be of great importance for further breeding strategies, genome editing and also for comparative genomics among related species.
**Corresponding Author:**	Ri-Chen Cong, Ph.D<br>Beijing Institute of Landscape Architecture<br>Beijing, CHINA
**Corresponding Author Secondary Information:**	
**Corresponding Author's Institution:**	Beijing Institute of Landscape Architecture
**Corresponding Author's Secondary Institution:**	
**First Author:**	Jian-Feng Mao, Ph.D.
**First Author Secondary Information:**	
**Order of Authors:**	Jian-Feng Mao, Ph.D.
	Ai-Xiang Dong
	Hai-Bo Xin
	Zi-Jing Li
	Hui Liu
	Yan-Qiang Sun
	Shuai Nie

Powered by Editorial Manager® and ProduXion Manager® from Aries Systems Corporation

	Zheng-Nan Zhao
	Rong-Feng Cui
	Hua-Li Zhang
	Ren-Gang Zhang
	Quan-Zheng Yun
	Fatemeh Maghuly
	Ilga Porth
	Ri-Chen Cong
**Order of Authors Secondary Information:**	
**Opposed Reviewers:**	
**Additional Information:**	
**Question**	**Response**
Are you submitting this manuscript to a special series or article collection?	No
**Experimental design and statistics**<br><br>Full details of the experimental design and statistical methods used should be given in the Methods section, as detailed in our Minimum Standards Reporting Checklist. Information essential to interpreting the data presented should be made available in the figure legends.<br><br>Have you included all the information requested in your manuscript?	Yes
**Resources**<br><br>A description of all resources used, including antibodies, cell lines, animals and software tools, with enough information to allow them to be uniquely identified, should be included in the Methods section. Authors are strongly encouraged to cite Research Resource Identifiers (RRIDs) for antibodies, model organisms and tools, where possible.<br><br>Have you included the information requested as detailed in our Minimum Standards Reporting Checklist?	Yes
**Availability of data and materials**<br><br>All datasets and code on which the conclusions of the paper rely must be either included in your submission or	Yes

deposited in publicly available repositories (where available and ethically appropriate), referencing such data using a unique identifier in the references and in the “Availability of Data and Materials” section of your manuscript.<br><br>Have you have met the above requirement as detailed in our Minimum Standards Reporting Checklist?	

Manuscript
Click here to download Manuscript Salvia_Genome_paper_mao7_Ilga1-4.docx

High quality assembly of the reference genome for scarlet sage, *Salvia splendens*, an economically important ornamental plant

Ai-Xiang Dong[1‡], Hai-Bo Xin[1‡], Zi-Jing Li[1‡], Hui Liu[2‡], Yan-Qiang Sun[2‡], Shuai Nie[2], Zheng-Nan Zhao[1], Rong-Feng Cui[1], Hua-Li Zhang[1], Ren-Gang Zhang[3], Quan-Zheng Yun[3], Fatemeh Maghuly[4], Ilga Porth[5], Ri-Chen Cong[1*], Jian-Feng Mao[2*]

[1] Beijing Key Laboratory of Green Plants Breeding, Beijing Institute of Landscape Architecture, Beijing, 10020, China.

[2] Beijing Advanced Innovation Center for Tree Breeding by Molecular Design, National Engineering Laboratory for Tree Breeding, Key Laboratory of Genetics and Breeding in Forest Trees and Ornamental Plants, Ministry of Education, College of Biological Sciences and Technology, Beijing Forestry University, Beijing, 100083, China.

[3] Beijing Ori-Gene Science and Technology Co. Ltd, Beijing, 10226, China.

[4] Plant Biotechnology Unit (PBU), Dept. Biotechnology, BOKU-VIBT, University of Natural Resources and Life Sciences, Muthgasse 18, 1190 Vienna, Austria.

[5] Département des sciences du bois et de la forêt, Pavillon Charles-Eugène-Marchand, 1030, Avenue de la Médecine, Université Laval, Québec (Québec) G1V 0A6, Canada.

‡These authors contributed equally to this paper.

*Correspondence to: hardhopeee@163.com (RCC); jianfeng.mao@bjfu.edu.cn (JFM)

Abstract

Background: *Salvia splendens* Ker-Gawler, scarlet or tropical sage, is a tender herbaceous perennial widely introduced and seen in public gardens all over the world. With few molecular resources, breeding is still restricted to traditional phenotypic selection, and the genetic mechanisms underlying phenotypic variation still remain unknown. Hence, a high quality reference genome will be very valuable for marker assisted breeding, genome editing or molecular genetics.

Findings: We generated 66 gigabases (Gb) and 37 Gb of raw DNA sequences, respectively, from whole-genome sequencing of a largely homozygous scarlet sage inbred line using PacBio Single-Molecule Real-Time (SMRT) and Illumina HiSeq sequencing platforms. PacBio *de novo* assembly yielded a final genome with a scaffold N50 size of 3.12 megabases (Mb), and a total length of 808 Mb. The repetitive sequences identified accounted for 57.52% of the genome sequence and 54,008 protein-coding genes were predicted collectively with *ab initio* and homology-based gene prediction from the masked genome. The divergence time between *S. splendens* and *S. miltiorrhiza* was estimated with 28.21 million years ago (Mya). Moreover, 3,797 species-specific genes and 1,187 expanded gene families were identified for the scarlet sage genome.

Conclusions: We provide the first genome sequence and gene annotation for the scarlet sage. The availability of these resources will be of great importance for further breeding strategies, genome editing and also for comparative genomics among related species.

Keywords: annotation, evolution, reference genome, *Salvia splendens*, scarlet sage

Data description

Background information

Salvia L., with nearly 1,000 species of shrubs, herbaceous perennials, and annuals, is the largest genus in the mint family (Lamiaceae: Nepetoideae: Mentheae: Salviinae) [1-4]. The genus is widely distributed throughout the world. Many species of this genus are extensively used for culinary purposes, essential oil production and Chinese herbal remedies such as the two species *S. officinalis* [3] and *S. miltiorrhiza* (Danshen). Additionally, they are used as ornamental plants valued for their flowers or for their aromatic foliage such as *S. splendens* (**Fig. 1 a-k**).

S. splendens, scarlet or tropical sage, is a herbaceous perennial species, which is native to Brazil. While it is a perennial in warmer climate zones, it grows as an annual in cooler areas. *S. splendens* is a very popular bedding plant, and is widely introduced in public gardens all over the world [3,5], characterized by its dense flowers, and wide variation of colours (scarlet, purple, pink, blue, lavender, salmon, yellow green, white and bicolor), as well as long lasting flowering (3-9 weeks or even longer). Additionally, *S. splendens* can provide outstanding visual effects when grown in beds, borders and containers with long-lasting lifespans ranging from late spring to first frost occurrence. Furthermore, the flower is easy to maintain and fairly free of pests and diseases due to Lamiaceae’s characteristic insect repellent fragrance content [6]. The plant blends nicely with other annuals or perennial plants for the best visual effects in an ensemble

setting; in addition this plant requires little deadheading as well it attracts various butterfly species. *S. splendens* is a prolific and durable bloomer, thrives in full sun, and survives in a large range of soil moisture regimes.

Traditional breeding activities using phenotypic selection as well as performing targeted variety hybridizations between elite cultivars have resulted in a large number of new cultivars with different performances regarding flowering characters (related to colour, flowering time, flowering period amongst others), individual growth performance, height, and/or tolerance to moisture or temperature extremes. However, little is known about the molecular mechanisms underlying such economically important characteristics for ornamental varieties. To date, only few genetic markers [7] are available for marker assistant breeding or genetic modification.

In the current study, we present the first high quality genome assembly for *S. splendens* with a hybrid assembly strategy using PacBio Single-Molecule Real-Time and Illumina HiSeq short-read sequencing platforms. The genome assembly, its structural and functional annotation, provide a valuable reference for the genomic dissection of the phenotypic variation in *Salvia*, and new breeding strategies. This reference genome could also be used in comparative genomics with the recently released *Salvia* genome (*S. miltiorrhiza*) [8,9] and the mint genome (*Mentha longifolia*) [10] to study the biosynthesis of important fragrant and medicinal compounds.

Plant material

We chose the elite variety *S. splendens*, "Aoyunshenghuo (Olympic flame)" (**Fig. 1 a-b**) for whole genome sequencing, which was originally developed by multiple rounds of selection/selfing of one hybrid to obtain this inbred line. This cultivar is characterized by resistance to drought, high temperature, and improved performance related to a longer flowering period; it is well adapted to climate conditions across North China, and therefore grows well in Beijing. Because of the high homozygosity obtained due to advanced generation selfing, this cultivar shows no phenotypic segregation, a characteristic of important commercial value. Seeds of this cultivar were provided by the Beijing Institute of Landscape Architecture germplasm bank.

PacBio SMRT sequencing

High quality high molecular weight genomic DNA was extracted from leaves of two soil-grown seedlings (huo1 and huo1_1) following ~20 kb SMRTbell™ Libraries" protocol (http://www.pacb.com/wp-content/uploads/2015/09/Shared-Protocol-Preparing-Arabidopsis-DNA-for-20-kb-SMRTbell-Libraries.pdf). Plants for DNA extraction have been placed in the dark for 48 h before harvesting the leaf material. DNA was purified with Mobio PowerClean® Pro DNA Clean-Up Kit and quality was assessed by standard agarose gel electrophoresis and Thermo Fisher Scientific Qubit Fluorometry. Genomic DNA was sheared to a size range of 15–40 kb using either AMPure beads (Beckman Coulte) or g-TUBE (Covaris), and enzymatically repaired and converted into SMRTbell template libraries as recommended by Pacific

Biosciences. Following this procedure, hairpin adapters were ligated following exonucleasese-based digestion (of the remaining damaged DNA fragments and those fragments without adapters at both ends). Subsequently, the resulting SMRTbell templates were size-selected by Blue Pippin electrophoresis (Sage Sciences) and templates ranging from 15 to 50 kb were sequenced on a PacBio RS II instrument using P6-C4 sequencing chemistry (25 Single-Molecule Real-Time (SMRT) cells for individual huo1) and on a PacBio Sequel instrument using S/P2-C2 sequencing chemistry (8 SMRT cells for the other individual, huo1_1). A total of 8,858,116 PacBio post-filtered reads were generated. This produced a total of 65,962,079,028 bp (roughly 82x of the assembled genome) of single-molecule sequencing data, with an average read length of 7,446 bp (**Fig. S1** and **Table S1)**.

Illumina short-read sequencing

DNA was extracted from leaf tissue of the same soil-grown seedlings (huo1 and huo1_1) using the Qiagen DNeasy Plant Mini Kit. Two 500 bp paired-end (PE) libraries (huo1 and huo1_1) were prepared using the NEBNext Ultra DNA Library Prep Kit for Illumina sequencing with an Illumina HiSeq X Ten machine. Short reads were processed with Trimmomatic (v0.33) [11] and Cutadapt (v1.13) [12] to remove adapter sequences and leading and trailing bases with a quality score below 20 and reads with an average per-base-quality of 20 over a 4 bp sliding window. Reads < 70 nucleotides in length after trimming were removed from further analysis. A total of 265.53 million

reads were generated. This produced a total of 36.83 Gb (roughly 40x of the assembled genome) of raw sequencing data, with an average cleaned read length of 137 bp (**Table S1**).

Estimation of genome size, heterozygosity, and repeat content

All generated PacBio reads were filtered and corrected with Canu [13], thereafter, Jellyfish [14] was used to count occurrence of k-mers based on the processed data. Finally, gce [15] was employed to estimate the overall characteristics of the genome such as genome size, repeat contents and heterozygous rate. In this study, a total of 22,117,819,357 k-mers were generated and the peak k-mer depth was 31 (**Fig. S2**). The genome size was estimated to be approximately 711 Mb (**Table S2**) and the final cleaned data corresponded to the coverage of about 33-fold. Repeat and error rates were estimated to be 47.99% and 0.27%, respectively, and heterozygosity rate was 0.06%.

***De novo* genome assembly**

De novo assembly was conducted as follows in a progressive manner. Firstly, primary assemblies were generated from PacBio long reads by four different Overlap-Layout-Consensus (OLC) based assemblers, Canu (produced assembly v0.1) [13], MECAT (assembly v0.2) [16], FALCON [17] (https://github.com/PacificBiosciences/FALCON/) after Canu correction (v0.3) and SMARTdenovo (https://github.com/ruanjue/smartdenovo) after Canu correction (v0.4)

(**Table S3**). Based on the size of the assembled genome, the total number of assembled contigs, N50, the L50, maximum length of the contigs, and also the completeness of the genome assembly as assessed by using BUSCO criteria [18] (956 single copy orthologs of the Viridiplantae database) with the BLAST E-value cutoff of 10^{-5}, assembly (v0.1) from Canu was chosen for further polishing and scaffolding. In this selected primary assembly, the assembled genome size was 808 Mb distributed across 2,306 contigs with N50 of 2.06 Mb, L50 of 109 and maximum contig length of 8.88 Mb. We also confirmed on average 92.1% gene completeness in this assembly (**Table S3**). In the following steps, the arrow algorithm (https://github.com/PacificBiosciences/GenomicConsensus) was used to further improve the assembly based on PacBio long reads (v1.0), after which SSPACE-LongRead [19] and SSPACE-standard [20] were used for subsequent scaffold assembly based on PacBio long reads and Illumina short reads, respectively. Finally, after scaffold processing and subsequent gap filling with SOAPdenovo and GapCloser [21] (v1.1), arrow algorithm (based on PacBio long reads) and pilon (based on Illumina short reads, and run two times), we got the final genome assembly (v1.2). In this final assembly, we gained an assembled genome size of 808 Mb characterized by 2,204 contigs and 1,525 scaffolds (with contig N50 of 2.27 Mb and scaffold N50 of 3.12 Mb), and by gene completeness of 92.2% (**Table 1 and Table S3**). This assembly represents the highest continuity and completeness among the recently released genome assemblies for the *Salvia* genus [8,9] and the mint family [10], as it was examined by length distribution

plotting of contigs and scaffolds as shown in **Fig. 2a, b**.

DNA repeats annotation

RepeatModeler (v1.0.10) (http://www.repeatmasker.org/RepeatModeler.html) was employed to *de novo* identify and classify repeat families in the genome assembly. Subsequently, the outputs from RepeatModeler and RepBase [22] library were combined and used as repeat library for subsequent RepeatMasker (v4.0.7, rmblast-2.2.28) (http://www.repeatmasker.org/) analyses, which was used to fully discover and identify repeats within the assembled genome. In summary, 57.52% of the genome was annotated as repeats among which we found 1.08% simple repeats and 40.35% known transposable elements (TE). Long terminal repeats (LTRs) constituted the greatest proportion, 26.49% of the genome, and DNA TE made up 11.91% of the genome. Gypsy (18.15% of the genome) and Copia (7.92%) TEs were the largest components of LTRs. The results of repeat annotations are summarized in **Table S4**.

RNA sequencing, transcriptome assembly and functional annotation

RNA was extracted from the two cultivated lines with different flower colours (red and purple) using tissue obtained from, roots, shoots, leaves, calyxes and corollas. Frozen tissue from all samples was ground manually using mortar and pestil, and RNA was isolated using the NEBNext Poly(A) mRNA Magnetic Isolation Module. RNA quality was assessed using an Agilent 2100 BioAnalyzer. Sequencing libraries were prepared

using the NEBNext Ultra RNA Library Prep Kit for Illumina. 150 bp PE sequencing was performed using an Illumina HiSeq X Ten.

1,344 million raw reads from RNA sequencing were processed by Trimmomatic and Cutadapt and aligned to the genome assembly with HiSat2 [23]. Base quality was checked with FastQC (https://www.bioinformatics.babraham.ac.uk/projects/fastqc/) before and after data cleaning, and respective statistics of RNA sequencing data are shown in **Table S1**. Reference genome guided transcriptome assemblies were independently prepared with Cufflinks [24], StringTie [25] and Trinity [26]. *De novo* assembly was generated using Trinity, then, transcriptome assemblies were combined and further refined using CD-HIT [27], and finally, 192,169 unique transcripts were gained. The summary of the transcriptome assemblies is shown in **Table S5**.

AUGUSTUS [28] was employed for *ab initio* gene prediction, using model training based on coding sequences from *Arabidopsis thaliana* and *S. miltiorrhiza* (with two sets of proteins from independent genome annotation [8, 9]). Then, transcripts from RNA sequencing were aligned to the repeat-masked reference genome assembly with BlastN and TblastX [29] (E-value cutoff of 10^{-5}), and protein sequences from *A. thaliana* and *S. miltiorrhiza* were aligned to the repeat-masked reference genome assembly with BlastX (E-value cutoff of 10^{-5}). After optimization with Exonerate (https://www.ebi.ac.uk/about/vertebrate-genomics/software/exonerate) [30], gene model predictions were finalized prepared using the MAKER package [31] provided within AUGUSTUS. To assess the quality of the gene prediction, AED (Annotation

Edit Distance) scores were generated for each of the predicted genes as part of the MAKER pipeline. Putative function for each identified gene was assessed by performing a BLAT [32] search of the peptide sequences against the Uniprot database [33], Protein annotation against PFAM [34] and InterProScan [35] ID were also conducted using the scripts provided in the MAKER package. Completeness of gene annotation was checked using BUSCO (956 single copy orthologs of the Viridiplantae database) with a BLAST E-value cutoff of 10^{-5}.

54,008 genes could be predicted, with average lengths of gene regions, genes (including 5’, 3’ UTRs, exons and introns), CDS and exons of 3,430.43 bp, 1696.34 bp, 1293.62 bp and 265.94 bp, respectively (**Table S6**). The comparisons among genomes from related species regarding lengths of genes, exons, and introns are shown in **Fig. 2**. The distribution of AED tagged by MAKER is shown in **Fig. S3**, in which about 97% of the annotated genes (52,338 genes) had an AED < 0.5 (**Table S6**), thus indicating that the annotation is well supported. The result from BUSCO assessment of the quality of the genome assembly and annotation is shown in **Table S7**. 92.08 % of the universal single-copy genes (1,326 genes out of the total 1,440 genes) were identified, supporting the high quality of the genome assembly. Among the 1,326 BUSCO conserved single-copy genes detected in the scarlet genome, 466 genes were found single-copy, while 860 genes were duplicated (**Table S7**).

The predicted genes were annotated against several functional databases, including: (1) the NCBI non-redundant protein database (Nr;

http://www.ncbi.nlm.nih.gov), (2) Swiss-Prot protein database (http://www.expasy.ch/sprot) [33], (3) Translated EMBL-Bank (part of the International Nucleotide Sequence Database Collaboration, TrEMBL, http://www.ebi.ac.uk/uniprot) [33], (4) the protein families database (Pfam; http://pfam.xfam.org/), (5) Cluster of Orthologous Groups for eukaryotic complete genomes (KOG) database (http://genome.jgi-psf.org/help/kogbrowser.jsf), (6) KO (the Kyoto Encyclopedia of Genes and Genomes, Orthology) database (http://www.genome.jp/kegg/ko.html) [36], and (7) Gene ontology (GO) (http://www.geneontology.org) [37]. 94.67 % of all predicted genes could be annotated with the following protein related databases: NR (94.60 %), Swiss-Prot (63.40 %), TrEMBL (93.50 %), Pfam (82.10 %), KOG (90.05 %), KO (37.40 %), and GO (78.80 %) (**Table S8**).

Identification of orthologous genes and phylogenetic inference

To analyze gene families, we downloaded the protein sequences of 15 additional species (*Salvia miltiorrhiza* [8, 9], *Fraxinus excelsior* [38], *Olea europaea* [39], *Mimulus guttatus* [40], *Utricularia gibba* [41], *Sesamum indicum* [42], *Coffea canephora* [43], *Solanum lycopersicum* [44], *Daucus carota* [45], *Vitis vinifera* [46], *Arabidopsis thaliana* [47], *Populus trichocarpa* [48], *Oryza sativa* [49] and *Beta_vulgaris* [50]) (**Table S9**). Orthologous and paralogous gene clusters were identified among species using OrthoMCL [51]. Recommended settings were used for all-against-all BLASTP

comparisons (Blast+ v2.3.056) [29] and OrthoMCL [51] analyses.

A total of 35,808 OrthoMCL families were built based on effective database sizes of all versus all BLASTP with an E-value of 10^{-5} and a Markov Chain Clustering default inflation parameter. We identified 1,306 gene families (3,797 genes) that were specific to the scarlet sage genome when comparing with the other 15 genomes (**Table S10**), and we detected 10,770 gene families that have expanded in the scarlet sage lineage, using CAFE [52] (**Fig. 2c**). The expanded gene families were enriched for 60 significant (q<0.05) GO-terms of three different functional categories, i.e. BP, CC, and MF (**Table S11**) and one KEGG pathway (amino acid metabolism) (**Table S12**) significant at q<0.05. Also, 3,579 genes and 78 gene families were detected to be contracted and found to have rapidly evolved within the scarlet sage genome (**Fig. 2c**). Subsequently, 134 orthologous proteins among the 16 analyzed genomes were acquired and aligned with MUSCLE v3.8.31 [53] employing default settings. A maximum likelihood phylogenetic tree was then generated using the concatenated amino acid sequences in PhyML 3.0 [54] with GTR+G+I model. The divergence time was estimated with r8s [55] and calibrated against the timing of divergence between *A. thaliana* and *V. vinifera* (124 Mya) [56] as well as against *A. thaliana* and *P. trichocarpa* divergence time (90 Mya) [57]. The phylogenetic analysis identified the close relationship among the three *Salvia* genomes and their divergence time was estimated with about 28.21 Mya (**Fig. 2c**)

In summary, we presented the draft assembly for the scarlet sage genome using a

PacBio long-read dominated strategy, which was responsible for obtaining the high sequence assembly quality. Also, the almost complete homozygosity within the sequenced inbred line's genome was a key factor for the high continuity gained in this study. The novel genome data generated in the present study will provide a valuable resource for studying the molecular underpinnings of the various phenotypic variation found within *Salvia sp.*, and sets the foundation for molecular-informed breeding strategies and genome editing approaches for this valued ornamental flowering plant. Moreover, this genome assembly is useful for comparative genomic studies among related species.

Availability of supporting data

The genome assembly, annotations, and other supporting data are available via the GigaScience database GigaDB. The raw sequence data have been deposited in the Short Read Archive (SRA) under NCBI BioProject ID PRJNA422035.

Abbreviations

bp: base pair; kb: kilobases; Mb: megabases; Gb: gigabases; TE: transposable element; BUSCO: benchmarking universal single-copy orthologs; CDS: coding sequence.

Acknowledgement

This study was funded by Beijing Key Laboratory of Green Plants Breeding and

Fundamental Research Funds for the Central Universities (NO.YX2013-41).

Author Contributions

AXD, HBX, RCC, JFM, FM and IP conceived and designed the study; AXD, HBX, ZJL, HL, YQS, SN, ZNZ, RFC, HLZ, RGZ and QZY prepared the materials and conducted the experiments; JFM, HBX, FM, IP wrote the manuscript.

Conflict of Interest

The authors declare that they have no competing financial interests.

References

1. Drew BT, González-Gallegos JG, Xiang C-L, Kriebel R, Drummond CP, Walker JB, et al. Salvia united: The greatest good for the greatest number. Taxon. 2017;66 1:133-45. doi:10.12705/661.7.
2. Sutton J. The Gardener's Guide to Growing Salvias. David & Charles; 1999.
3. Clebsch B and Barner CD. The New Book of Salvias: Sages for Every Garden. Timber Press; 2003.
4. Walker JB, Sytsma KJ, Treutlein J and Wink M. Salvia (Lamiaceae) is not monophyletic: implications for the systematics, radiation, and ecological specializations of *Salvia* and tribe Mentheae. American Journal of Botany. 2004;91 7:1115-25. doi:10.3732/ajb.91.7.1115.
5. Griffiths M and Society RH. Index of Garden Plants. Macmillan; 1994.
6. Regnault-Roger C. The potential of botanical essential oils for insect pest control. Integrated Pest Management Reviews. 1997;2 1:25-34. doi:10.1023/a:1018472227889.
7. Ge X, Chen H, Wang H, Shi A and Liu K. De novo assembly and annotation of salvia splendens transcriptome using the Illumina platform. PLoS One. 2014;9 3:e87693. doi:10.1371/journal.pone.0087693.
8. Zhang G, Tian Y, Zhang J, Shu L, Yang S, Wang W, et al. Hybrid de novo genome assembly of the Chinese herbal plant danshen (*Salvia miltiorrhiza* Bunge). GigaScience. 2015;4 1:62. doi:10.1186/s13742-015-0104-3.

9. Xu H, Song J, Luo H, Zhang Y, Li Q, Zhu Y, et al. Analysis of the Genome Sequence of the Medicinal Plant *Salvia miltiorrhiza*. Molecular Plant. 2016;9 6:949-52. doi:10.1016/j.molp.2016.03.010.
10. Vining KJ, Johnson SR, Ahkami A, Lange I, Parrish AN, Trapp SC, et al. Draft Genome Sequence of *Mentha longifolia* and Development of Resources for Mint Cultivar Improvement. Molecular Plant. 2017;10 2:323-39. doi:10.1016/j.molp.2016.10.018.
11. Bolger AM, Lohse M and Usadel B. Trimmomatic: a flexible trimmer for Illumina sequence data. Bioinformatics. 2014;30 15:2114-20. doi:10.1093/bioinformatics/btu170.
12. Martin M. Cutadapt removes adapter sequences from high-throughput sequencing reads. EMBnetjournal. 2011;17 1 doi:10.14806/ej.17.1.200 pp. 10-12.
13. Koren S, Walenz BP, Berlin K, Miller JR, Bergman NH and Phillippy AM. Canu: scalable and accurate long-read assembly via adaptive k-mer weighting and repeat separation. Genome Research. 2017; doi:10.1101/gr.215087.116.
14. Marçais G and Kingsford C. A fast, lock-free approach for efficient parallel counting of occurrences of k-mers. Bioinformatics. 2011;27 6:764-70. doi:10.1093/bioinformatics/btr011.
15. Liu B, Shi Y, Yuan J, Hu X, Zhang H, Li N, et al. Estimation of genomic characteristics by analyzing k-mer frequency in *de novo* genome projects. arXiv preprint arXiv:13082012. 2013.
16. Xiao C-L, Chen Y, Xie S-Q, Chen K-N, Wang Y, Han Y, et al. MECAT: fast mapping, error correction, and de novo assembly for single-molecule sequencing reads. Nature Methods. 2017;14:1072. doi:10.1038/nmeth.4432.
17. Chin CS, Peluso P, Sedlazeck FJ, Nattestad M, Concepcion GT, Clum A, et al. Phased diploid genome assembly with single-molecule real-time sequencing. Nature Methods. 2016;13 12:1050-4. doi:10.1038/nmeth.4035.
18. Simão FA, Waterhouse RM, Ioannidis P, Kriventseva EV and Zdobnov EM. BUSCO: assessing genome assembly and annotation completeness with single-copy orthologs. Bioinformatics. 2015;31 19:3210-2.
19. Boetzer M and Pirovano W. SSPACE-LongRead: scaffolding bacterial draft genomes using long read sequence information. BMC Bioinformatics. 2014;15 1:211. doi:10.1186/1471-2105-15-211.
20. Boetzer M, Henkel CV, Jansen HJ, Butler D and Pirovano W. Scaffolding pre-assembled contigs using SSPACE. Bioinformatics. 2011;27 4:578-9. doi:10.1093/bioinformatics/btq683.
21. Luo R, Liu B, Xie Y, Li Z, Huang W, Yuan J, et al. SOAPdenovo2: an empirically improved memory-efficient short-read *de novo* assembler. GigaScience. 2012;1:18-. doi:10.1186/2047-217X-1-18.
22. Bao W, Kojima KK and Kohany O. Repbase Update, a database of repetitive elements in eukaryotic genomes. Mobile DNA. 2015;6 1:11.

doi:10.1186/s13100-015-0041-9.

23. Kim D, Langmead B and Salzberg SL. HISAT: a fast spliced aligner with low memory requirements. Nature Methods. 2015;12 4:357-60.

24. Trapnell C, Williams BA, Pertea G, Mortazavi A, Kwan G, van Baren MJ, et al. Transcript assembly and quantification by RNA-Seq reveals unannotated transcripts and isoform switching during cell differentiation. Nature Biotechnology. 2010;28 5:511-5. doi:10.1038/nbt.1621.

25. Pertea M, Pertea GM, Antonescu CM, Chang T-C, Mendell JT and Salzberg SL. StringTie enables improved reconstruction of a transcriptome from RNA-seq reads. Nature Biotechnology. 2015;33 3:290-5.

26. Grabherr MG, Haas BJ, Yassour M, Levin JZ, Thompson DA, Amit I, et al. Full-length transcriptome assembly from RNA-Seq data without a reference genome. Nature Biotechnology. 2011;29:644. doi:10.1038/nbt.1883.

27. Fu L, Niu B, Zhu Z, Wu S and Li W. CD-HIT: accelerated for clustering the next-generation sequencing data. Bioinformatics. 2012;28 23:3150-2.

28. Stanke M, Diekhans M, Baertsch R and Haussler D. Using native and syntenically mapped cDNA alignments to improve *de novo* gene finding. Bioinformatics. 2008;24 5:637-44. doi:10.1093/bioinformatics/btn013.

29. Boratyn GM, Schäffer AA, Agarwala R, Altschul SF, Lipman DJ and Madden TL. Domain enhanced lookup time accelerated BLAST. Biology Direct. 2012;7 1:12. doi:10.1186/1745-6150-7-12.

30. Slater GSC and Birney E. Automated generation of heuristics for biological sequence comparison. BMC Bioinformatics. 2005. doi:10.1186/1471-2105-6-31.

31. Cantarel BL, Korf I, Robb SM, Parra G, Ross E, Moore B, et al. MAKER: an easy-to-use annotation pipeline designed for emerging model organism genomes. Genome Resesearch. 2008;18 1:188-96. doi:10.1101/gr.6743907.

32. Kent WJ. BLAT--the BLAST-like alignment tool. Genome Res. 2002;12 4:656-64. doi:10.1101/gr.229202.

33. Bairoch A and Apweiler R. The SWISS-PROT protein sequence database and its supplement TrEMBL in 2000. Nucleic Acids Research. 2000;28 1:45-8.

34. Bateman A, Birney E, Cerruti L, Durbin R, Etwiller L, Eddy SR, et al. The Pfam Protein Families Database. Nucleic Acids Research. 2002;30 1:276-80.

35. Quevillon E, Silventoinen V, Pillai S, Harte N, Mulder N, Apweiler R, et al. InterProScan: protein domains identifier. Nucleic Acids Research. 2005;33 Web Server issue:W116-W20. doi:10.1093/nar/gki442.

36. Kanehisa M and Goto S. KEGG: Kyoto Encyclopedia of Genes and Genomes. Nucleic Acids Research. 2000;28 1:27-30.

37. The Gene Ontology C, Ashburner M, Ball CA, Blake JA, Botstein D, Butler H, et al. Gene Ontology: tool for the unification of biology. Nature Genetics. 2000;25 1:25-9. doi:10.1038/75556.

38. Sollars ESA, Harper AL, Kelly LJ, Sambles CM, Ramirez-Gonzalez RH,

Swarbreck D, et al. Genome sequence and genetic diversity of European ash trees. Nature. 2016;541:212. doi:10.1038/nature20786.

39. Unver T, Wu Z, Sterck L, Turktas M, Lohaus R, Li Z, et al. Genome of wild olive and the evolution of oil biosynthesis. Proceedings of the National Academy of Sciences. 2017;114 44:E9413-E22. doi:10.1073/pnas.1708621114.

40. Hellsten U, Wright KM, Jenkins J, Shu S, Yuan Y, Wessler SR, et al. Fine-scale variation in meiotic recombination in *Mimulus* inferred from population shotgun sequencing. Proceedings of the National Academy of Sciences. 2013;110 48:19478-82. doi:10.1073/pnas.1319032110.

41. Lan T, Renner T, Ibarra-Laclette E, Farr KM, Chang T-H, Cervantes-Pérez SA, et al. Long-read sequencing uncovers the adaptive topography of a carnivorous plant genome. Proceedings of the National Academy of Sciences. 2017;114 22:E4435-E41. doi:10.1073/pnas.1702072114.

42. Wang L, Yu S, Tong C, Zhao Y, Liu Y, Song C, et al. Genome sequencing of the high oil crop sesame provides insight into oil biosynthesis. Genome Biology. 2014;15 2:R39. doi:10.1186/gb-2014-15-2-r39.

43. Denoeud F, Carretero-Paulet L, Dereeper A, Droc G, Guyot R, Pietrella M, et al. The coffee genome provides insight into the convergent evolution of caffeine biosynthesis. Science. 2014;345 6201:1181-4. doi:10.1126/science.1255274.

44. Consortium TTG. The tomato genome sequence provides insights into fleshy fruit evolution. Nature. 2012;485:635. doi:10.1038/nature11119.

45. Iorizzo M, Ellison S, Senalik D, Zeng P, Satapoomin P, Huang J, et al. A high-quality carrot genome assembly provides new insights into carotenoid accumulation and asterid genome evolution. Nature Genetics. 2016;48:657. doi:10.1038/ng.3565.

46. The French–Italian Public Consortium for Grapevine Genome C. The grapevine genome sequence suggests ancestral hexaploidization in major angiosperm phyla. Nature. 2007;449:463. doi:10.1038/nature06148.

47. Cheng C-Y, Krishnakumar V, Chan AP, Thibaud-Nissen F, Schobel S and Town CD. Araport11: a complete reannotation of the Arabidopsis thaliana reference genome. The Plant Journal. 2017;89 4:789-804. doi:10.1111/tpj.13415.

48. Tuskan GA, DiFazio S, Jansson S, Bohlmann J, Grigoriev I, Hellsten U, et al. The Genome of Black Cottonwood, *Populus trichocarpa* (Torr. & Gray). Science. 2006;313 5793:1596-604. doi:10.1126/science.1128691.

49. Ouyang S, Zhu W, Hamilton J, Lin H, Campbell M, Childs K, et al. The TIGR Rice Genome Annotation Resource: improvements and new features. Nucleic Acids Research. 2007;35 suppl_1:D883-D7. doi:10.1093/nar/gkl976.

50. Dohm JC, Minoche AE, Holtgräwe D, Capella-Gutiérrez S, Zakrzewski F, Tafer H, et al. The genome of the recently domesticated crop plant sugar beet (*Beta vulgaris*). Nature. 2013;505:546. doi:10.1038/nature12817.

51. Li L, Stoeckert CJ and Roos DS. OrthoMCL: Identification of Ortholog Groups for Eukaryotic Genomes. Genome Research. 2003;13 9:2178-89.

doi:10.1101/gr.1224503.

52. De Bie T, Cristianini N, Demuth JP and Hahn MW. CAFE: a computational tool for the study of gene family evolution. Bioinformatics. 2006;22 10:1269-71. doi:10.1093/bioinformatics/btl097.

53. Edgar RC. MUSCLE: multiple sequence alignment with high accuracy and high throughput. Nucleic Acids Research. 2004;32 5:1792-7. doi:10.1093/nar/gkh340.

54. Guindon S, Dufayard J-F, Lefort V, Anisimova M, Hordijk W and Gascuel O. New Algorithms and Methods to Estimate Maximum-Likelihood Phylogenies: Assessing the Performance of PhyML 3.0. Systematic Biology. 2010;59 3:307-21. doi:10.1093/sysbio/syq010.

55. Sanderson MJ. r8s: inferring absolute rates of molecular evolution and divergence times in the absence of a molecular clock. Bioinformatics. 2003;19 2:301-2.

56. Doyle JA. Molecular and Fossil Evidence on the Origin of Angiosperms. Annual Review of Earth and Planetary Sciences. 2012;40 1:301-26. doi:10.1146/annurev-earth-042711-105313.

57. Wang H, Moore MJ, Soltis PS, Bell CD, Brockington SF, Alexandre R, et al. Rosid radiation and the rapid rise of angiosperm-dominated forests. Proceedings of the National Academy of Sciences. 2009;106 10:3853-8. doi:10.1073/pnas.0813376106.

Supplemental Figures

Figure S1. Length distribution of PacBio subreads.

Figure S2. K-mer frequency distribution at k-mer size of 17. A k-mer refers to an artificial sequence division of K nucleotides. From k-mer frequency, genomic characteristics (genome size, repeat structure and heterozygous rate) could be estimated. Peaks at depths of 31 and 62 were annotated with dash lines.

Figure S3. Distribution of AED scores from gene prediction. AED, Annotation Edit Distance, AED = 0 indicates perfect agreement between annotation and the evidence; AED = 1 indicates no evidence support for annotation.

Figure S4. Length distribution of annotated genes, exons and introns. **a-c** for annotated genes, exons and introns from different genome assemblies.

Supplemental Tables

Table S1. Summary of Pacbio and Illumina sequencing data generated in the present study. IDs of the study, sample, library and accession in NCBI SRA and sequencing platform, material origins of the sequenced DNA or RNA, the statistics of the raw and cleaned data are shown.

Table S2. Estimation of genome characteristics based on 17-mer statistics.

Table S3. Statistics of the different versions of the genome assembly of the scarlet sage. NA: data not available; * statistics for contigs/scaffolds.

Table S4. Summary of the annotated interspersed repeats in the genome assembly of

the scarlet sage.

Table S5. Summary of the transcriptome assemblies.

Table S6. Summary of the annotated genes. AED: Annotation Edit Distance; gene regions (including UTRs, exons and introns); genes (including 5', 3' UTRs, exons and introns).

Table S7. Summary of BUSCO evaluation of gene prediction.

Table S8. Summary of functional annotation of predicted genes.

Table S9. Genomic data used for gene families analyses. Origins, download links, assembly versions, genome properties and references of 15 analyzed genomes are shown.

Table S10. Summary of gene family analyses. Unique groups and genes, single-copy and duplicated groups and genes are summarized for the 16 analyzed genomes of 15 plant species.

Table S11. GO enrichment of expanded gene families. (A) 'Category' is the Gene Ontology (GO) term ID; (B) 'p_value' is the over represented p-value indicating the observed frequency of a given term among analyzed genes is equal to the expected frequency based on the null distribution; i.e. lower p-values indicate stronger evidence for overrepresentation; (C) 'q_value' is the Benjamini and Hochberg adjusted p-value, (D) 'numEPInCat' is the number of expanded gene families in the corresponding GO category; (E) 'numInCat' is the number of detected gene families in the corresponding GO category; (F) 'Term' is the GO term; (G) 'Ontology' indicates which ontology the

term comes from. 60 significant (q<0.05) GO-terms of three different functional categories are indicated in bold.

Table S12. KEGG enrichment of expanded gene families. (A) ‘KO category’ is the KEGG Orthology (KO) category ID; (B) ‘p_value’ is the over represented p-value indicating the observed frequency of a given term among analyzed genes is equal to the expected frequency based on the null distribution; i.e. lower p-values indicate stronger evidence for overrepresentation; (C) ‘q_value’ is the Benjamini and Hochberg adjusted p-value, (D) ‘numEPInCat’ is the number of expanded gene families in the corresponding KO category; (E) ‘numInCat’ is the number of detected gene families in the corresponding KO category; (F) ‘Pathway’ is the KEGG pathway; (G) ‘Class’ indicates which KEGG class the pathway comes from. One significant (q<0.05) KEGG pathway is indicated in bold.

Tables

Table 1. Statistics of the final genome assembly of the scarlet sage.

	Contig		Scaffold	
	**Size (bp)**	**Number**	**Size (bp)**	**Number**
**Total Size**	807,514,799	-	809,159,598	-
**Total Number**	-	2,204	-	1,525
**N10**	6,529,455	10	8,157,631	9
**N50**	2,267,074	100	3,123,266	73
**N90**	265,262	456	433,303	324
**Max.**	10,812,588	-	12,944,193	-
**Min.**	500	-	9,495	-
**Mean**	366,386	-	530,596	-
**Median**	38,049	-	48,557	-
**Gap**	-	-	1,644,799 (0.2%)	679
**GC Content**	38.84%	-	38.76%	-

Figures:

Fig. 1 Images of the scarlet sage, *Salvia splendens.*

a-b, flowers of the sequenced cultivar of *S. splendens*, "Aoyunshenghuo (Olympic flame)"; **c**, the scarlet sage with different flower colors in bedding; **d-k**, the scarlet sage with flowers of different pure colors or bi-colors.

Fig. 2 Quality of scarlet sage genome assembly and the phylogenomic inferences. Quality was assessed by comparing the scarlet genome with the recently released genomes of related species. Length distribution of contigs (**a**) and scaffolds (**b**); **c**, phylogenetic tree, divergence time, and profiles of gene families that underwent expansion or contraction. *Salvia miltiorrhiza* Zhang [8] and *Salvia miltiorrhiza* Xu [9] are two genome assemblies reported for *Salvia miltiorrhiza.*

Fig. 1

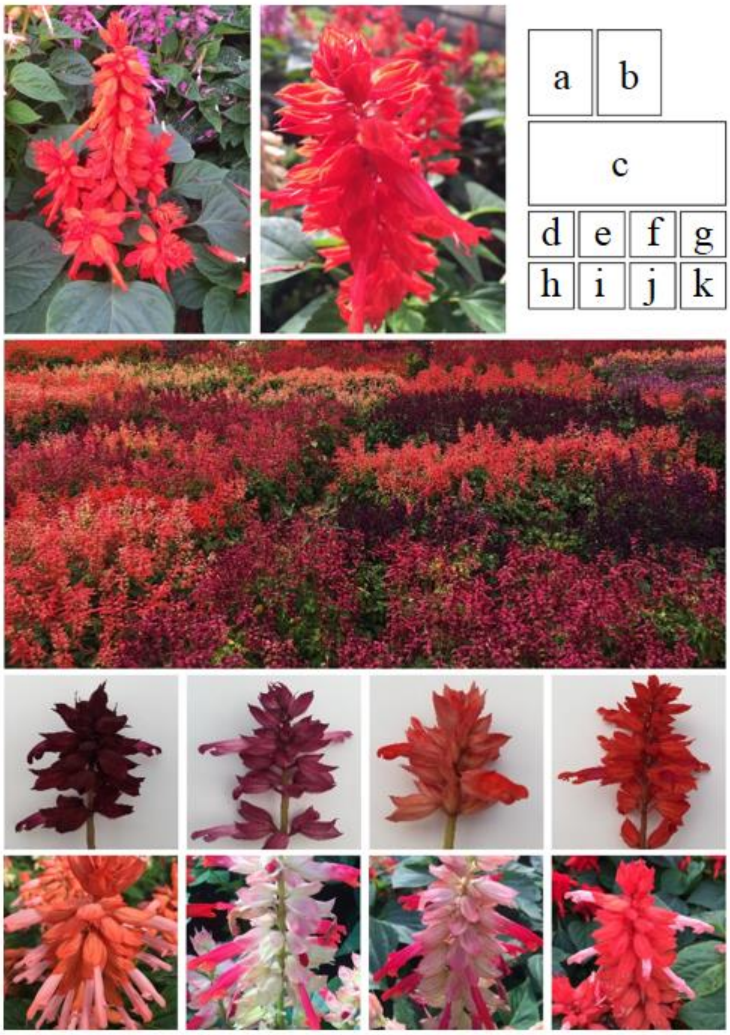

Fig. 2

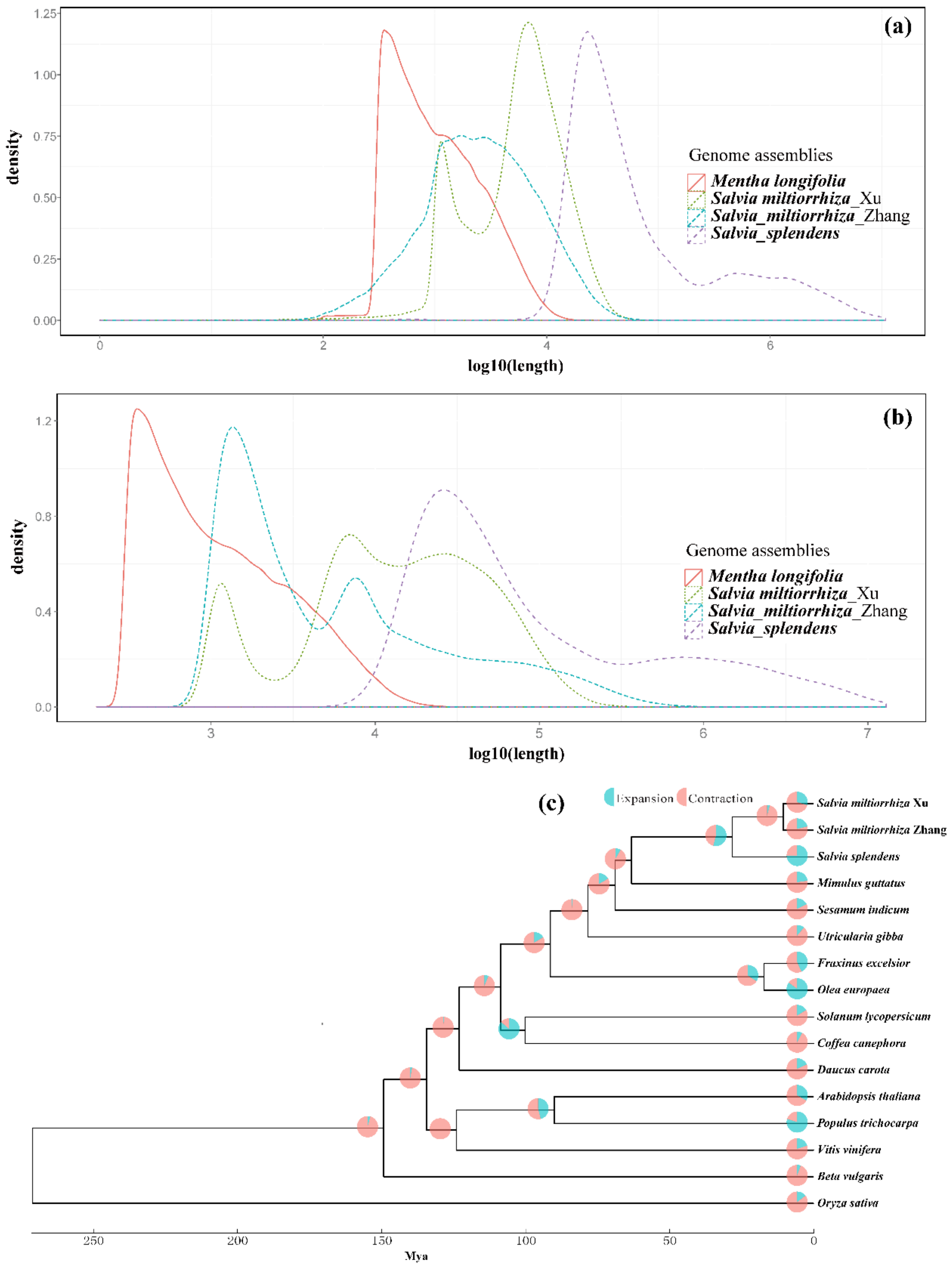
(a)
density
log10(length)
Genome assemblies
Mentha longifolia
Salvia miltiorrhiza_Xu
Salvia_miltiorrhiza_Zhang
Salvia_splendens
(b)
density
log10(length)
Genome assemblies
Mentha longifolia
Salvia miltiorrhiza_Xu
Salvia_miltiorrhiza_Zhang
Salvia_splendens
(c)
Expansion
Contraction
Salvia miltiorrhiza Xu
Salvia miltiorrhiza Zhang
Salvia splendens
Mimulus guttatus
Sesamum indicum
Utricularia gibba
Fraxinus excelsior
Olea europaea
Solanum lycopersicum
Coffea canephora
Daucus carota
Arabidopsis thaliana
Populus trichocarpa
Vitis vinifera
Beta vulgaris
Oryza sativa
Mya

Click here to access/download
Supplementary Material
Table_S1.xlsx

Click here to access/download
Supplementary Material
Table_S2.docx

Click here to access/download
Supplementary Material
Table_S3.docx

Click here to access/download
Supplementary Material
Table_S4.docx

Click here to access/download
Supplementary Material
Table_S5.docx

Click here to access/download
Supplementary Material
Table_S6.docx

Click here to access/download
Supplementary Material
Table_S7.docx

Click here to access/download
Supplementary Material
Table_S8.docx

Click here to access/download
Supplementary Material
Table_S9.docx

Click here to access/download
Supplementary Material
Table_S10.docx

Click here to access/download
Supplementary Material
Table_S11.xlsx

Click here to access/download
Supplementary Material
Table_S12.docx

Click here to access/download
Supplementary Material
Fig_S1.pdf

Click here to access/download
Supplementary Material
Fig_S2.pdf

Click here to access/download
Supplementary Material
Fig_S3.pdf

Click here to access/download
Supplementary Material
Fig_S4.pdf

Personal Cover Click here to download Personal Cover Salvia_genome_cover_letter.docx

January 26, 2018

Dr. Laurie Goodman, Editor of GigaScience

RE: “**High quality assembly of the reference genome for scarlet sage, *Salvia splendens*, an economically important ornamental plant**”

Dear Dr. Goodman,

Attached, please find the above-mentioned manuscript that my colleagues and I are submitting to **GigaScience** for possible publication as a **Data Note**.

The scarlet or tropical sage (*Salvia splendens*) is a tender herbaceous perennial widely introduced all over the world for ornamental purposes thus representing considerable economic value. Currently, few molecular resources exist for this species, and thus, improvement is still restricted to traditional phenotypic selection. In order to further improve selection for advantageous traits, the genetic mechanisms underlying phenotypic variation need to be further explored. Here, we provide a comprehensive new resource for *Salvia* genomics research based on: (1) PacBio Single-Molecule Real-Time (SMRT) and Illumina short-read sequencing providing long- and short-reads respectively, useful for *Salvia* high quality genome assembly, (2) genes and whole genome annotations with comprehensive bioinformatics computation (genetic diversity, DNA repeats annotations) and the help of a large set of RNA sequences obtained from multiple tissues, (3) gene family evolution characterisations (expansion; contraction) and phylogenomic analyses (estimated times of divergence) using a representative set of 14 additional angiosperm species. The availability of these resources will prove to be of great importance for further breeding strategies of *Salvia*, genome editing and also for comparative genomics among related species.

We attest that this manuscript has not been submitted to any other journal for publication. We also confirm that all the listed coauthors contributed to the study, and have read and approved the manuscript and are free from any conflicts of interest.

We look forward to hearing from you.

Sincerely Yours,
Jian-Feng Mao

Beijing Forestry University,
Qinghua East Road No 35.
Beijing, 100083,
P. R. China.
