# Supplementary material for: High-quality assembly of the reference genome for scarlet sage, Salvia splendens, an economically important ornamental plant
Source: Gigascience. 2018 Jun 19;7(7):giy068. doi: 10.1093/gigascience/giy068 (PMC6030905; doi:10.1093/gigascience/giy068)
Supplement: GIGA-D-18-00028_Revision_1..pdf [file giy068_giga-d-18-00028_revision_1..pdf]

GigaScience

High quality assembly of the reference genome for scarlet sage, Salvia splendens, an economically important ornamental plant

--Manuscript Draft--

**Manuscript Number:**	GIGA-D-18-00028R1
**Full Title:**	High quality assembly of the reference genome for scarlet sage, Salvia splendens, an economically important ornamental plant
**Article Type:**	Data Note
**Funding Information:**	Beijing Key Laboratory of Greening Plants Breeding (Z201605) — Dr. Hai-Bo Xin<br>Fundamental Research Funds for the Central Universities (YX2013-41) — Mr. Jian-Feng Mao
**Abstract:**	Background: Salvia splendens Ker-Gawler, scarlet or tropical sage, is a tender herbaceous perennial widely introduced and seen in public gardens all over the world. With few molecular resources, breeding is still restricted to traditional phenotypic selection, and the genetic mechanisms underlying phenotypic variation still remain unknown. Hence, a high quality reference genome will be very valuable for marker assisted breeding, genome editing or molecular genetics.<br>Findings: We generated 66 gigabases (Gb) and 37 Gb of raw DNA sequences, respectively, from whole-genome sequencing of a largely homozygous scarlet sage inbred line using PacBio Single-Molecule Real-Time (SMRT) and Illumina HiSeq sequencing platforms. PacBio de novo assembly yielded a final genome with a scaffold N50 size of 3.12 megabases (Mb), and a total length of 808 Mb. The repetitive sequences identified accounted for 57.52% of the genome sequence and 54,008 protein-coding genes were predicted collectively with ab initio and homology-based gene prediction from the masked genome. The divergence time between S. splendens and S. miltiorrhiza was estimated with 28.21 million years ago (Mya). Moreover, 3,797 species-specific genes and 1,187 expanded gene families were identified for the scarlet sage genome.<br>Conclusions: We provide the first genome sequence and gene annotation for the scarlet sage. The availability of these resources will be of great importance for further breeding strategies, genome editing and also for comparative genomics among related species.
**Corresponding Author:**	Ri-Chen Cong, Ph.D<br>Beijing Institute of Landscape Architecture<br>Beijing, CHINA
**Corresponding Author Secondary Information:**	
**Corresponding Author's Institution:**	Beijing Institute of Landscape Architecture
**Corresponding Author's Secondary Institution:**	
**First Author:**	Ai-Xiang Dong
**First Author Secondary Information:**	
**Order of Authors:**	Ai-Xiang Dong
	Hai-Bo Xin
	Zi-Jing Li
	Hui Liu
	Yan-Qiang Sun
	Shuai Nie

Powered by Editorial Manager® and ProduXion Manager® from Aries Systems Corporation

	Zheng-Nan Zhao
	Rong-Feng Cui
	Ren-Gang Zhang
	Quan-Zheng Yun
	Xin-Ning Wang
	Fatemeh Maghuly
	Ilga Porth
	Ri-Chen Cong
	Jian-Feng Mao, Ph.D.
**Order of Authors Secondary Information:**	
**Response to Reviewers:**	We provide our comments directly underneath the points raised by you and within the three reviewers' reports as follows:<br><br>AE: Please pay particular attention to reviewer #2's comment number 3: "Since the genomes of Salvia miltiorrhiza (Zhang et al. and Xu et al.) and Mentha longifolia have been published, a more detailed analysis about differences between Salvia splendens and the other two plants should be conducted, so as to highlight the importance of Salvia splendens. "<br>R: We provided synteny analyses among detected metabolic gene cluster between the Salvia genomes. One section of comparative genomics was added (also see Figure S7 for synteny blocks). However, even mentha genome has been published, its gene annotation data are not publicly available. We wrote two emails to the corresponding authors for two times, we did not get any response. So mentha genome was not included in out comparative genomic studies.<br><br>AE: Your manuscript is under consideration as a Data Note, and although we do not require in-depth exploration of biological questions for this article type, I fully agree with the referee that it is crucially important that you provide some detailed context regarding the other published Salvia and Mentha genomes - what are similarities and differences, and what are unique features of Salvia splendens.<br>R: please see answer provided above.<br><br>AE: Please also clarify a number of technical issues mentioned by reviewer 3, e.g. regarding your scaffolding approach, as well as the use of Pilon and BUSCO.<br>R: Please see answers to Reviewer 3.<br><br>AE: As an editorial point, I notice that you indicate 4 "equally contributing" first authors. Please note that we allow a maximum of 3 co-first authors (and only if their contributions are really absolutely equal). Please revise the author role indications accordingly.<br>R: Revised. Now we have 3 co-first authors.<br><br>Reviewer #1: The authors of "High quality assembly of the reference genome for scarlet sage, Salvia splendens, an economically important ornamental plant" describe their efforts in generating a reference sequence for the plant Salvia splendens that is spread out in multiple gardens. Overall the authors relied mainly on Pacbio to obtain a high quality reference genome sequence using state of the art methods. Furthermore, they annotated the genome using RNA-Seq reads and state of the art methods such as maker, Augustus etc. Thus, I don't have any comments or concerns.<br>R: Thank you.<br><br>Reviewer #2: This manuscript described the construction of genome sequence and annotation for Salvia splendens Ker-Gawler. A hybrid approach using PacBio Single-Molecule Real-Time (SMRT) and Illumina HiSeq sequencing platforms was employed. Finally, a genome of 808Mb and 54,008 protein-coding genes were reported. The genome should be pretty completed because 1) the genome size is already bigger than the k-mer estimated genome size; 2) supported by BUSCO results and 3) satisfactory N50 and contig / scaffold number. However, this is not the first species of

the same genus and more functional information should be included to improve the novelty and usefulness of this piece of work. Otherwise, this will be only another genome sequence deposited in the database.
R: Thank you. Regarding more functional information provision from genomic data, please see our comments immediately below.

Reviewer #2: Comments and suggestions:
2.1. As mentioned in the introduction, many species of this genus are extensively used for culinary purposes, essential oil production and Chinese herbal remedies. Therefore, it is expected that the active ingredients of the plant responsible for its biological and therapeutic functions should be quite well known. If the metabolic pathways responsible for the production of these ingredients could be dissected, the information reported could be more useful for researchers working on this plant species.
R: One section (lines 284-332) involving description and analysis of metabolic pathways, gene clusters and comparative genomics was added. Two pathways of flavonoid and menthol biosynthesis were constructed by homolog mapping with the help of the Plant Metabolic Network (PMN v12.5, https://www.plantcyc.org/). Results were summarized in Figure S5 and S6, Supplementary_File_1.

2.2. Regarding the transcriptome analysis, results had been generated using tissues obtained from roots, shoots, leaves, calyxes and corollas. For gene discovery, mixing all the datasets to generate the transcript set is reasonable. However, to highlight the therapeutic value of particular part(s) of the plant, differential expression analysis and gene clustering would be expected.
R: Yes, this true. Our immediate intention was to identify the overall metabolic gene clusters for the two Salvia genomes, and related gene co-expression profiles were further examined among the co-localized genes. These gene clusters were summarized in Table S13, and genomic composition of gene clusters and gene expression were detailed in Supplementary File 2 and 3 (lines 284-321). A follow-up study could now target more specifically the genes of interest that promise to be correlated with variation in the therapeutic value of certain compounds and in the different plant parts and confidently identify those with the highest value.

2.3. Since the genomes of Salvia miltiorrhiza (Zhang et al. and Xu et al.) and Mentha longifolia have been published, a more detailed analysis about differences between Salvia splendens and the other two plants should be conducted, so as to highlight the importance of Salvia splendens. Moreover, the functional significance of such differences should be extensively explored and discussed. Finally, certain experiments should be done if necessary.
R: We provided synteny analyses among the detected metabolic gene cluster between the Salvia genomes. One section (lines 284-332) of comparative genomics was added to our manuscript (also see Figure S7). However, even though the mentha genome has been published, curiously, its gene annotation data is not publicly available! We wrote two emails to the corresponding authors, but we did not get any response. Thus, at this time, unfortunately, the mentha genome could not be included in our comparative genomic studies.

Reviewer #3: Dong et al. provide a near complete reference genome for the ornamental crop Salvia splendens using a PacBio sequencing approach. The assembly is high quality and will be useful for the plant comparative genomics community. The approaches are technically sound and adequate details on the assembly and annotation of this genome are provided. I have a few minor concerns I feel should be addressed before this manuscript is published.
R: Thanks.

Reviewer #3: Comments and suggestions:
3.1 The assembly metrics of the Salvia genome are exceptionally good and the near completeness of this assembly will make it useful for the comparative genomics community. The scaffolding is potentially problematic given the short read lengths of the Illumina data and the lack of an additional set of PacBio data that was not utilized in the initial assembly. The authors used 4-5 different scaffolding algorithms on the same datasets, potentially introducing errors. Most of these scaffolding and gap filling programs were designed to utilize mate pair data to bridge repeats and not the short insert libraries produced by the authors. The Illumina data could falsely bridge gaps

Powered by Editorial Manager® and ProduXion Manager® from Aries Systems Corporation

	creating chimeric, misassembled scaffolds.<br>R: Indeed, we used two sets of PacBio reads from two individual plants, and just one set of Illumina reads. Genome assembly was processed in two main steps in this study as follows: We firstly generated the primary assemblies with different algorithms based on one set of PacBio reads. Then, the other set of PacBio reads was utilized in a further scaffolding step starting from the best assembly from the primary step. We provided a detailed description for genome assembly in this revision now to avoid ambiguity in the method description. We were trying to explore extra information from the Illumina short reads in the second scaffolding step, while taking care of the potential false bridge. In fact, Illumina did provide us only few values.<br><br>3.3 Line 162. The aligner used to map the Illumina reads to the Salvia genome for Pilon based polishing should be provided. Parameters for Pilon and the number of corrected indels/SNPs should also be listed.<br>R: Yes, we did it. Pl. see lines 164-170.<br><br>3.4 Line 216 and Line 225: It is unclear why two different BUSCO datasets were used to verify the completeness of the genome assembly/annotation.<br>R: We assured that only one BUSCO dataset (1,440 single copy orthologs of the Viridiplantae database) was used in this study. We wrongly input the description for BUSCO dataset. Now we corrected it throughout the text.<br><br>3.5 It would be interesting to include more downstream comparative genomics analyses for this species, but I suspect this is beyond the scope of this manuscript.<br>R: We did further provide functional analyses according to the second reviewer. However, no real comparative genomic analyses were provided as published genomes of Salvia miltiorrhiza (Zhang et al. and Xu et al.) and Mentha longifolia are really low quality or no protein annotation has yet been released which prevented further comparative study.
**Additional Information:**	
**Question**	**Response**
Are you submitting this manuscript to a special series or article collection?	No
**Experimental design and statistics**<br><br>Full details of the experimental design and statistical methods used should be given in the Methods section, as detailed in our Minimum Standards Reporting Checklist. Information essential to interpreting the data presented should be made available in the figure legends.<br><br>Have you included all the information requested in your manuscript?	Yes
**Resources**<br><br>A description of all resources used, including antibodies, cell lines, animals and software tools, with enough information to allow them to be uniquely identified, should be included in the Methods section. Authors are strongly encouraged to cite Research Resource Identifiers (RRIDs) for antibodies, model organisms and tools, where possible.	Yes

Have you included the information requested as detailed in our Minimum Standards Reporting Checklist?	
**Availability of data and materials**<br><br>All datasets and code on which the conclusions of the paper rely must be either included in your submission or deposited in publicly available repositories (where available and ethically appropriate), referencing such data using a unique identifier in the references and in the “Availability of Data and Materials” section of your manuscript.<br><br>Have you have met the above requirement as detailed in our Minimum Standards Reporting Checklist?	Yes

Manuscript

Click here to download Manuscript Salvia_Genome_paper-revision_SCEedits_track.docx

High quality assembly of the reference genome for scarlet sage, *Salvia splendens*, an economically important ornamental plant

Ai-Xiang Dong[1‡], Hai-Bo Xin[1,2‡], Zi-Jing Li[1‡], Hui Liu[2], Yan-Qiang Sun[2], Shuai Nie[2], Zheng-Nan Zhao[1], Rong-Feng Cui[1], Ren-Gang Zhang[3], Quan-Zheng Yun[3], Xin-Ning Wang[3], Fatemeh Maghuly[4], Ilga Porth[5], Ri-Chen Cong[1*], Jian-Feng Mao[2*]

[1] Beijing Key Laboratory of Greening Plants Breeding, Beijing Institute of Landscape Architecture, Beijing, 10020, China.

[2] Beijing Advanced Innovation Center for Tree Breeding by Molecular Design, National Engineering Laboratory for Tree Breeding, Key Laboratory of Genetics and Breeding in Forest Trees and Ornamental Plants, Ministry of Education, College of Biological Sciences and Technology, Beijing Forestry University, Beijing, 100083, China.

[3] Beijing Ori-Gene Science and Technology Co. Ltd, Beijing, 10226, China.

[4] Plant Biotechnology Unit (PBU), Dept. Biotechnology, BOKU-VIBT, University of Natural Resources and Life Sciences, Muthgasse 18, 1190 Vienna, Austria. ORCID: 0000-0001-5433-0070.

[5] Département des sciences du bois et de la forêt, Pavillon Charles-Eugène-Marchand, 1030, Avenue de la Médecine, Université Laval, Québec (Québec) G1V 0A6, Canada. ORCID: 0000-0002-9344-6348

‡These authors contributed equally to this paper.

*Correspondence to: hardhopeee@163.com (RCC), ORCID: 0000-0002-4619-6120; jianfeng.mao@bjfu.edu.cn (JFM), ORCID: 0000-0001-9735-8516

Abstract

Background: *Salvia splendens* Ker-Gawler, scarlet or tropical sage, is a tender herbaceous perennial widely introduced and seen in public gardens all over the world. With few molecular resources, breeding is still restricted to traditional phenotypic selection, and the genetic mechanisms underlying phenotypic variation still remain unknown. Hence, a high quality reference genome will be very valuable for marker assisted breeding, genome editing or molecular genetics.

Findings: We generated 66 gigabases (Gb) and 37 Gb of raw DNA sequences, respectively, from whole-genome sequencing of a largely homozygous scarlet sage inbred line using PacBio Single-Molecule Real-Time (SMRT) and Illumina HiSeq sequencing platforms. PacBio *de novo* assembly yielded a final genome with a scaffold N50 size of 3.12 megabases (Mb), and a total length of 808 Mb. The repetitive sequences identified accounted for 57.52% of the genome sequence and 54,008 protein-coding genes were predicted collectively with *ab initio* and homology-based gene prediction from the masked genome. The divergence time between *S. splendens* and *S. miltiorrhiza* was estimated with 28.21 million years ago (Mya). Moreover, 3,797 species-specific genes and 1,187 expanded gene families were identified for the scarlet sage genome.

Conclusions: We provide the first genome sequence and gene annotation for the scarlet sage. The availability of these resources will be of great importance for further breeding strategies, genome editing and also for comparative genomics among related species.

Keywords: annotation, evolution, reference genome, *Salvia splendens,* scarlet sage

Data description

Background information

Salvia L., with nearly 1,000 species of shrubs, herbaceous perennials, and annuals, is the largest genus in the mint family (Lamiaceae: Nepetoideae: Mentheae: Salviinae) [1-4]. The genus is widely distributed throughout the world. Many species of this genus are extensively used for culinary purposes, essential oil production and Chinese herbal remedies such as the two species *S. officinalis* [3] and *S. miltiorrhiza* (Danshen). Additionally, they are used as ornamental plants valued for their flowers or for their aromatic foliage such as *S. splendens* (**Fig. 1 a-k**).

S. splendens (NCBI taxon ID:180675), scarlet or tropical sage, is a herbaceous perennial species, which is native to Brazil. While it is a perennial in warmer climate zones, it grows as an annual in cooler areas. *S. splendens* is a very popular bedding plant, and is widely cultivated in public gardens all over the world [3, 5], characterized by its dense flowers, and wide variation of colours (scarlet, purple, pink, blue, lavender, salmon, yellow green, white and bicolor), as well as long lasting flowering (3-9 weeks or even longer). Additionally, *S. splendens* can provide

outstanding visual effects when grown in beds, borders and containers with long-lasting lifespans ranging from late spring to the occurrence first frost. Furthermore, the flower is easy to maintain and fairly free of pests and diseases due to Lamiaceae's characteristic insect repellent fragrance content [6]. The plant blends nicely with other annuals or perennial plants for the best visual effects in an ensemble setting; in addition this plant requires little deadheading as well it attracts various butterfly species. *S. splendens* is a prolific and durable bloomer, thrives in full sun, and survives in a large range of soil moisture regimes.

Traditional breeding activities using phenotypic selection as well as performing targeted variety hybridizations between elite cultivars have resulted in a large number of new cultivars with different performances regarding flowering characters (related to colour, flowering time, flowering period amongst others), individual growth performance, height, and/or tolerance to moisture or temperature extremes. However, little is known about the molecular mechanisms underlying such economically important characteristics for ornamental varieties. To date, only few genetic markers [7] are available for marker assistant breeding or genetic modification.

In the current study, we present the first high quality genome assembly for *S. splendens* with a hybrid assembly strategy using PacBio Single-Molecule Real-Time and Illumina HiSeq short-read sequencing platforms. The genome assembly, its structural and functional annotation, provide a valuable reference for the genomic dissection of the phenotypic variation in *Salvia*, and new breeding strategies. This

reference genome could also be used in comparative genomics with the recently released *Salvia* genome (*S. miltiorrhiza*) [8, 9] and the mint genome (*Mentha longifolia*) [10] to study the biosynthesis of important fragrant and medicinal compounds.

Plant material

We chose the elite variety *S. splendens*, "Aoyunshenghuo (Olympic flame)" (**Fig. 1 a-b**) for whole genome sequencing, which was originally developed by multiple rounds of selection/selfing of one hybrid to obtain this inbred line. This cultivar is characterized by resistance to drought, high temperature, and improved performance related to a longer flowering period; it is well adapted to climate conditions across North China, and therefore grows well in Beijing. Because of the high homozygosity obtained due to advanced generation selfing, this cultivar shows no phenotypic segregation, a characteristic of important commercial value. Seeds of this cultivar were provided by the Beijing Institute of Landscape Architecture germplasm bank.

PacBio SMRT sequencing

High quality high molecular weight genomic DNA was extracted from leaves of two soil-grown seedlings (huo1 and huo1_1) following ~20 kb SMRTbell™ Libraries" protocol (http://www.pacb.com/wp-content/uploads/2015/09/Shared-Protocol-Preparing-Arabi

dopsis-DNA-for-20-kb-SMRTbell-Libraries.pdf). Plants for DNA extraction have been placed in the dark for 48 h before harvesting the leaf material. DNA was purified with Mobio PowerClean® Pro DNA Clean-Up Kit and quality was assessed by standard agarose gel electrophoresis and Thermo Fisher Scientific Qubit Fluorometry. Genomic DNA was sheared to a size range of 15–40 kb using either AMPure beads (Beckman Coulte) or g-TUBE (Covaris), and enzymatically repaired and converted into SMRTbell template libraries as recommended by Pacific Biosciences. Following this procedure, hairpin adapters were ligated following exonucleasese-based digestion (of the remaining damaged DNA fragments and those fragments without adapters at both ends). Subsequently, the resulting SMRTbell templates were size-selected by Blue Pippin electrophoresis (Sage Sciences) and templates ranging from 15 to 50 kb were sequenced on a PacBio RS II instrument using P6-C4 sequencing chemistry (25 Single-Molecule Real-Time (SMRT) cells for individual huo1) and on a PacBio Sequel instrument using S/P2-C2 sequencing chemistry (8 SMRT cells for the other individual, huo1_1). A total of 8,858,116 PacBio post-filtered reads were generated. This produced a total of 65,962,079,028 bp (roughly 82x of the assembled genome) of single-molecule sequencing data, with an average read length of 7,446 bp (**Fig. S1** and **Table S1)**.

Illumina short-read sequencing

DNA was extracted from leaf tissue of the same soil-grown seedlings (huo1 and

huo1_1) using the Qiagen DNeasy Plant Mini Kit. Two 500 bp paired-end (PE) libraries (huo1 and huo1_1) were prepared using the NEBNext Ultra DNA Library Prep Kit for Illumina sequencing with an Illumina HiSeq X Ten machine. Short reads were processed with Trimmomatic v0.33 (Trimmomatic, RRID:SCR_011848) [11, 12] and Cutadapt v1.13 (cutadapt, RRID:SCR_011841) [13, 14] to remove adapter sequences and leading and trailing bases with a quality score below 20 and reads with an average per-base-quality of 20 over a 4 bp sliding window. Reads < 70 nucleotides in length after trimming were removed from further analysis. A total of 265.53 million reads were generated. This produced a total of 36.83 Gb (roughly 40x of the assembled genome) of raw sequencing data, with an average cleaned read length of 137 bp (**Table S1**).

Estimation of genome size, heterozygosity, and repeat content

All generated PacBio reads were filtered and corrected with Canu v1.5 (Canu, RRID:SCR_015880) [15], thereafter, Jellyfish (Jellyfish, RRID:SCR_005491) [16] was used to count occurrence of k-mers based on the processed data. Finally, gce 1.0.0 [17] was employed to estimate the overall characteristics of the genome such as genome size, repeat contents and heterozygous rate. In this study, a total of 22,117,819,357 k-mers were generated and the peak k-mer depth was 31 (**Fig. S2**). The genome size was estimated to be approximately 711 Mb (**Table S2**) and the final cleaned data corresponded to the coverage of about 33-fold. Repeat and error rates

were estimated to be 47.99% and 0.27%, respectively, and heterozygosity rate was 0.06%.

De novo genome assembly

De novo assembly was conducted as follows in a progressive manner. Firstly, primary assemblies were generated from PacBio long reads of the 31 Gb from the 'huo1' sequenced individual by four different Overlap-Layout-Consensus (OLC) based assemblers, Canu (produced assembly v0.1), MECAT 1.1 (assembly v0.2) [18], FALCON v0.7 (Falcon, RRID:SCR_016089) [19] (https://github.com/PacificBiosciences/FALCON/) after Canu correction (v0.3) and SMARTdenovo 1.0.0 (https://github.com/ruanjue/smartdenovo) after Canu correction (v0.4) (**Table S3**). Based on the size of the assembled genome, the total number of assembled contigs, N50, the L50, maximum length of the contigs, and also the completeness of the genome assembly as assessed by using BUSCO criteria v2.0.1 (BUSCO, RRID:SCR_015008)[20] (1,440 single copy orthologs of the Viridiplantae database) with the BLAST E-value cutoff of 10^{-5}, assembly (v0.1) from Canu was chosen for further polishing and scaffolding. In this selected primary assembly, the assembled genome size was 808 Mb distributed across 2,306 contigs with N50 of 2.06 Mb, L50 of 109 and maximum contig length of 8.88 Mb. We also confirmed on average 92.1% gene completeness in this assembly (**Table S3**). In the following steps, the arrow algorithm v2.2.1

(https://github.com/PacificBiosciences/GenomicConsensus) was used to further improve the assembly based on PacBio long reads (v1.0), after which SSPACE-LongRead 1.1 [21] and SSPACE-standard 3.0 (SSPACE, RRID:SCR_005056) [22] were used for subsequent scaffold assembly based on PacBio long reads of 35 Gb from the second sequenced individual 'hou1_1' and Illumina short reads, respectively. Finally, after scaffold processing and subsequent gap filling with SOAPdenovo and GapCloser (GapCloser, RRID:SCR_015026) [23] (v1.1), arrow v2.2.1 algorithm (based on PacBio long reads) and pilon (Pilon, RRID:SCR_014731) (based on Illumina short reads, and run two times, parameters for Pilon: --changes --diploid –dumpreads.), we got the final genome assembly (v1.2). Mapping of Illumina reads was done by using Bowtie2 v2.3.0 (Bowtie, RRID:SCR_005476) [24]. We detected 400,170 SNPs, 96,854 insertions and 62,637 deletions, respectively, for the first pilon run, while, subsequently, a greatly decreased number of variants for the second pilon run (40,465 SNPs, 6,935 insertions and 9,976 deletions, respectively). In this final assembly, we gained an assembled genome size of 808 Mb characterized by 2,204 contigs and 1,525 scaffolds (with contig N50 of 2.27 Mb and scaffold N50 of 3.12 Mb), and by gene completeness of 92.2% (**Table 1 and Table S3**). This assembly represents the highest continuity and completeness among the recently released genome assemblies for the *Salvia* genus [8, 9] and the mint [10], as it was examined by length distribution plotting of contigs and scaffolds as shown in **Fig. 2a, b**.

DNA repeats annotation

RepeatModeler v1.0.10 (RepeatModeler, RRID:SCR_015027) (http://www.repeatmasker.org/RepeatModeler.html) was employed to *de novo* identify and classify repeat families in the genome assembly. Subsequently, the outputs from RepeatModeler and RepBase [25] library were combined and used as repeat library for subsequent RepeatMasker (RepeatMasker, RRID:SCR_012954) (v4.0.7, rmblast-2.2.28) (http://www.repeatmasker.org/) analyses, which was used to fully discover and identify repeats within the assembled genome. In summary, 57.52% of the genome was annotated as repeats among which we found 1.08% simple repeats and 40.35% known transposable elements (TE). Long terminal repeats (LTRs) constituted the greatest proportion, 26.49% of the genome, and DNA TE made up 11.91% of the genome. Gypsy (18.15% of the genome) and Copia (7.92%) TEs were the largest components of LTRs. The results of repeat annotations are summarized in **Table S4**.

RNA sequencing, transcriptome assembly and functional annotation

RNA was extracted from the two cultivated lines with different flower colours (red and purple) using tissue obtained from, roots, shoots, leaves, calyxes and corollas. Frozen tissue from all samples was ground manually using mortar and pestil, and RNA was isolated using the NEBNext Poly(A) mRNA Magnetic Isolation Module.

RNA quality was assessed using an Agilent 2100 BioAnalyzer. Sequencing libraries were prepared using the NEBNext Ultra RNA Library Prep Kit for Illumina. 150 bp PE sequencing was performed using an Illumina HiSeq X Ten.

1,344 million raw reads from RNA sequencing were processed by Trimmomatic and Cutadapt and aligned to the genome assembly with HiSat2 v2.1.0 (HiSat2, RRID:SCR_015530) [26]. Base quality was checked with FastQC (FastQC, RRID:SCR_014583) (https://www.bioinformatics.babraham.ac.uk/projects/fastqc/) before and after data cleaning, and respective statistics of RNA sequencing data are shown in **Table S1**. Reference genome guided transcriptome assemblies were independently prepared with Cufflinks v2.1.1 (Cufflinks, RRID:SCR_014597) [27], StringTie v1.3.3b (StringTie, RRID:SCR_016323)[28] and Trinity v2.0.6 (Trinity, RRID:SCR_013048) [29]. *De novo* assembly was generated using Trinity, then, transcriptome assemblies were combined and further refined using CD-HIT v4.6 [30], and finally, 192,169 unique transcripts were gained. The summary of the transcriptome assemblies is shown in **Table S5**.

AUGUSTUS v3.2.3 (Augustus, RRID:SCR_008417) [31] was employed for *ab initio* gene prediction, using model training based on coding sequences from *Arabidopsis thaliana* and *S. miltiorrhiza* (with two sets of proteins from independent genome annotation [8, 9]). Then, transcripts from RNA sequencing were aligned to the repeat-masked reference genome assembly with BlastN and TblastX from BLAST v2.2.28+ (NCBI BLAST, RRID:SCR_004870) [32] (E-value cutoff of 10^{-5}), and

protein sequences from *A. thaliana* and *S. miltiorrhiza* were aligned to the repeat-masked reference genome assembly with BlastX (E-value cutoff of 10^{-5}). After optimization with Exonerate v2.4.0 (https://www.ebi.ac.uk/about/vertebrate-genomics/software/exonerate) [33], gene model predictions were finalized prepared using the MAKER package v2.31.9 (MAKER, RRID:SCR_005309) [34] provided within AUGUSTUS. To assess the quality of the gene prediction, AED (Annotation Edit Distance) scores were generated for each of the predicted genes as part of the MAKER pipeline. Putative function for each identified gene was assessed by performing a BLAT (BLAT, RRID:SCR_011919) [35] search of the peptide sequences against the Uniprot database (UniProt, RRID:SCR_002380) [36], Protein annotation against PFAM (Pfam, RRID:SCR_004726) [37] and InterProScan (InterProScan, RRID:SCR_005829) [38] ID were also conducted using the scripts provided in the MAKER package. Completeness of gene annotation was checked using BUSCO (1,440 single copy orthologs of the Viridiplantae database) with a BLAST E-value cutoff of 10^{-5}.

54,008 genes could be predicted, with average lengths of gene regions, genes (including 5', 3' UTRs, exons and introns), CDS and exons of 3,430.43 bp, 1696.34 bp, 1293.62 bp and 265.94 bp, respectively (**Table S6**). The comparisons among genomes from related species regarding lengths of genes, exons, and introns are shown in **Fig. 2**. The distribution of AED tagged by MAKER is shown in **Fig. S3**, in

which about 97% of the annotated genes (52,338 genes) had an AED < 0.5 (**Table S6**), thus indicating that the annotation is well supported. The result from BUSCO assessment of the quality of the genome assembly and annotation is shown in **Table S7**. 92.08 % of the universal single-copy genes (1,326 genes out of the total 1,440 genes) were identified, supporting the high quality of the genome assembly. Among the 1,326 BUSCO conserved single-copy genes detected in the scarlet genome, 466 genes were found single-copy, while 860 genes were duplicated (**Table S7**).

The predicted genes were annotated against several functional databases, including: (1) the NCBI non-redundant protein database (Nr; http://www.ncbi.nlm.nih.gov), (2) Swiss-Prot protein database (http://www.expasy.ch/sprot) [36], (3) Translated EMBL-Bank (part of the International Nucleotide Sequence Database Collaboration, TrEMBL, http://www.ebi.ac.uk/uniprot) [36], (4) the protein families database (Pfam; http://pfam.xfam.org/), (5) Cluster of Orthologous Groups for eukaryotic complete genomes (KOG) database (http://genome.jgi-psf.org/help/kogbrowser.jsf), (6) KO (the Kyoto Encyclopedia of Genes and Genomes, Orthology) database (http://www.genome.jp/kegg/ko.html) [39], and (7) Gene ontology (GO) (http://www.geneontology.org) [40]. 94.67 % of all predicted genes could be annotated with the following protein related databases: NR (94.60 %), Swiss-Prot (63.40 %), TrEMBL (93.50 %), Pfam (82.10 %), KOG (90.05 %), KO (37.40 %), and GO (78.80 %) (**Table S8**).

Identification of orthologous genes and phylogenetic inference

To analyze gene families, we downloaded the protein sequences of 15 additional species (*Salvia miltiorrhiza* [8, 9], *Fraxinus excelsior* [41], *Olea europaea* [42], *Mimulus guttatus* [43], *Utricularia gibba* [44], *Sesamum indicum* [45], *Coffea canephora* [46], *Solanum lycopersicum* [47], *Daucus carota* [48], *Vitis vinifera* [49], *Arabidopsis thaliana* [50], *Populus trichocarpa* [51], *Oryza sativa* [52] and *Beta_vulgaris* [53]) (**Table S9**). Orthologous and paralogous gene clusters were identified among species using OrthoMCL v2.0.9 [54]. Recommended settings were used for all-against-all BLASTP comparisons (Blast+ v2.3.056) [32] and OrthoMCL[22] analyses.

A total of 35,808 OrthoMCL families were built based on effective database sizes of all versus all BLASTP with an E-value of 10^{-5} and a Markov Chain Clustering default inflation parameter. We identified 1,306 gene families (3,797 genes) that were specific to the scarlet sage genome when comparing with the other 15 genomes (**Table S10**), and we detected 10,770 gene families that have expanded in the scarlet sage lineage, using CAFE v4.0 [55, 56] (**Fig. 2c**). The expanded gene families were enriched for 60 significant ($q<0.05$) GO-terms of three different functional categories, i.e. BP, CC, and MF (**Table S11**) and one KEGG pathway (amino acid metabolism) (**Table S12**) significant at $q<0.05$. Also, 3,579 genes and 78 gene families were detected to be contracted and found to have rapidly evolved within the scarlet sage

genome (**Fig. 2c**). Subsequently, 134 orthologous proteins among the 16 analyzed genomes were acquired and aligned with MUSCLE v3.8.31 (MUSCLE, RRID:SCR_011812) [57] employing default settings. A maximum likelihood phylogenetic tree was then generated using the concatenated amino acid sequences in PhyML v3.0 (PhyML, RRID:SCR_014629) [58] with GTR+G+I model. The divergence time was estimated with r8s v1.81 [59] and calibrated against the timing of divergence between *A. thaliana* and *V. vinifera* (124 Mya) [60]as well as against *A. thaliana* and *P. trichocarpa* divergence time (90 Mya) [61]. The phylogenetic analysis identified the close relationship among the three *Salvia* genomes and their divergence time was estimated with about 28.21 Mya (**Fig. 2c**).

Secondary metabolic pathways: gene annotations, gene clusters and comparative genomics

The mint family is recognized as providing promising sources of bioactive secondary metabolites [62]. In fact, a diverse variety of bioactive secondary metabolites can be found with a wide range of pharmacological activities: antimicrobial, antispasmodic, carminative, antioxidant, antiulcer, cytoprotective, heptoprotective, cholagogue, chemo-preventive, anti-inflammatory, antidiabetogenic etc. Here, we obtained enzymatic annotations for coding genes by employing the E2P2 package v3.1 (https://gitlab.com/rhee-lab/E2P2/tree/master). Then, we mapped genes to flavonoid and menthol biosynthesis pathways by querying the Plant Metabolic Network (PMN

v12.5, https://www.plantcyc.org/) [63]. Regarding the flavonoid biosynthesis pathway, we found an abundance of genes encoding annotated enzymes in this pathway, especially of note the 41 genes for flavanone synthase I (EC: 1.14.11.9) (**Figure S5** and **Supplementary_File_1**). With respect to menthol biosynthesis, certain genes are still lacking annotations for enzymes such as (+)-pulegone reductase (EC: 1.3.1.81), (-)-isopiperitenone reductase (EC: 1.3.1.82) or menthol-dehydrogenase (lacking EC number) (**Figure S6** and **Supplementary_File_1**). However, this pathway mapping analysis provides a highly valuable reference for the genetic dissection of key metabolic genes for the scarlet sage.

The presence of metabolic gene clusters for secondary metabolic pathways are common in bacteria and filamentous fungi, and are also widely reported in plants [64-66]. Using the newly created and robust computational toolkit, plantSMASH [67], we identified 85 gene clusters potentially related to secondary metabolic biosynthesis in the scarlet sage genome as reported here, and 23 gene clusters in the *S. miltiorrhiza* genome [8]. Genomic position, gene composition, functional annotation of the identified gene clusters were summarized in **Table S13**, **Supplementary_File_2** and **Supplementary_File_3**. The gene clusters were found to be potentially related to the biosynthesis of alkaloids, saccharides, polyketides, terpenes, and lignans. It was previously reported that physical clustering of terpene synthase genes (TPS) and cytochrome P450 mono-oxygenase genes is frequently associated with consecutive enzymatic actions in terpenoid biosynthesis [68]. Interestingly, we detected eight such

gene clusters within the scarlet sage genome, but none in the *S. miltiorrhiza* genome, which could partially be due to the draft status of the genome assembly for *S. miltiorrhiza*. Furthermore, significant gene co-expression across different organs was detected for one TPS gene and two out of four P450 genes located in a single gene cluster (i.e. Cluster 63; **Table S13** and **Supplementary_File_2**). Evidence for moderate or significant co-expression among clustered genes was revealed and shown in **Supplementary_File_2**.

Based on the collinearity elucidated by former OrthoMCL analyses, a comparative genomic study between the scarlet sage and *S. miltiorrhiza* genomes revealed six pairs of gene clusters which share synteny between these two congeneric plants, and two blocks from the scarlet sage share synteny with one block from *S. miltiorrhiza* (**Figure S7**). Among the shared synteny blocks, four could be related to saccharide, one to lignan and another to polyketide biosynthesis. The smaller number of gene clusters detected for *S. miltiorrhiza* and subsequently, fewer shared synteny blocks of metabolic gene cluster between these two species may be partially attributed to the present state of the *S. miltiorrhiza* genome assembly which is hundred times more fragmented than that of the scarlet sage. Thus, here, we provided a starting point for comparative genomics among plant species within the mint family.

In summary, we presented the draft assembly for the scarlet sage genome using a PacBio long-read dominated strategy, which was responsible for obtaining the high

sequence assembly quality. Also, the almost complete homozygosity within the sequenced inbred line's genome was a key factor for the high continuity gained in this study. The novel genome data generated in the present study will provide a valuable resource for studying the molecular underpinnings of the various phenotypic variation found within *Salvia sp.*, and sets the foundation for molecular-informed breeding strategies and genome editing approaches for this valued ornamental flowering plant. Moreover, this genome assembly is useful for comparative genomic studies among related species.

Availability of supporting data

The genome assembly, annotations, and other supporting data are available via the *GigaScience* database GigaDB [69]. The raw sequence data have been deposited in the Short Read Archive (SRA) under NCBI BioProject ID PRJNA422035.

Abbreviations

AED: Annotation Edit Distance; bp: base pair; BUSCO: benchmarking universal single-copy orthologs; CDS: coding sequence; Gb: gigabases; kb: kilobases; LTR: Long terminal repeats; Mb: megabases; Mya: million years ago; PE: paired end; SMRT: Single-Molecule Real-Time; SNP: Single Nucleotide Polymorphism; TE: TPS: transposable element; terpene synthase genes.

Acknowledgement

This study was funded by Beijing Key Laboratory of Greening Plants Breeding (NO. Z201605) and Fundamental Research Funds for the Central Universities (NO. YX2013-41).

Author Contributions

AXD, HBX, RCC, JFM, FM and IP conceived and designed the study; AXD, HBX, ZJL, HL, YQS, SN, ZNZ, RFC, HLZ, RGZ and QZY prepared the materials and conducted the experiments; JFM, HBX, FM, IP wrote the manuscript.

Conflict of Interest

The authors declare that they have no competing financial interests.

References

1. Drew BT, González-Gallegos JG, Xiang C-L, Kriebel R, Drummond CP, Walker JB, et al. Salvia united: The greatest good for the greatest number. Taxon. 2017;66 1:133-45.
2. Sutton J. The Gardener's Guide to Growing Salvias. David & Charles; 1999.
3. Clebsch B and Barner CD. The New Book of Salvias: Sages for Every Garden. Timber Press; 2003.
4. Walker JB, Sytsma KJ, Treutlein J and Wink M. *Salvia* (Lamiaceae) is not monophyletic: implications for the systematics, radiation, and ecological specializations of *Salvia* and tribe Mentheae. American Journal of Botany. 2004;91 7:1115-25.
5. Griffiths M and Society RH. Index of Garden Plants. Macmillan; 1994.
6. Regnault-Roger C. The potential of botanical essential oils for insect pest control. Integrated Pest Management Reviews. 1997;2 1:25-34.
7. Ge X, Chen H, Wang H, Shi A and Liu K. De Novo Assembly and Annotation

of *Salvia splendens* Transcriptome Using the Illumina Platform. PLoS One. 2014;9 3:e87693.

8. Zhang G, Tian Y, Zhang J, Shu L, Yang S, Wang W, et al. Hybrid de novo genome assembly of the Chinese herbal plant danshen (*Salvia miltiorrhiza* Bunge). GigaScience. 2015;4 1:62.

9. Xu H, Song J, Luo H, Zhang Y, Li Q, Zhu Y, et al. Analysis of the Genome Sequence of the Medicinal Plant *Salvia miltiorrhiza*. Molecular Plant. 2016;9 6:949-52.

10. Vining KJ, Johnson SR, Ahkami A, Lange I, Parrish AN, Trapp SC, et al. Draft Genome Sequence of *Mentha longifolia* and Development of Resources for Mint Cultivar Improvement. Molecular Plant. 2017;10 2:323-39.

11. Bolger AM, Lohse M and Usadel B. Trimmomatic: a flexible trimmer for Illumina sequence data. Bioinformatics. 2014;30 15:2114-20.

12. Alberto CM, Sanso AM and Xifreda CC. Chromosomal studies in species of *Salvia* (Lamiaceae) from Argentina. Botanical Journal of the Linnean Society. 2003;141 4:483-90.

13. Martin M. Cutadapt removes adapter sequences from high-throughput sequencing reads. EMBnetjournal. 2011;17 1: 10-12.

14. The Gene Ontology (GO) database and informatics resource. Nucleic Acids Research. 2004;32 Database issue:D258-D61.

15. Koren S, Walenz BP, Berlin K, Miller JR, Bergman NH and Phillippy AM. Canu: scalable and accurate long-read assembly via adaptive k-mer weighting and repeat separation. Genome Research. 2017; 722-736.

16. Marçais G and Kingsford C. A fast, lock-free approach for efficient parallel counting of occurrences of k-mers. Bioinformatics. 2011;27 6:764-70.

17. Liu B, Shi Y, Yuan J, Hu X, Zhang H, Li N, et al. Estimation of genomic characteristics by analyzing k-mer frequency in de novo genome projects. arXiv preprint arXiv:13082012. 2013.

18. Xiao C-L, Chen Y, Xie S-Q, Chen K-N, Wang Y, Han Y, et al. MECAT: fast mapping, error correction, and de novo assembly for single-molecule sequencing reads. Nature Methods. 2017;14:1072.

19. Chin CS, Peluso P, Sedlazeck FJ, Nattestad M, Concepcion GT, Clum A, et al. Phased diploid genome assembly with single-molecule real-time sequencing. Nature Methods. 2016;13 12:1050-4.

20. Simão FA, Waterhouse RM, Ioannidis P, Kriventseva EV and Zdobnov EM. BUSCO: assessing genome assembly and annotation completeness with single-copy orthologs. Bioinformatics. 2015;31 19:3210-2.

21. Boetzer M and Pirovano W. SSPACE-LongRead: scaffolding bacterial draft genomes using long read sequence information. BMC Bioinformatics. 2014;15 1:211.

22. Boetzer M, Henkel CV, Jansen HJ, Butler D and Pirovano W. Scaffolding pre-assembled contigs using SSPACE. Bioinformatics. 2011;27 4:578-9.

23. Luo R, Liu B, Xie Y, Li Z, Huang W, Yuan J, et al. SOAPdenovo2: an empirically improved memory-efficient short-read de novo assembler. GigaScience. 2012;1:18.

24. Langmead B and Salzberg SL. Fast gapped-read alignment with Bowtie 2. Nature Methods. 2012;9 4:357-9.

25. Bao W, Kojima KK and Kohany O. Repbase Update, a database of repetitive elements in eukaryotic genomes. Mobile DNA. 2015;6 1:11.

26. Kim D, Langmead B and Salzberg SL. HISAT: a fast spliced aligner with low memory requirements. Nature Methods. 2015;12 4:357-60.

27. Trapnell C, Williams BA, Pertea G, Mortazavi A, Kwan G, van Baren MJ, et al. Transcript assembly and quantification by RNA-Seq reveals unannotated transcripts and isoform switching during cell differentiation. Nature Biotechnology. 2010;28 5:511-5.

28. Pertea M, Pertea GM, Antonescu CM, Chang T-C, Mendell JT and Salzberg SL. StringTie enables improved reconstruction of a transcriptome from RNA-seq reads. Nature Biotechnology. 2015;33 3:290-5.

29. Grabherr MG, Haas BJ, Yassour M, Levin JZ, Thompson DA, Amit I, et al. Full-length transcriptome assembly from RNA-Seq data without a reference genome. Nature Biotechnology. 2011;29:644.

30. Fu L, Niu B, Zhu Z, Wu S and Li W. CD-HIT: accelerated for clustering the next-generation sequencing data. Bioinformatics. 2012;28 23:3150-2.

31. Stanke M, Diekhans M, Baertsch R and Haussler D. Using native and syntenically mapped cDNA alignments to improve de novo gene finding. Bioinformatics. 2008;24 5:637-44.

32. Boratyn GM, Schäffer AA, Agarwala R, Altschul SF, Lipman DJ and Madden TL. Domain enhanced lookup time accelerated BLAST. Biology Direct. 2012;7 1:12.

33. Slater GSC and Birney E. Automated generation of heuristics for biological sequence comparison. BMC Bioinformatics. 2005; 6 1:1-11.

34. Cantarel BL, Korf I, Robb SM, Parra G, Ross E, Moore B, et al. MAKER: an easy-to-use annotation pipeline designed for emerging model organism genomes. Genome Research. 2008;18 1:188-96.

35. Kent WJ. BLAT--the BLAST-like alignment tool. Genome Research. 2002;12 4:656-64.

36. Bairoch A and Apweiler R. The SWISS-PROT protein sequence database and its supplement TrEMBL in 2000. Nucleic Acids Research. 2000;28 1:45-8.

37. Bateman A, Birney E, Cerruti L, Durbin R, Etwiller L, Eddy SR, et al. The Pfam Protein Families Database. Nucleic Acids Research. 2002;30 1:276-80.

38. Quevillon E, Silventoinen V, Pillai S, Harte N, Mulder N, Apweiler R, et al. InterProScan: protein domains identifier. Nucleic Acids Research. 2005;33 Web Server issue:W116-W20.

39. Kanehisa M and Goto S. KEGG: Kyoto Encyclopedia of Genes and Genomes.

Nucleic Acids Research. 2000;28 1:27-30.
40. The Gene Ontology C, Ashburner M, Ball CA, Blake JA, Botstein D, Butler H, et al. Gene Ontology: tool for the unification of biology. Nature Genetics. 2000;25 1:25-9.
41. Sollars ESA, Harper AL, Kelly LJ, Sambles CM, Ramirez-Gonzalez RH, Swarbreck D, et al. Genome sequence and genetic diversity of European ash trees. Nature. 2016;541:212.
42. Unver T, Wu Z, Sterck L, Turktas M, Lohaus R, Li Z, et al. Genome of wild olive and the evolution of oil biosynthesis. Proceedings of the National Academy of Sciences. 2017;114 44:E9413-E22.
43. Hellsten U, Wright KM, Jenkins J, Shu S, Yuan Y, Wessler SR, et al. Fine-scale variation in meiotic recombination in *Mimulus* inferred from population shotgun sequencing. Proceedings of the National Academy of Sciences. 2013;110 48:19478-82.
44. Lan T, Renner T, Ibarra-Laclette E, Farr KM, Chang T-H, Cervantes-Pérez SA, et al. Long-read sequencing uncovers the adaptive topography of a carnivorous plant genome. Proceedings of the National Academy of Sciences. 2017;114 22:E4435-E41.
45. Wang L, Yu S, Tong C, Zhao Y, Liu Y, Song C, et al. Genome sequencing of the high oil crop sesame provides insight into oil biosynthesis. Genome Biology. 2014;15 2:R39.
46. Denoeud F, Carretero-Paulet L, Dereeper A, Droc G, Guyot R, Pietrella M, et al. The coffee genome provides insight into the convergent evolution of caffeine biosynthesis. Science. 2014;345 6201:1181-4.
47. Tomato Genome Consortium. The tomato genome sequence provides insights into fleshy fruit evolution. Nature. 2012;485:635.
48. Iorizzo M, Ellison S, Senalik D, Zeng P, Satapoomin P, Huang J, et al. A high-quality carrot genome assembly provides new insights into carotenoid accumulation and asterid genome evolution. Nature Genetics. 2016;48:657.
49. The French-Italian Public Consortium for Grapevine Genome Consortium for Grapevine Genome Characterization. The grapevine genome sequence suggests ancestral hexaploidization in major angiosperm phyla. Nature. 2007;449:463.
50. Cheng C-Y, Krishnakumar V, Chan AP, Thibaud-Nissen F, Schobel S and Town CD. Araport11: a complete reannotation of the *Arabidopsis thaliana* reference genome. The Plant Journal. 2017;89 4:789-804.
51. Tuskan GA, DiFazio S, Jansson S, Bohlmann J, Grigoriev I, Hellsten U, et al. The Genome of Black Cottonwood, *Populus trichocarpa* (Torr. & Gray). Science. 2006;313 5793:1596-604.
52. Ouyang S, Zhu W, Hamilton J, Lin H, Campbell M, Childs K, et al. The TIGR Rice Genome Annotation Resource: improvements and new features. Nucleic Acids Research. 2007;35 suppl_1:D883-D7.

53. Dohm JC, Minoche AE, Holtgräwe D, Capella-Gutiérrez S, Zakrzewski F, Tafer H, et al. The genome of the recently domesticated crop plant sugar beet (*Beta vulgaris*). Nature. 2013;505:546.
54. Li L, Stoeckert CJ and Roos DS. OrthoMCL: Identification of Ortholog Groups for Eukaryotic Genomes. Genome Research. 2003;13 9:2178-89.
55. De Bie T, Cristianini N, Demuth JP and Hahn MW. CAFE: a computational tool for the study of gene family evolution. Bioinformatics. 2006;22 10:1269-71.
56. Badouin H, Gouzy J, Grassa CJ, Murat F, Staton SE, Cottret L, et al. The sunflower genome provides insights into oil metabolism, flowering and Asterid evolution. Nature. 2017;546:148.
57. Edgar RC. MUSCLE: multiple sequence alignment with high accuracy and high throughput. Nucleic Acids Research. 2004;32 5:1792-7.
58. Guindon S, Dufayard J-F, Lefort V, Anisimova M, Hordijk W and Gascuel O. New Algorithms and Methods to Estimate Maximum-Likelihood Phylogenies: Assessing the Performance of PhyML 3.0. Systematic Biology. 2010;59 3:307-21.
59. Sanderson MJ. r8s: inferring absolute rates of molecular evolution and divergence times in the absence of a molecular clock. Bioinformatics. 2003;19 2:301-2.
60. Doyle JA. Molecular and Fossil Evidence on the Origin of Angiosperms. Annual Review of Earth and Planetary Sciences. 2012;40 1:301-26.
61. Wang H, Moore MJ, Soltis PS, Bell CD, Brockington SF, Alexandre R, et al. Rosid radiation and the rapid rise of angiosperm-dominated forests. Proceedings of the National Academy of Sciences. 2009;106 10:3853-8.
62. Mimica-Dukic N and Bozin B. *Mentha* L. Species (Lamiaceae) as Promising Sources of Bioactive Secondary Metabolites. Current Pharmaceutical Design. 2008;14 29:3141-50.
63. Schläpfer P, Zhang P, Wang C, Kim T, Banf M, Chae L, et al. Genome-Wide Prediction of Metabolic Enzymes, Pathways, and Gene Clusters in Plants. Plant Physiology. 2017;173 4:2041-59.
64. Osbourn A. Secondary metabolic gene clusters: evolutionary toolkits for chemical innovation. Trends in Genetics. 2010;26 10:449-57.
65. Nützmann H-W and Osbourn A. Gene clustering in plant specialized metabolism. Current Opinion in Biotechnology. 2014;26:91-9.
66. Hans-Wilhelm N, Ancheng H and Anne O. Plant metabolic clusters – from genetics to genomics. New Phytologist. 2016;211 3:771-89.
67. Kautsar SA, Suarez Duran HG, Blin K, Osbourn A and Medema MH. plantiSMASH: automated identification, annotation and expression analysis of plant biosynthetic gene clusters. Nucleic Acids Research. 2017;45 W1:W55-W63.

68. Boutanaev AM, Moses T, Zi J, Nelson DR, Mugford ST, Peters RJ, et al. Investigation of terpene diversification across multiple sequenced plant genomes. Proceedings of the National Academy of Sciences. 2015;112 1:E81-E8.

69. Dong, A; Xin, H; Li, Z; Liu, H; Sun, Y; Nie, S; Zhao, Z; Cui, R; Zhang, R; Yun, Q; Wang, X; Maghuly, F; Porth, I; Cong, R; Mao, J (2018): Supporting data for "High quality assembly of the reference genome for scarlet sage, Salvia splendens, an economically important ornamental plant" GigaScience Database. http://dx.doi.org/10.5524/100463.

Supplementary Figures

Figure S1. Length distribution of PacBio subreads.

Figure S2. K-mer frequency distribution at k-mer size of 17. A k-mer refers to an artificial sequence division of K nucleotides. From k-mer frequency, genomic characteristics (genome size, repeat structure and heterozygous rate) could be estimated. Peaks at depths of 31 and 62 were annotated with dash lines.

Figure S3. Distribution of AED scores from gene prediction. AED, Annotation Edit Distance, AED = 0 indicates perfect agreement between annotation and the evidence; AED = 1 indicates no evidence support for annotation.

Figure S4. Length distribution of annotated genes, exons and introns. **a-c** for annotated genes, exons and introns from different genome assemblies.

Figure S5. Flavonoid biosynthesis pathway. Flavonoid biosynthesis pathways by querying the Plant Metabolic Network (https://www.plantcyc.org/), enzymatic coding genes of the scarlet sage were shown for key reactions.

Figure S6. Menthol biosynthesis pathway. Menthol biosynthesis pathways by querying the Plant Metabolic Network (https://www.plantcyc.org/), enzymatic coding genes of the scarlet sage were shown for key reactions.

Figure S7. Shared synteny addressed for metabolic gene clusters between Salvia genomes. a-f: display of the different pairs of synteny blocks. Genes are colored along the contigs/scaffolds to compare between scarlet sage and *Salvia miltiorrhiza* Zhang [8], with metabolic genes highlighted with olive drab color, other homologous genes

are shown in grey.

Supplementary Tables

Table S1. Summary of Pacbio and Illumina sequencing data generated in the present study. IDs of the study, sample, library and accession in NCBI SRA and sequencing platform, material origins of the sequenced DNA or RNA, the statistics of the raw and cleaned data are shown.

Table S2. Estimation of genome characteristics based on 17-mer statistics.

Table S3. Statistics of the different versions of the genome assembly of the scarlet sage. NA: data not available; * statistics for contigs/scaffolds.

Table S4. Summary of the annotated interspersed repeats in the genome assembly of the scarlet sage.

Table S5. Summary of the transcriptome assemblies.

Table S6. Summary of the annotated genes. AED: Annotation Edit Distance; gene regions (including UTRs, exons and introns); genes (including 5’, 3’ UTRs, exons and introns).

Table S7. Summary of BUSCO evaluation of gene prediction.

Table S8. Summary of functional annotation of predicted genes.

Table S9. Genomic data used for gene families analyses. Origins, download links, assembly versions, genome properties and references of 15 analyzed genomes are shown.

Table S10. Summary of gene family analyses. Unique groups and genes, single-copy and duplicated groups and genes are summarized for the 16 analyzed genomes of 15 plant species.

Table S11. GO enrichment of expanded gene families. (A) ‘Category’ is the Gene Ontology (GO) term ID; (B) ‘p_value’ is the over represented p-value indicating the observed frequency of a given term among analyzed genes is equal to the expected frequency based on the null distribution; i.e. lower p-values indicate stronger evidence for overrepresentation; (C) ‘q_value’ is the Benjamini and Hochberg adjusted p-value, (D) ‘numEPInCat’ is the number of expanded gene families in the corresponding GO category; (E) ‘numInCat’ is the number of detected gene families in the corresponding GO category; (F) ‘Term’ is the GO term; (G) ‘Ontology’ indicates which ontology the term comes from. 60 significant ($q<0.05$) GO-terms of three different functional categories are indicated in bold.

Table S12. KEGG enrichment of expanded gene families. (A) ‘KO category’ is the KEGG Orthology (KO) category ID; (B) ‘p_value’ is the over represented p-value indicating the observed frequency of a given term among analyzed genes is equal to the expected frequency based on the null distribution; i.e. lower p-values indicate stronger evidence for overrepresentation; (C) ‘q_value’ is the Benjamini and Hochberg adjusted p-value, (D) ‘numEPInCat’ is the number of expanded gene families in the corresponding KO category; (E) ‘numInCat’ is the number of detected gene families in the corresponding KO category; (F) ‘Pathway’ is the KEGG pathway;

(G) ‘Class’ indicates which KEGG class the pathway comes from. One significant (q<0.05) KEGG pathway is indicated in bold.

Table S13. Summary of metabolic gene clusters detected in genomes of *Salvia miltiorrhiza* and *S. splendens*. (A) ‘Genome’ denotes the genome origination; (B) ‘Cluster’ is the code for a certain gene cluster detected; (C) ‘Record’ denotes the contig/scaffold ID from where the gene cluster was detected; (D) ‘Type’ denotes the functional assignment for the gene cluster; (E) ‘From’, ‘To’ and ‘Size’ denote the genomic position and range of the gene cluster; (F) ‘Core domains’ denote the domain annotation for the metabolic genes in the cluster; (G) ‘CD-HIT Cluster’ indicate the number of genes in the cluster; (H) ‘Gene cluster genes’ is showing the ID of genes in the cluster.

Supplementary Files

Supplementary File 1. Genes (Gene ID, name and EC number) mapped to flavonoid and menthol biosynthesis pathways.

Supplementary File 2. Structure of a metabolic gene cluster (polyketide synthesis) and gene expression patterns of *Salvia splendens*. Genomic position, gene composition, functional annotation of gene cluster are shown, also including a heatmap of tissue specific expression of the genes within the presented cluster is shown. HG: root of red flower (individual); HJ: stem of red flower (individual); HY: leave of red flower (individual); HE: calyx of red flower (individual); HHG: corolla

of red flower (individual); ZG: root of purple flower (individual); ZJ: stem of purple flower (individual); ZY: leave of purple flower (individual); ZE: calyx of purple flower (individual); ZHG: corolla of purple flower (individual).

Supplementary File 3. Structure of a metabolic gene cluster (alkaloid synthesis). Genomic position, gene composition, functional annotation of gene cluster were shown.

Tables

Table 1. Statistics of the final genome assembly of the scarlet sage.

	Contig		Scaffold	
	**Size (bp)**	**Number**	**Size (bp)**	**Number**
**Total Size**	807,514,799	-	809,159,598	-
**Total Number**	-	2,204	-	1,525
**N10**	6,529,455	10	8,157,631	9
**N50**	2,267,074	100	3,123,266	73
**N90**	265,262	456	433,303	324
**Max.**	10,812,588	-	12,944,193	-
**Min.**	500	-	9,495	-
**Mean**	366,386	-	530,596	-
**Median**	38,049	-	48,557	-
**Gap**	-	-	1,644,799 (0.2%)	679
**GC Content**	38.84%	-	38.76%	-

Figures:

Fig. 1 Images of the scarlet sage, *Salvia splendens.*

a-b, flowers of the sequenced cultivar of *S. splendens*, "Aoyunshenghuo (Olympic flame)"; **c**, the scarlet sage with different flower colors in bedding; **d-k**, the scarlet sage with flowers of different pure colors or bi-colors.

Fig. 2 Quality of scarlet sage genome assembly and the phylogenomic inferences. Quality was assessed by comparing the scarlet genome with the recently released genomes of related species. Length distribution of contigs (**a**) and scaffolds (**b**); **c**, phylogenetic tree, divergence time, and profiles of gene families that underwent expansion or contraction. *Salvia miltiorrhiza* Zhang [14] and *Salvia miltiorrhiza* Xu [14] are two genome assemblies reported for *Salvia miltiorrhiza*.

Fig. 1

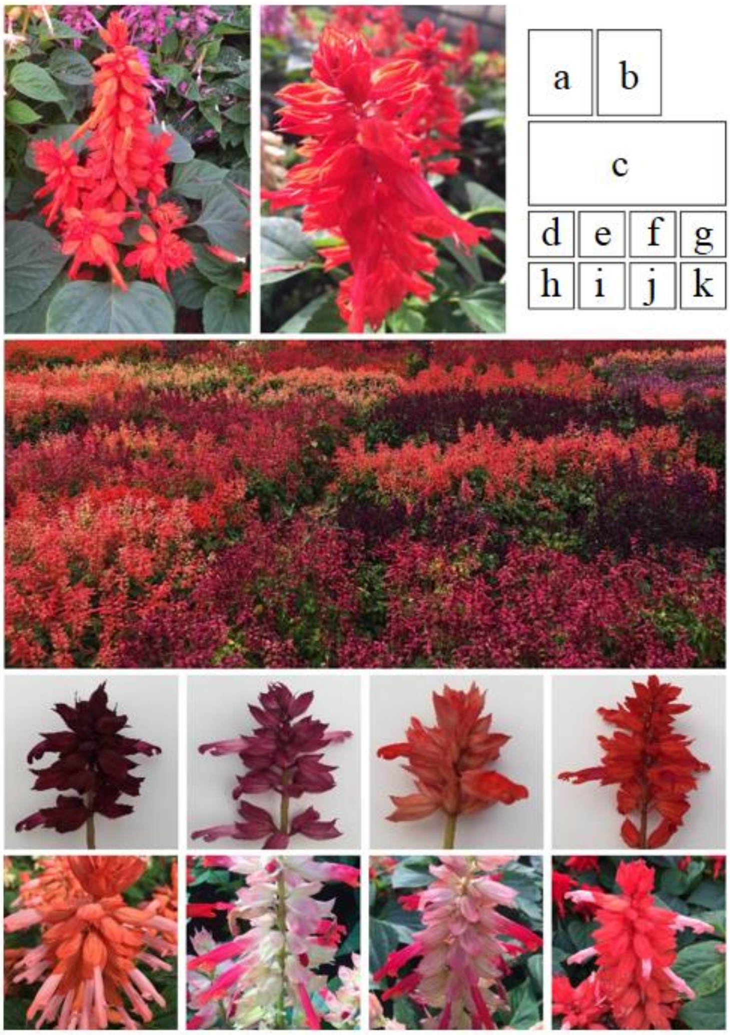

Fig. 2

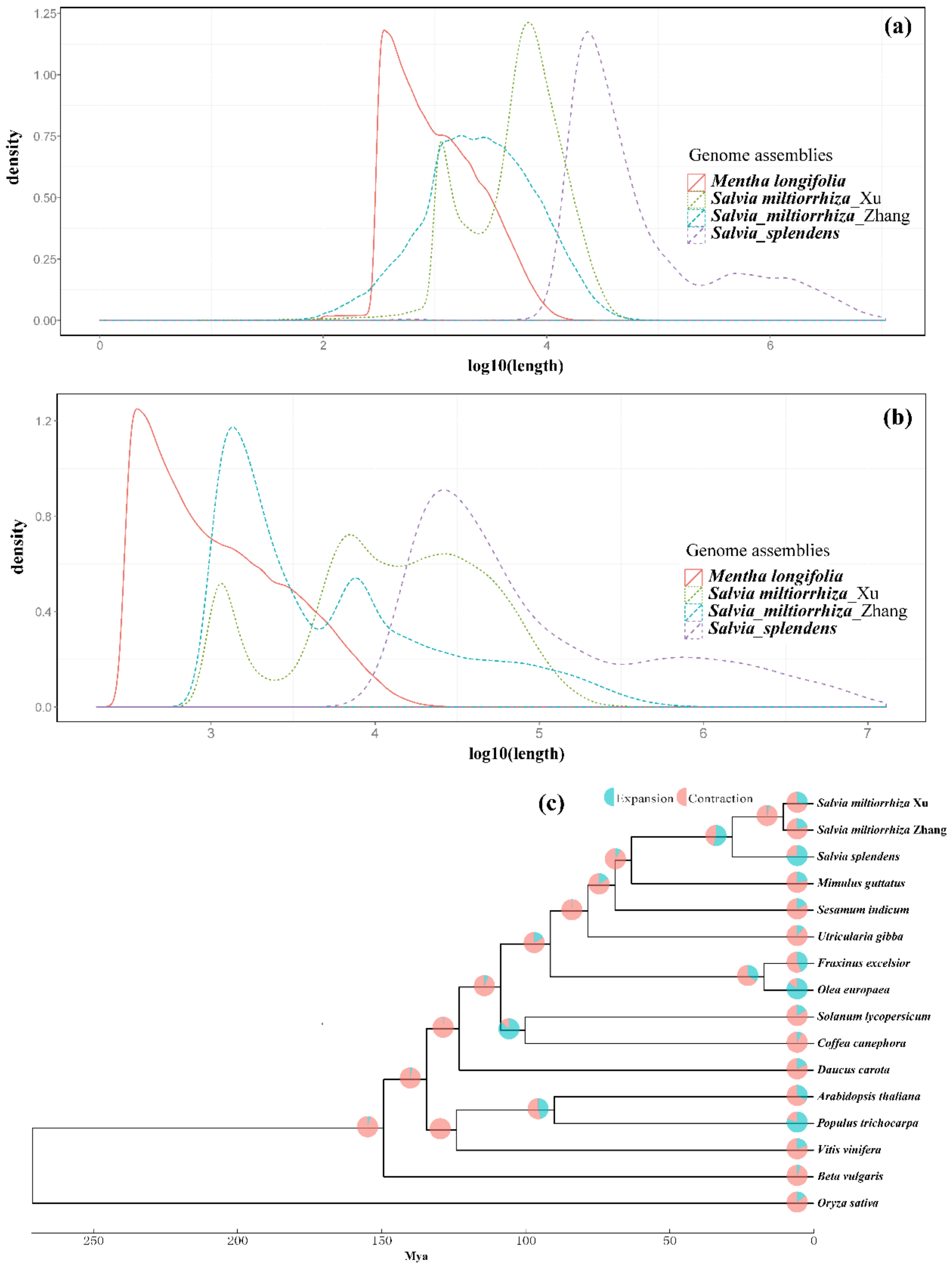
(a)
density
log10(length)
Genome assemblies
Mentha longifolia
Salvia miltiorrhiza_Xu
Salvia_miltiorrhiza_Zhang
Salvia_splendens
(b)
density
log10(length)
Genome assemblies
Mentha longifolia
Salvia miltiorrhiza_Xu
Salvia_miltiorrhiza_Zhang
Salvia_splendens
(c)
Expansion
Contraction
Salvia miltiorrhiza Xu
Salvia miltiorrhiza Zhang
Salvia splendens
Mimulus guttatus
Sesamum indicum
Utricularia gibba
Fraxinus excelsior
Olea europaea
Solanum lycopersicum
Coffea canephora
Daucus carota
Arabidopsis thaliana
Populus trichocarpa
Vitis vinifera
Beta vulgaris
Oryza sativa
Mya

Click here to access/download
Supplementary Material
Table_S1.xlsx

Click here to access/download
Supplementary Material
Table_S2.docx

Click here to access/download
Supplementary Material
Table_S3.docx

Click here to access/download
Supplementary Material
Table_S4.docx

Click here to access/download
Supplementary Material
Table_S5.docx

Click here to access/download
Supplementary Material
Table_S6.docx

Click here to access/download
Supplementary Material
Table_S7.docx

Click here to access/download
Supplementary Material
Table_S8.docx

Click here to access/download
Supplementary Material
Table_S9.docx

Click here to access/download
Supplementary Material
Table_S10.docx

Click here to access/download
Supplementary Material
Table_S11.xlsx

Click here to access/download
Supplementary Material
Table_S12.docx

Click here to access/download
Supplementary Material
Table_S13.xlsx

Click here to access/download
Supplementary Material
Fig_S1.pdf

Click here to access/download
Supplementary Material
Fig_S2.pdf

Click here to access/download
Supplementary Material
Fig_S3.pdf

Click here to access/download
Supplementary Material
Fig_S4.pdf

Click here to access/download
Supplementary Material
Fig_S5.pdf

Click here to access/download
Supplementary Material
Fig_S6.pdf

Click here to access/download
Supplementary Material
Fig_S7.pdf

Click here to access/download
Supplementary Material
Supplementary_File_1.docx

Click here to access/download
Supplementary Material
Supplementary_File_2.docx

Click here to access/download
Supplementary Material
Supplementary_File_3.docx
