# Supplementary material for: High-quality assembly of the reference genome for scarlet sage, Salvia splendens, an economically important ornamental plant
Source: Gigascience. 2018 Jun 19;7(7):giy068. doi: 10.1093/gigascience/giy068 (PMC6030905; doi:10.1093/gigascience/giy068)
Supplement: Additional Files [file giy068_supplemental_files.zip › Fig_S4.pdf]

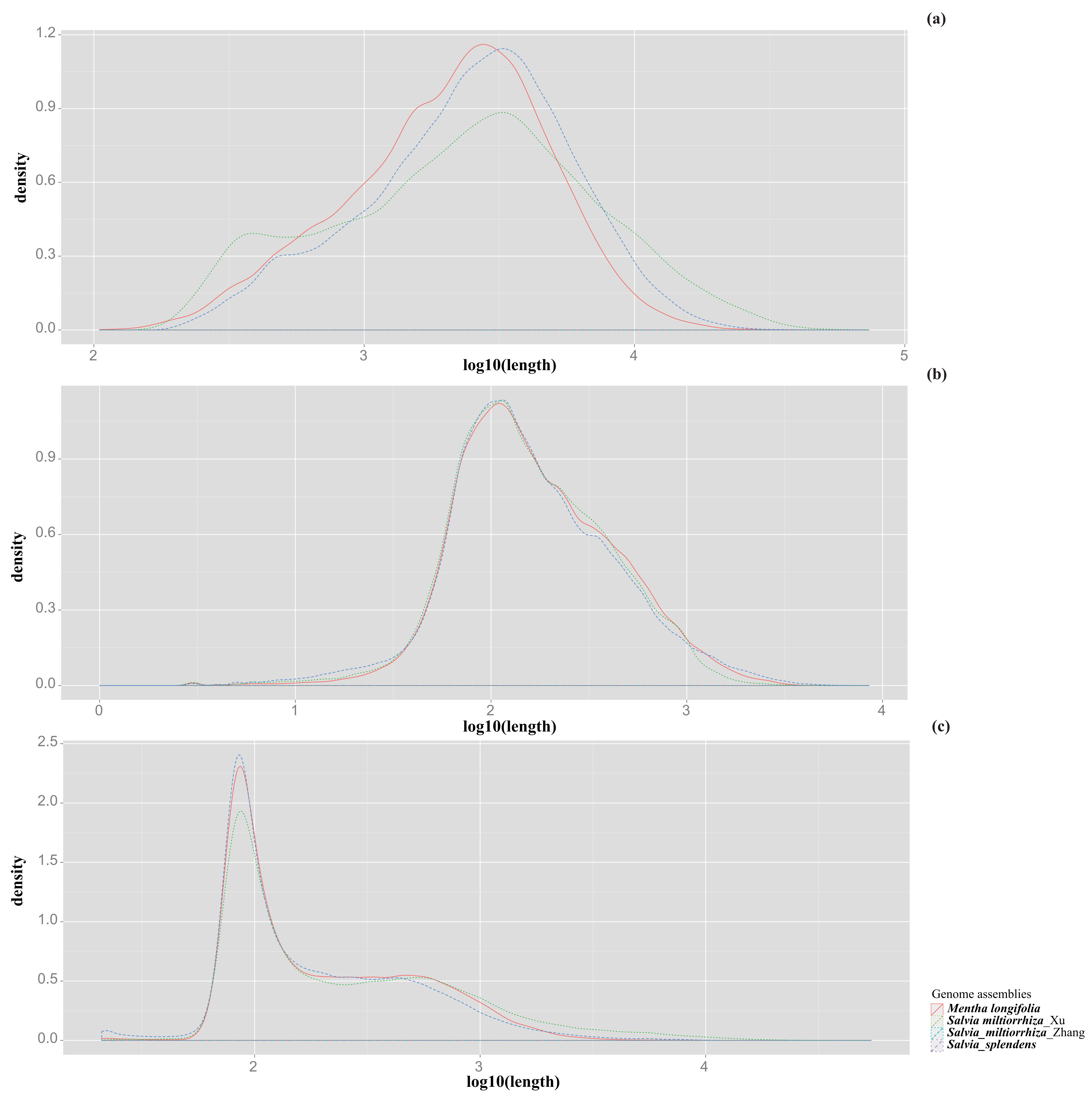

(a)
1.2
0.9
0.6
0.3
0.0
density
2
3
4
5
log10(length)
(b)
0.9
0.6
0.3
0.0
density
0
1
2
3
4
log10(length)
(c)
2.5
2.0
1.5
1.0
0.5
0.0
density
2
3
4
log10(length)
Genome assemblies
Mentha longifolia
Salvia miltiorrhiza_Xu
Salvia_miltiorrhiza_Zhang
Salvia_splendens
