# Supplementary material for: High-quality assembly of the reference genome for scarlet sage, Salvia splendens, an economically important ornamental plant
Source: Gigascience. 2018 Jun 19;7(7):giy068. doi: 10.1093/gigascience/giy068 (PMC6030905; doi:10.1093/gigascience/giy068)
Supplement: Additional Files [file giy068_supplemental_files.zip › Fig_S6.pdf]

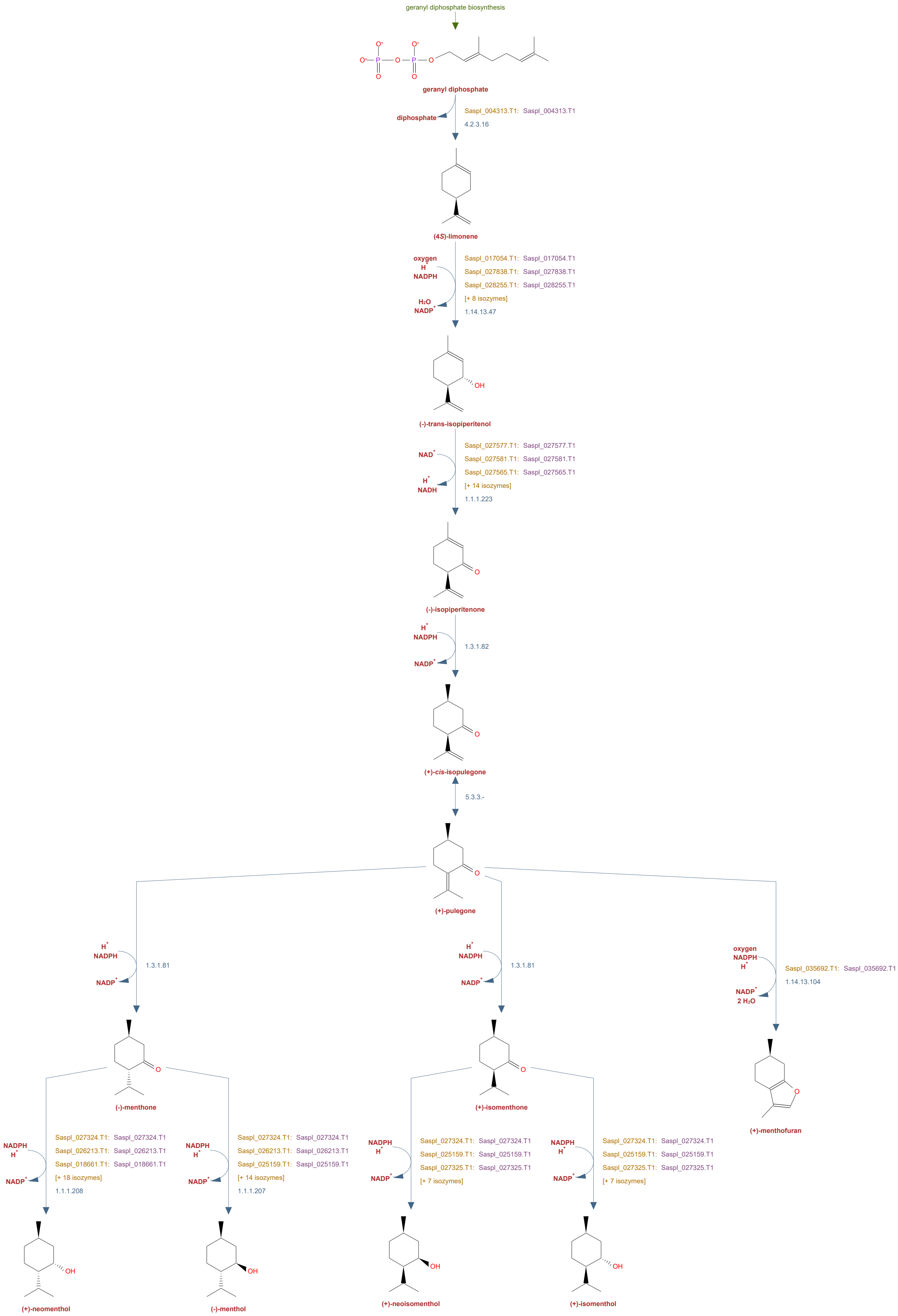

geranyl diphosphate biosynthesis
geranyl diphosphate
diphosphate
Saspl_004313.T1: Saspl_004313.T1
4.2.3.16
(4S)-limonene
oxygen
H+
NADPH
H2O
NADP+
Saspl_017054.T1: Saspl_017054.T1
Saspl_027838.T1: Saspl_027838.T1
Saspl_028255.T1: Saspl_028255.T1
[+ 8 isozymes]
1.14.13.47
(-)-trans-isopiperitenol
NAD+
H+
NADH
Saspl_027577.T1: Saspl_027577.T1
Saspl_027581.T1: Saspl_027581.T1
Saspl_027565.T1: Saspl_027565.T1
[+ 14 isozymes]
1.1.1.223
(-)-isopiperitenone
H+
NADPH
NADP+
1.3.1.82
(+)-cis-isopulegone
5.3.3.-
(+)-pulegone
H+
NADPH
NADP+
1.3.1.81
H+
NADPH
NADP+
1.3.1.81
oxygen
NADPH
H+
NADP+
2 H2O
Saspl_035692.T1: Saspl_035692.T1
1.14.13.104
(-)-menthone
(+)-isomenthone
(+)-menthofuran
NADPH
H+
NADP+
Saspl_027324.T1: Saspl_027324.T1
Saspl_026213.T1: Saspl_026213.T1
Saspl_018661.T1: Saspl_018661.T1
[+ 18 isozymes]
1.1.1.208
NADPH
H+
NADP+
Saspl_027324.T1: Saspl_027324.T1
Saspl_026213.T1: Saspl_026213.T1
Saspl_025159.T1: Saspl_025159.T1
[+ 14 isozymes]
1.1.1.207
NADPH
H+
NADP+
Saspl_027324.T1: Saspl_027324.T1
Saspl_025159.T1: Saspl_025159.T1
Saspl_027325.T1: Saspl_027325.T1
[+ 7 isozymes]
NADPH
H+
NADP+
Saspl_027324.T1: Saspl_027324.T1
Saspl_025159.T1: Saspl_025159.T1
Saspl_027325.T1: Saspl_027325.T1
[+ 7 isozymes]
(+)-neomenthol
(-)-menthol
(+)-neoisomenthol
(+)-isomenthol
