# Supplementary material for: High-quality assembly of the reference genome for scarlet sage, Salvia splendens, an economically important ornamental plant
Source: Gigascience. 2018 Jun 19;7(7):giy068. doi: 10.1093/gigascience/giy068 (PMC6030905; doi:10.1093/gigascience/giy068)
Supplement: Additional Files [file giy068_supplemental_files.zip › Supplementary_File_1.docx]

ID Saspl_022367.T1

METACYC RXN1F-93

EC 1.14.11.23

--

//

ID Saspl_005419.T1

NAME Saspl_005419.T1

EC 1.14.11.23

--

//

ID Saspl_012839.T1

NAME Saspl_012839.T1

EC 1.14.11.23

--

//

ID Saspl_014790.T1

NAME Saspl_014790.T1

EC 1.14.11.23

--

//

ID Saspl_004726.T1

NAME Saspl_004726.T1

EC 1.14.11.23

--

//

ID Saspl_002704.T1

NAME Saspl_002704.T1

EC 1.14.11.23

--

//

ID Saspl_045007.T1

NAME Saspl_045007.T1

EC 1.14.11.23

--

//

ID Saspl_010237.T1

NAME Saspl_010237.T1

EC 1.14.11.23

--

//

ID Saspl_024043.T1

NAME Saspl_024043.T1

EC 1.14.11.23

--

NAME Saspl_000236.T1

METACYC 1.14.11.20-RXN

EC 1.14.11.20

EC 1.14.11.23

--

//

ID Saspl_022737.T1

NAME Saspl_022737.T1

EC 1.14.11.23

//

ID Saspl_010292.T1

NAME Saspl_010292.T1

EC 1.14.11.9

--

//

ID Saspl_024039.T1

NAME Saspl_024039.T1

EC 1.14.11.9

--

//

ID Saspl_037558.T1

NAME Saspl_037558.T1

EC 1.14.11.9

--

//

ID Saspl_028602.T1

NAME Saspl_028602.T1

EC 1.14.11.9

--

//

ID Saspl_024046.T1

NAME Saspl_024046.T1

EC 1.14.11.9

--

//

ID Saspl_024040.T1

NAME Saspl_024040.T1

EC 1.14.11.9

--

//

ID Saspl_049245.T1

NAME Saspl_049245.T1

EC 1.14.11.9

--

//

ID Saspl_040901.T1

NAME Saspl_040901.T1

EC 1.14.11.9

--

//

ID Saspl_024041.T1

NAME Saspl_024041.T1

EC 1.14.11.9

--

//

ID Saspl_050111.T1

NAME Saspl_050111.T1

EC 1.14.11.9

--

//

ID Saspl_049246.T1

NAME Saspl_049246.T1

EC 1.14.11.9

--

//

ID Saspl_052487.T1

NAME Saspl_052487.T1

EC 1.14.11.9

--

//

ID Saspl_002703.T1

NAME Saspl_002703.T1

EC 1.14.11.9

--

//

ID Saspl_025216.T1

NAME Saspl_025216.T1

EC 1.14.11.9

--

//

ID Saspl_040897.T1

NAME Saspl_040897.T1

EC 1.14.11.9

--

//

ID Saspl_010013.T1

NAME Saspl_010013.T1

EC 1.14.11.9

--

ID Saspl_005419.T1

NAME Saspl_005419.T1

EC 1.14.11.23

EC 1.14.11.9

--

//

ID Saspl_034777.T1

NAME Saspl_034777.T1

EC 1.14.11.9

--

//

ID Saspl_040899.T1

NAME Saspl_040899.T1

EC 1.14.11.9

--

//

ID Saspl_020700.T1

NAME Saspl_020700.T1

EC 1.14.11.9

--

//

ID Saspl_012726.T1

NAME Saspl_012726.T1

EC 1.14.11.9

--

//

ID Saspl_049252.T1

NAME Saspl_049252.T1

EC 1.14.11.9

--

//

ID Saspl_036942.T1

NAME Saspl_036942.T1

EC 1.14.11.9

--

//

ID Saspl_026966.T1

NAME Saspl_026966.T1

EC 1.14.11.9

--

//

ID Saspl_028225.T1

NAME Saspl_028225.T1

EC 1.14.11.9

--

ID Saspl_045007.T1

NAME Saspl_045007.T1

EC 1.14.11.23

EC 1.14.11.9

--

//

ID Saspl_010014.T1

NAME Saspl_010014.T1

EC 1.14.11.9

--

//

ID Saspl_040896.T1

NAME Saspl_040896.T1

EC 1.14.11.9

--

//

ID Saspl_004620.T1

NAME Saspl_004620.T1

EC 1.14.11.9

--

//

ID Saspl_036943.T1

NAME Saspl_036943.T1

EC 1.14.11.9

--

//

ID Saspl_001236.T1

NAME Saspl_001236.T1

EC 1.14.11.9

--

//

ID Saspl_013644.T1

NAME Saspl_013644.T1

EC 1.14.11.9

--

//

ID Saspl_005694.T1

NAME Saspl_005694.T1

EC 1.14.11.9

--

//

ID Saspl_012949.T1

NAME Saspl_012949.T1

EC 1.14.11.9

--

//

ID Saspl_005088.T1

NAME Saspl_005088.T1

EC 1.14.11.9

--

//

ID Saspl_053022.T1

NAME Saspl_053022.T1

EC 1.14.11.9

--

//

ID Saspl_039509.T1

NAME Saspl_039509.T1

EC 1.14.11.9

--

//

ID Saspl_034776.T1

NAME Saspl_034776.T1

EC 1.14.11.9

--

//

ID Saspl_034778.T1

NAME Saspl_034778.T1

EC 1.14.11.9

--

//

ID Saspl_020877.T1

NAME Saspl_020877.T1

EC 1.14.11.9

--

//

ID Saspl_030605.T1

NAME Saspl_030605.T1

EC 1.14.11.9

//

ID Saspl_025458.T1

NAME Saspl_025458.T1

EC 1.14.13.88

--

//

ID Saspl_031512.T1

NAME Saspl_031512.T1

EC 1.14.13.88

--

//

ID Saspl_052235.T1

NAME Saspl_052235.T1

EC 1.14.13.88

--

//

ID Saspl_047043.T1

NAME Saspl_047043.T1

EC 1.14.13.88

--

//

ID Saspl_029526.T1

NAME Saspl_029526.T1

EC 1.14.13.88

--

//

ID Saspl_032397.T1

NAME Saspl_032397.T1

EC 1.14.13.88

--

//

ID Saspl_045189.T1

NAME Saspl_045189.T1

EC 1.14.13.88

--

//

ID Saspl_025681.T1

NAME Saspl_025681.T1

EC 1.14.13.88

--

//

ID Saspl_052980.T1

NAME Saspl_052980.T1

EC 1.14.13.88

--

//

ID Saspl_031511.T1

NAME Saspl_031511.T1

EC 1.14.13.88

--

//

ID Saspl_032396.T1

NAME Saspl_032396.T1

EC 1.14.13.88

--

//

ID Saspl_045187.T1

NAME Saspl_045187.T1

EC 1.14.13.88

--

//

ID Saspl_003098.T1

NAME Saspl_003098.T1

EC 1.14.13.88

--

//

ID Saspl_011836.T1

NAME Saspl_011836.T1

EC 1.14.13.88

--

//

ID Saspl_052981.T1

NAME Saspl_052981.T1

EC 1.14.13.88

--

//

ID Saspl_032394.T1

NAME Saspl_032394.T1

EC 1.14.13.88

//

ID Saspl_018661.T1

NAME Saspl_018661.T1

EC 1.1.1.208

--

METACYC RXN-5165

METACYC --MENTHOL-DEHYDROGENASE-RXN

METACYC RXN-5166

METACYC +-NEOMENTHOL-DEHYDROGENASE-RXN

EC 1.1.1.208

--

//

ID Saspl_040234.T1

NAME Saspl_040234.T1

EC 1.1.1.208

--

METACYC RXN-5165

METACYC --MENTHOL-DEHYDROGENASE-RXN

METACYC RXN-5166

METACYC +-NEOMENTHOL-DEHYDROGENASE-RXN

EC 1.1.1.208

--

//

ID Saspl_027322.T1

NAME Saspl_027322.T1

EC 1.1.1.208

--

METACYC RXN-5165

METACYC --MENTHOL-DEHYDROGENASE-RXN

METACYC RXN-5166

METACYC +-NEOMENTHOL-DEHYDROGENASE-RXN

EC 1.1.1.208

--

METACYC RXN-5165

METACYC --MENTHOL-DEHYDROGENASE-RXN

METACYC RXN-5166

METACYC +-NEOMENTHOL-DEHYDROGENASE-RXN

EC 1.1.1.208

--

//

ID Saspl_027200.T1

NAME Saspl_027200.T1

EC 1.1.1.208

NAME Saspl_027577.T1

METACYC ISOPIPERITENOL-DEHYDROGENASE-RXN

METACYC CARVEOL-DEHYDROGENASE-RXN

EC 1.1.1.223

--

NAME Saspl_027581.T1

METACYC ISOPIPERITENOL-DEHYDROGENASE-RXN

METACYC CARVEOL-DEHYDROGENASE-RXN

EC 1.1.1.223

--

NAME Saspl_027565.T1

METACYC ISOPIPERITENOL-DEHYDROGENASE-RXN

METACYC CARVEOL-DEHYDROGENASE-RXN

EC 1.1.1.223

--

NAME Saspl_027579.T1

METACYC ISOPIPERITENOL-DEHYDROGENASE-RXN

METACYC CARVEOL-DEHYDROGENASE-RXN

EC 1.1.1.223

--

NAME Saspl_027573.T1

METACYC ISOPIPERITENOL-DEHYDROGENASE-RXN

METACYC CARVEOL-DEHYDROGENASE-RXN

EC 1.1.1.223

--

NAME Saspl_052938.T1

METACYC ISOPIPERITENOL-DEHYDROGENASE-RXN

METACYC CARVEOL-DEHYDROGENASE-RXN

EC 1.1.1.223

--

NAME Saspl_027575.T1

METACYC ISOPIPERITENOL-DEHYDROGENASE-RXN

METACYC CARVEOL-DEHYDROGENASE-RXN

EC 1.1.1.223

--

NAME Saspl_017051.T1

METACYC ISOPIPERITENOL-DEHYDROGENASE-RXN

METACYC CARVEOL-DEHYDROGENASE-RXN

EC 1.1.1.223

--

NAME Saspl_027582.T1

METACYC ISOPIPERITENOL-DEHYDROGENASE-RXN

METACYC CARVEOL-DEHYDROGENASE-RXN

EC 1.1.1.223

--

NAME Saspl_052937.T1

METACYC ISOPIPERITENOL-DEHYDROGENASE-RXN

METACYC CARVEOL-DEHYDROGENASE-RXN

EC 1.1.1.223

--

NAME Saspl_027834.T1

METACYC ISOPIPERITENOL-DEHYDROGENASE-RXN

METACYC CARVEOL-DEHYDROGENASE-RXN

EC 1.1.1.223

--

NAME Saspl_027580.T1

METACYC ISOPIPERITENOL-DEHYDROGENASE-RXN

METACYC CARVEOL-DEHYDROGENASE-RXN

EC 1.1.1.223

--

NAME Saspl_017061.T1

METACYC ISOPIPERITENOL-DEHYDROGENASE-RXN

METACYC CARVEOL-DEHYDROGENASE-RXN

EC 1.1.1.223

--

NAME Saspl_027566.T1

METACYC ISOPIPERITENOL-DEHYDROGENASE-RXN

METACYC CARVEOL-DEHYDROGENASE-RXN

EC 1.1.1.223

--

NAME Saspl_017062.T1

METACYC ISOPIPERITENOL-DEHYDROGENASE-RXN

METACYC CARVEOL-DEHYDROGENASE-RXN

EC 1.1.1.223

--

NAME Saspl_027571.T1

METACYC ISOPIPERITENOL-DEHYDROGENASE-RXN

METACYC CARVEOL-DEHYDROGENASE-RXN

EC 1.1.1.223

--

NAME Saspl_052939.T1

METACYC ISOPIPERITENOL-DEHYDROGENASE-RXN

METACYC CARVEOL-DEHYDROGENASE-RXN

EC 1.1.1.223

//

ID Saspl_035692.T1

NAME Saspl_035692.T1

METACYC 1.14.13.104-RXN

EC 1.14.13.104

//

ID Saspl_017054.T1

NAME Saspl_017054.T1

EC 1.14.13.47

--

//

ID Saspl_027838.T1

NAME Saspl_027838.T1

EC 1.14.13.47

--

ID Saspl_028255.T1

NAME Saspl_028255.T1

METACYC --LIMONENE-3-MONOOXYGENASE-RXN

EC 1.14.13.47

--

ID Saspl_017797.T1

NAME Saspl_017797.T1

METACYC --LIMONENE-3-MONOOXYGENASE-RXN

EC 1.14.13.47

--

//

ID Saspl_027839.T1

NAME Saspl_027839.T1

EC 1.14.13.47

--

ID Saspl_049292.T1

NAME Saspl_049292.T1

METACYC --LIMONENE-3-MONOOXYGENASE-RXN

EC 1.14.13.47

--

ID Saspl_009142.T1

NAME Saspl_009142.T1

METACYC --LIMONENE-3-MONOOXYGENASE-RXN

EC 1.14.13.47

--

//

ID Saspl_029000.T1

NAME Saspl_029000.T1

EC 1.14.13.47

--

ID Saspl_017055.T1

NAME Saspl_017055.T1

METACYC --LIMONENE-3-MONOOXYGENASE-RXN

EC 1.14.13.47

--

//

ID Saspl_027836.T1

NAME Saspl_027836.T1

EC 1.14.13.47

--

ID Saspl_009141.T1

NAME Saspl_009141.T1

METACYC --LIMONENE-3-MONOOXYGENASE-RXN

EC 1.14.13.47

//

ID Saspl_039874.T1

NAME Saspl_039874.T1

EC 2.3.1.74

--

//

ID Saspl_039872.T1

NAME Saspl_039872.T1

EC 2.3.1.74

--

//

ID Saspl_016605.T1

NAME Saspl_016605.T1

EC 2.3.1.74

--

//

ID Saspl_010516.T1

NAME Saspl_010516.T1

EC 2.3.1.74

--

//

ID Saspl_004527.T1

NAME Saspl_004527.T1

EC 2.3.1.74

--

//

ID Saspl_048124.T1

NAME Saspl_048124.T1

EC 2.3.1.74

--

//

ID Saspl_016604.T1

NAME Saspl_016604.T1

EC 2.3.1.74

--

//

ID Saspl_039871.T1

NAME Saspl_039871.T1

EC 2.3.1.74

--

//

ID Saspl_039873.T1

NAME Saspl_039873.T1

EC 2.3.1.74

--

//

ID Saspl_000636.T1

NAME Saspl_000636.T1

EC 2.3.1.74

//

ID Saspl_004313.T1

NAME Saspl_004313.T1

METACYC 4.2.3.16-RXN

//

ID Saspl_047571.T1

NAME Saspl_047571.T1

EC 5.5.1.6

--

//

ID Saspl_046151.T1

NAME Saspl_046151.T1

EC 5.5.1.6

--

//

ID Saspl_042638.T1

NAME Saspl_042638.T1

EC 5.5.1.6

--

//

ID Saspl_043806.T1

NAME Saspl_043806.T1

EC 5.5.1.6

--

//

ID Saspl_025956.T1

NAME Saspl_025956.T1

EC 5.5.1.6

--

//

ID Saspl_038438.T1

NAME Saspl_038438.T1

EC 5.5.1.6

--

//

ID Saspl_038198.T1

NAME Saspl_038198.T1

EC 5.5.1.6

--

//

ID Saspl_020531.T1

NAME Saspl_020531.T1

EC 5.5.1.6
