# Supplementary material for: High-quality assembly of the reference genome for scarlet sage, Salvia splendens, an economically important ornamental plant
Source: Gigascience. 2018 Jun 19;7(7):giy068. doi: 10.1093/gigascience/giy068 (PMC6030905; doi:10.1093/gigascience/giy068)
Supplement: Additional Files [file giy068_supplemental_files.zip › Supplementary_File_2.docx]

1. **scaffold1 - Cluster 1 – Polyketide**

**
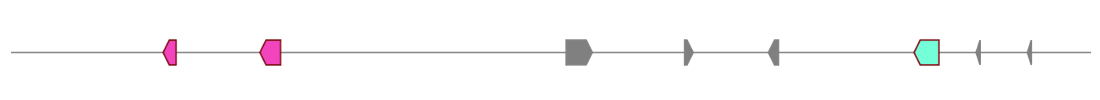
**

**
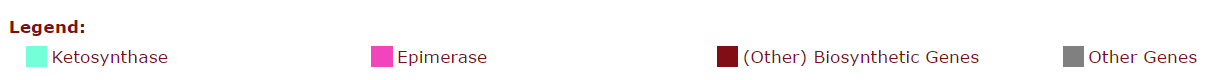
**

**
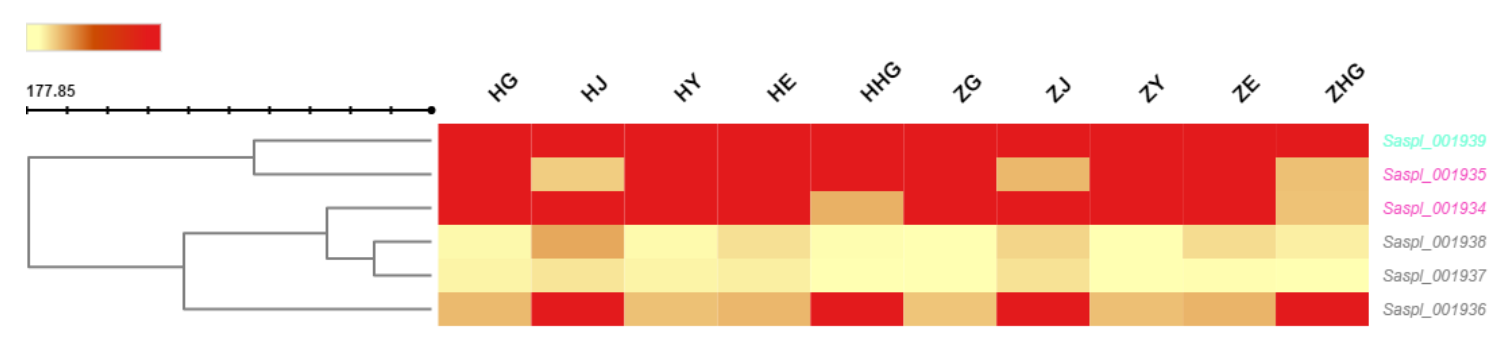
**

1. **scaffold10 - Cluster 2 – Putative**

**
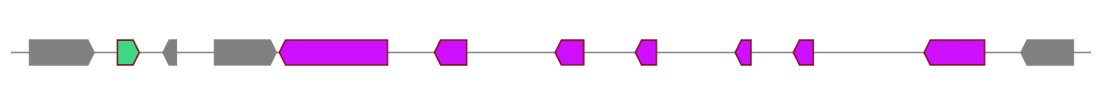
**

**
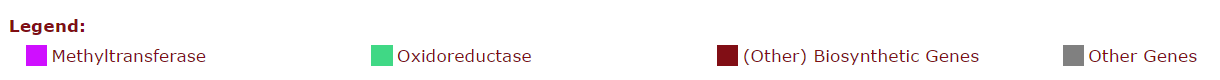
**

**
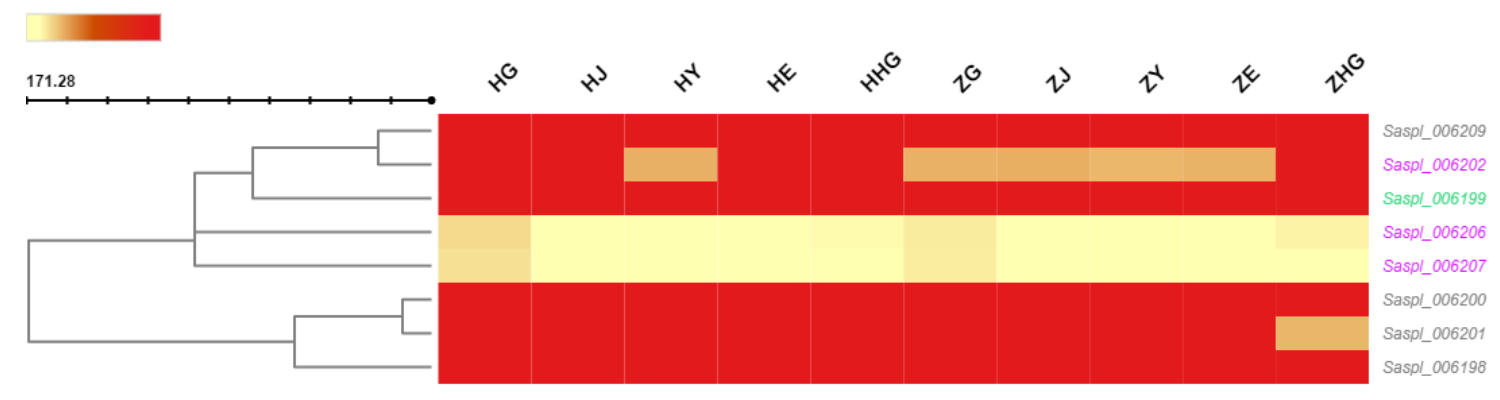
**

1. **scaffold102 - Cluster 3 – Putative**

**
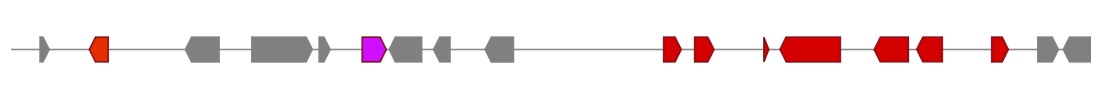
**

**
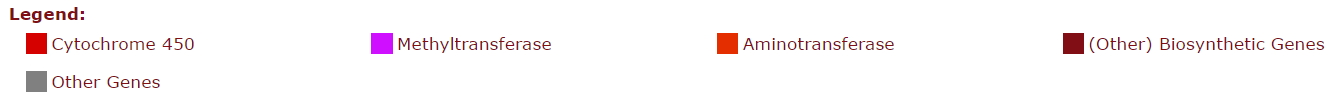
**

**
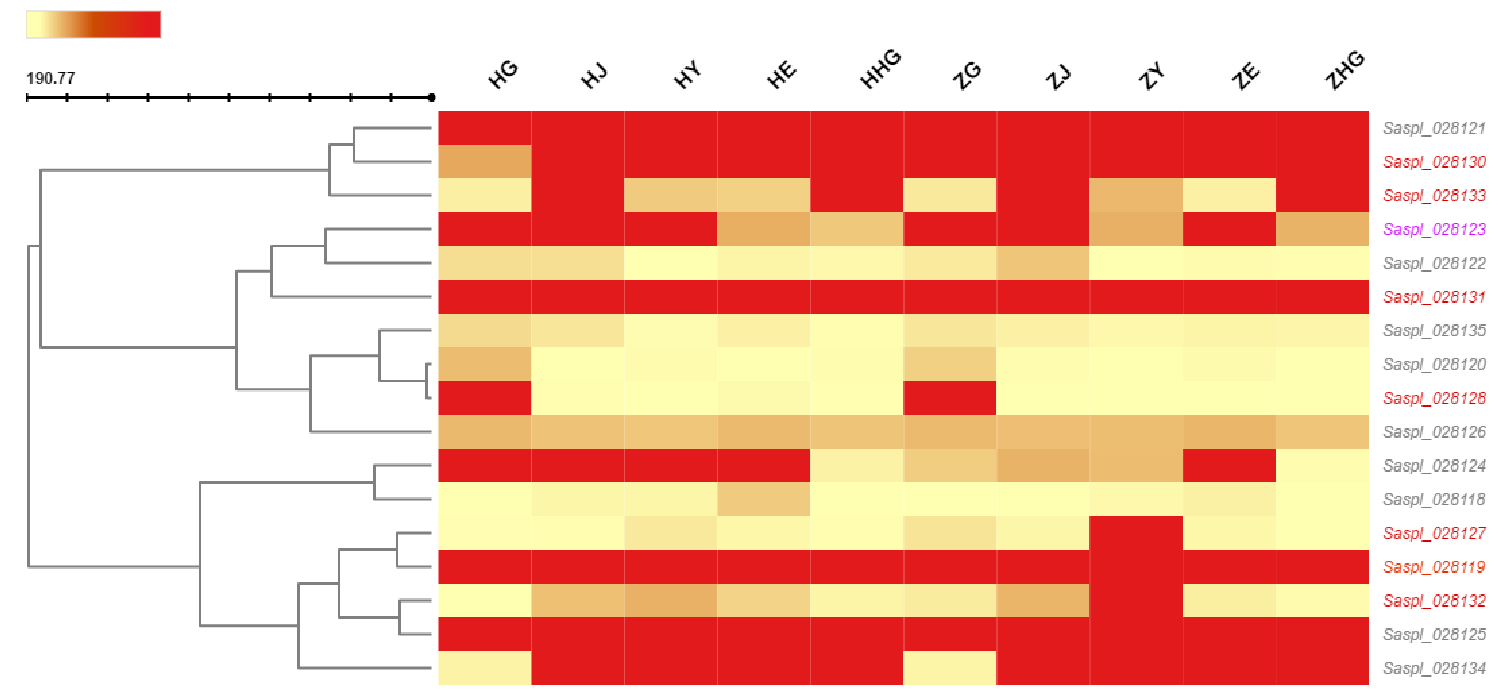
**

1. **scaffold102 - Cluster 4 – Saccharide**

**
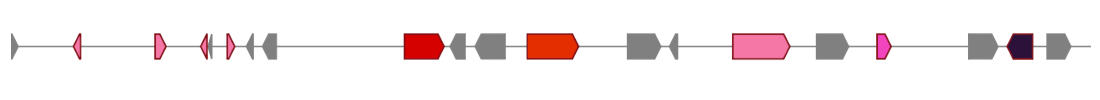
**

**
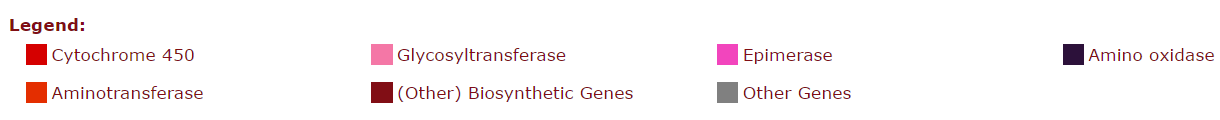
**

**
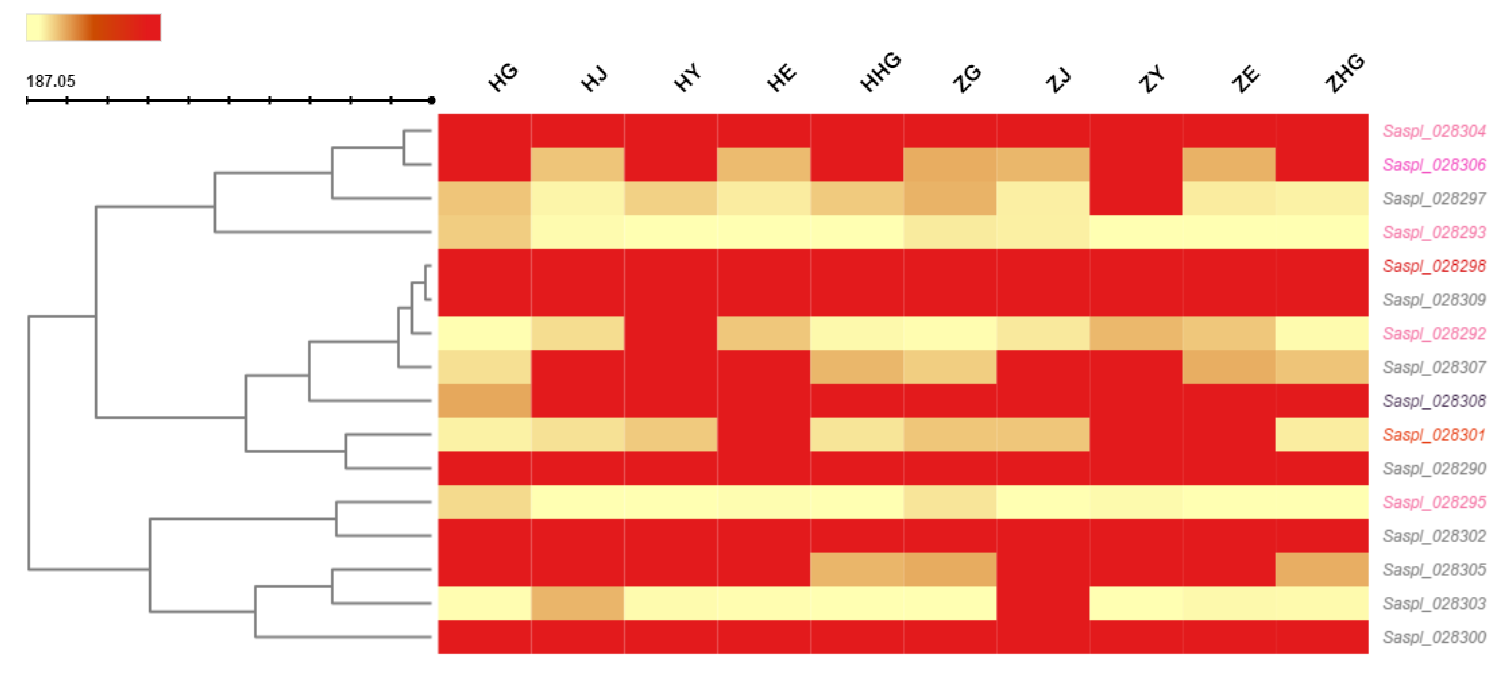
**

1. **scaffold103 - Cluster 5 – Putative**

**
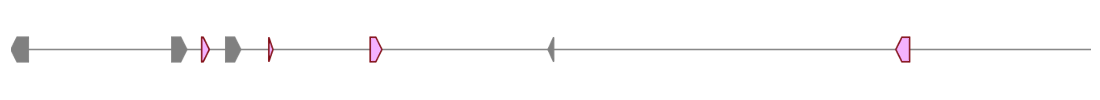
**

**
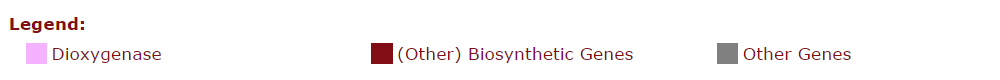
**

**
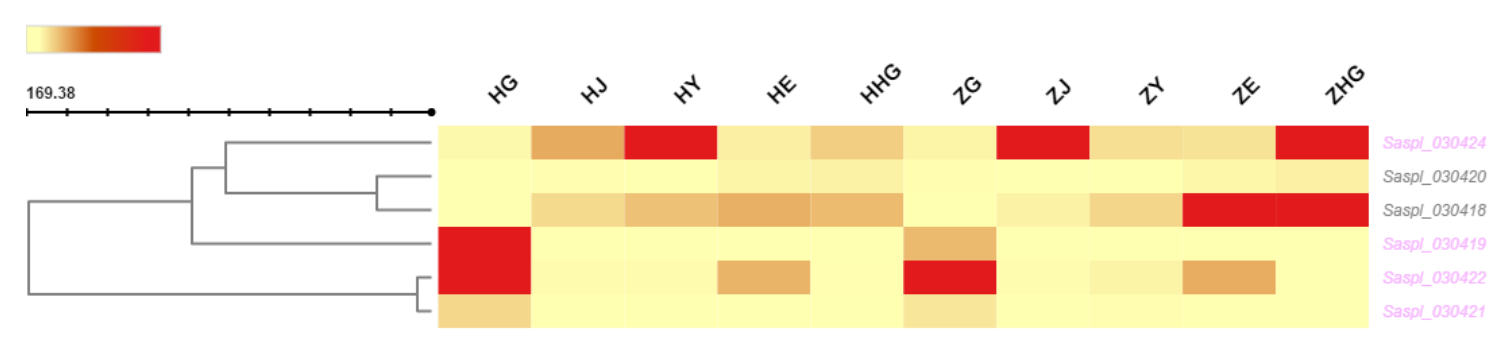
**

1. **scaffold109 - Cluster 6 – Saccharide**

**
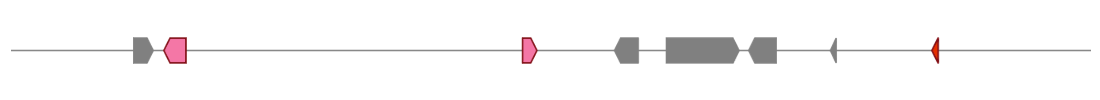
**

**
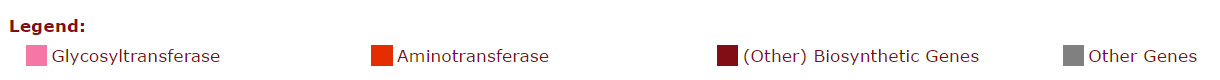
**

**
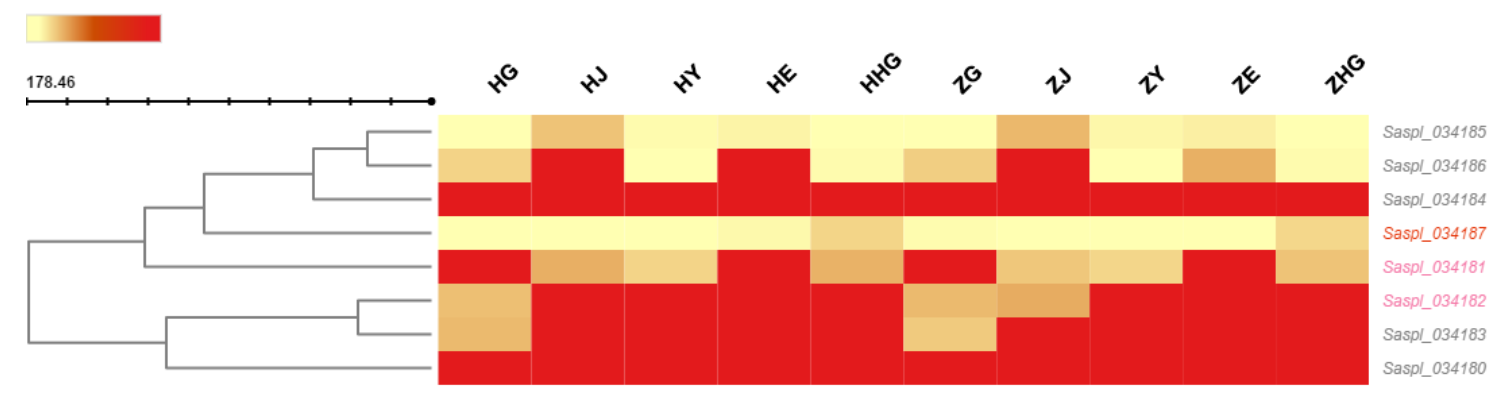
**

1. **scaffold114 - Cluster 7 – Alkaloid**

**
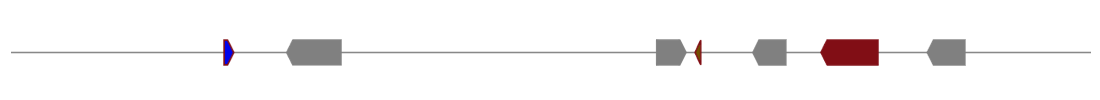
**

**
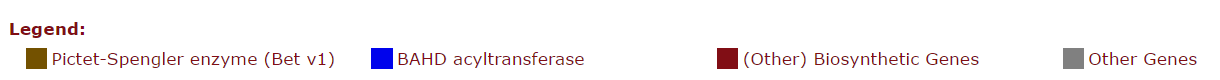
**


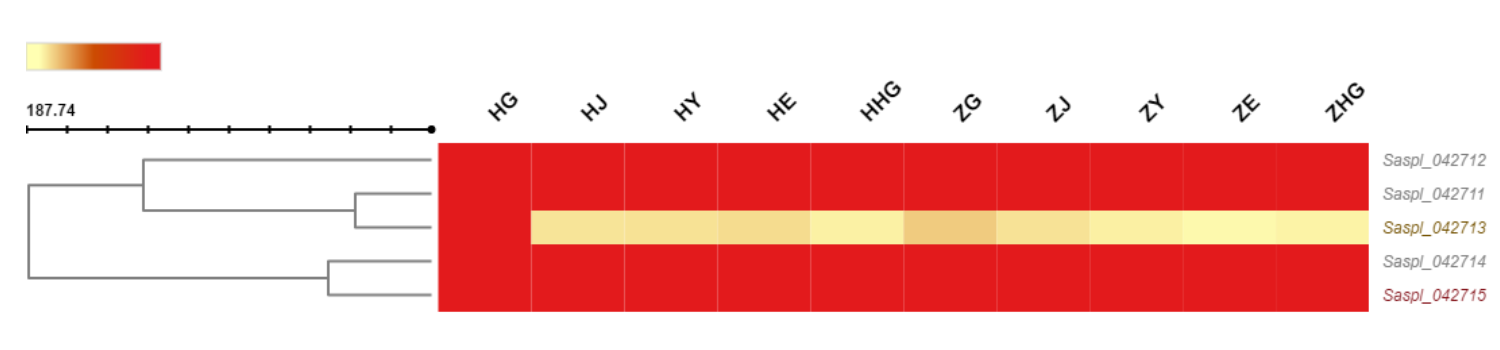


1. **scaffold12 - Cluster 8 – Saccharide**

**
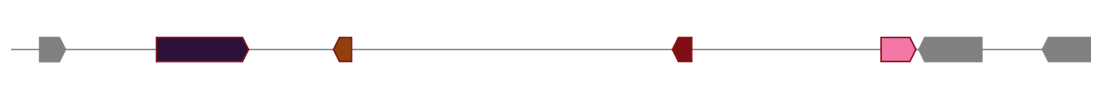
**

**
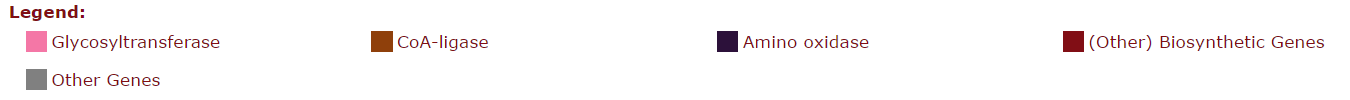
**

**
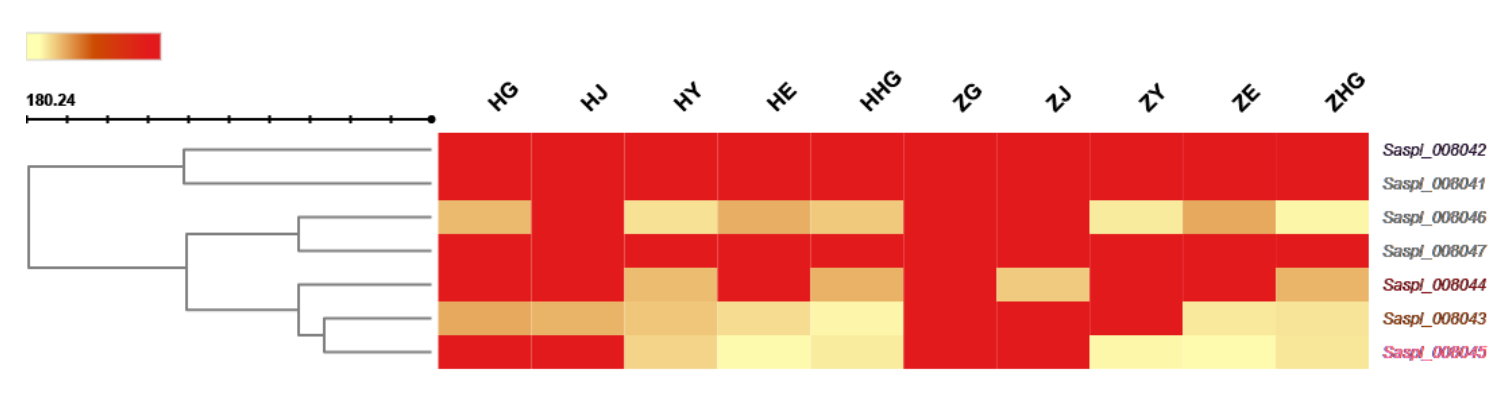
**

1. **scaffold123 - Cluster 9 – Saccharide**

**
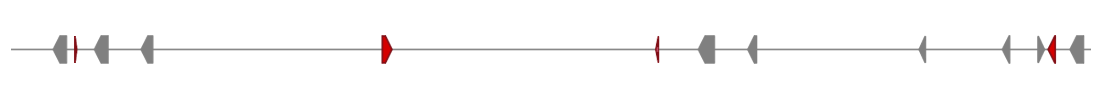
**

**
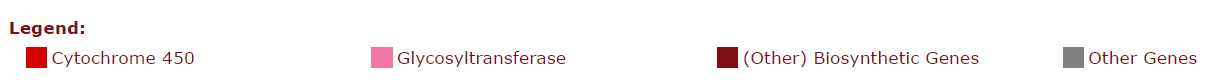
**

**
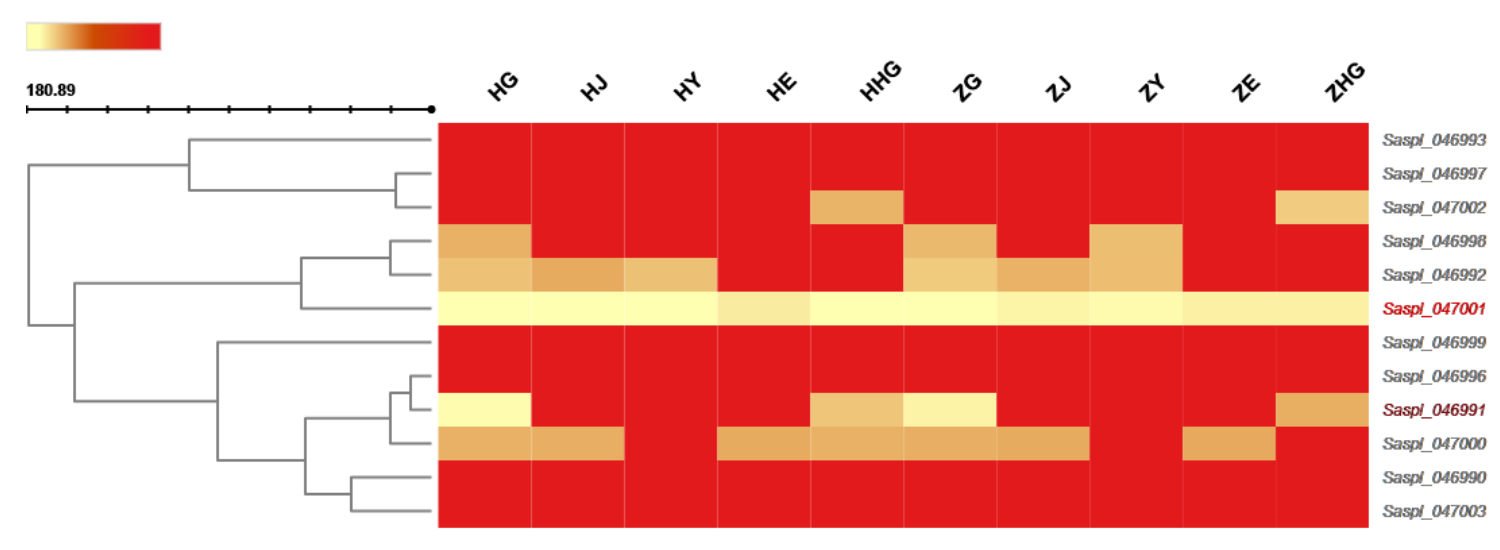
**

1. **scaffold124 - Cluster 10 – Saccharide**

**
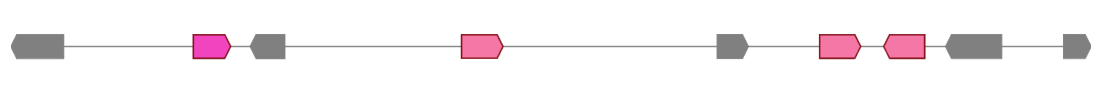
**

**
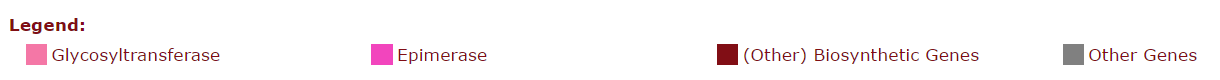
**

**
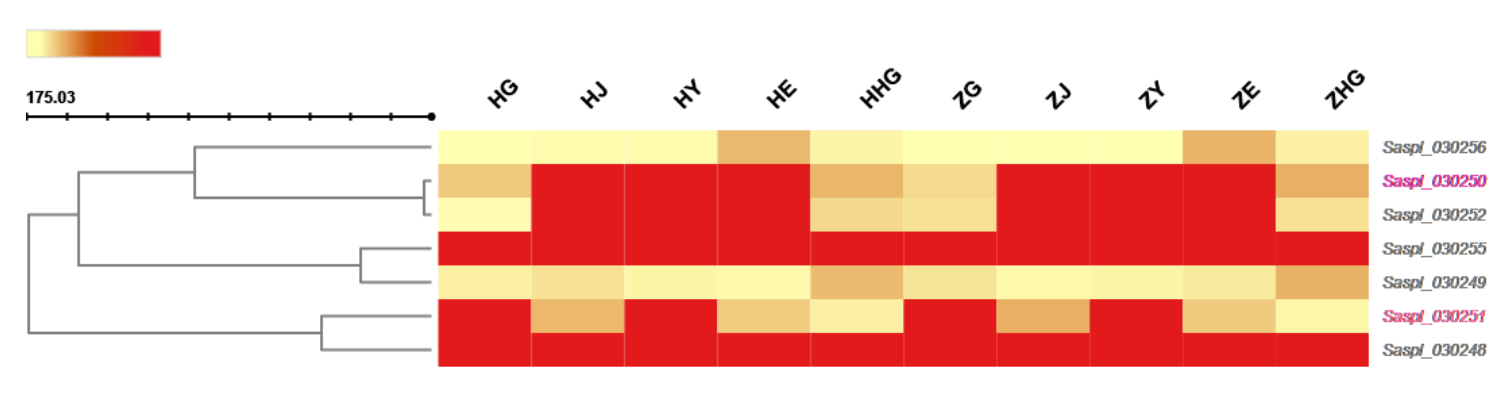
**

1. **scaffold124 - Cluster 11 - Saccharide-terpene**

**
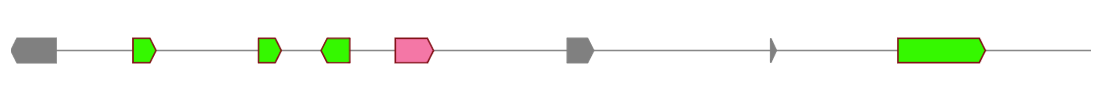
**

**
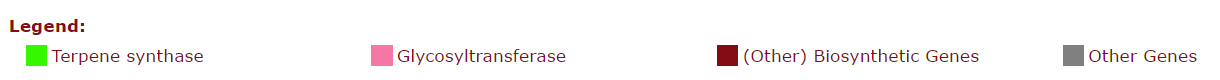
**

**
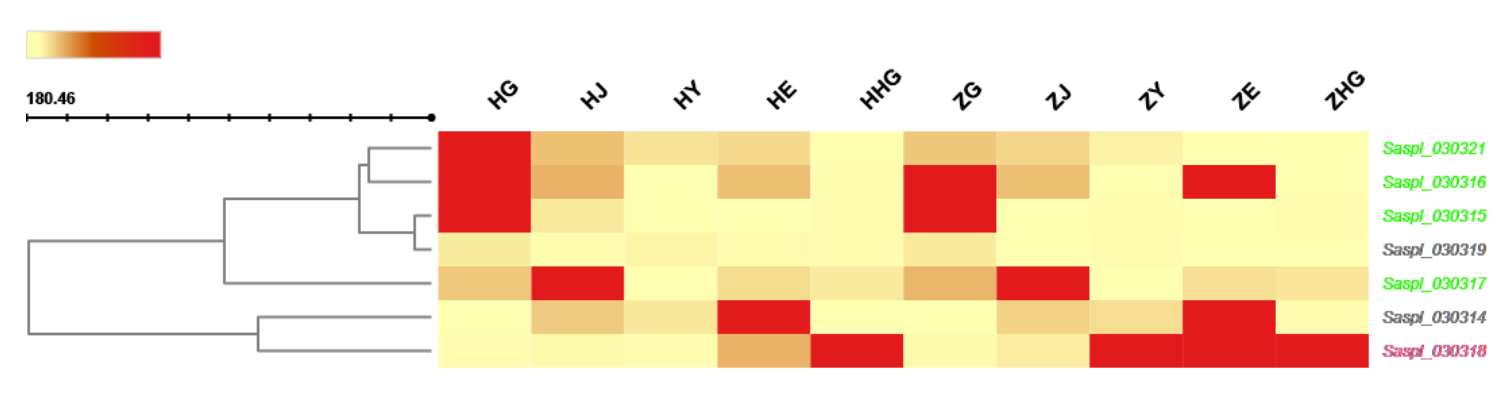
**

1. **scaffold124 - Cluster 12 - Saccharide-terpene-alkaloid**

**
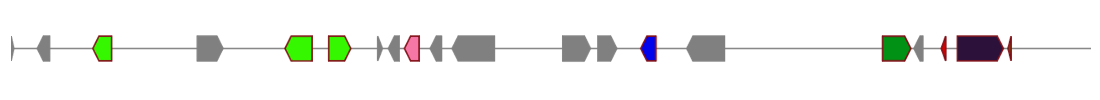
**

**
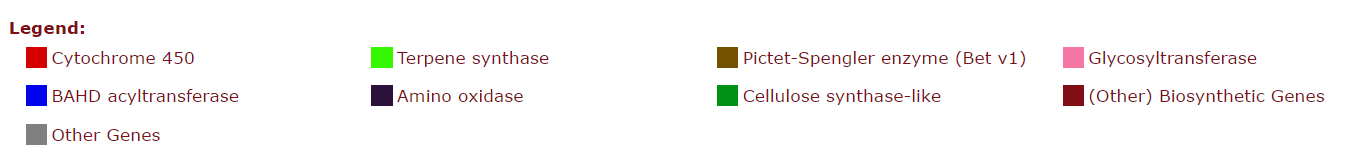
**

**
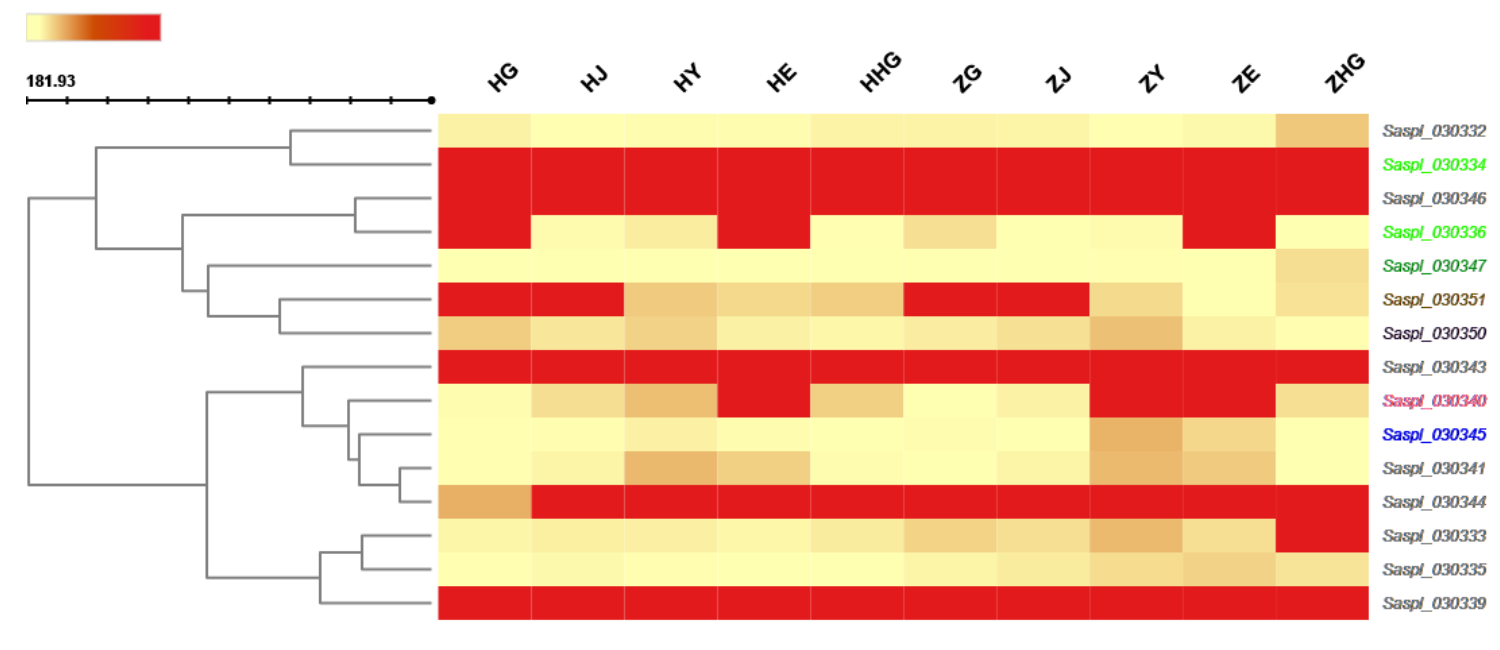
**

1. **scaffold129 - Cluster 13 – Terpene**

**
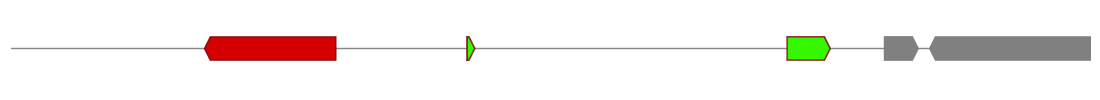
**

**
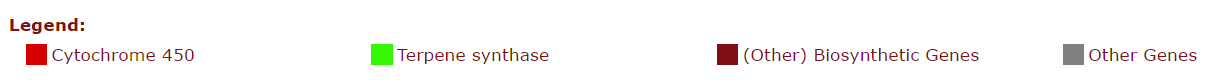
**

**
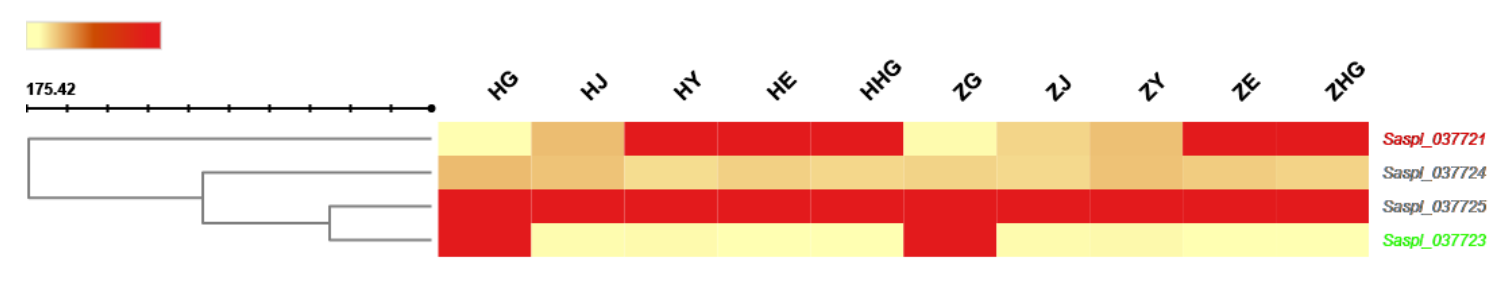
**

1. **scaffold13 - Cluster 14 – Saccharide**

**
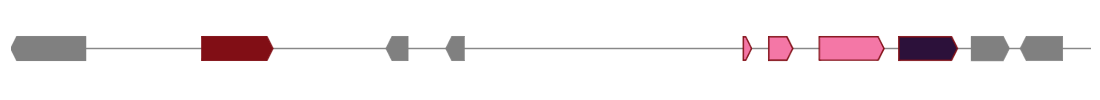
**

**
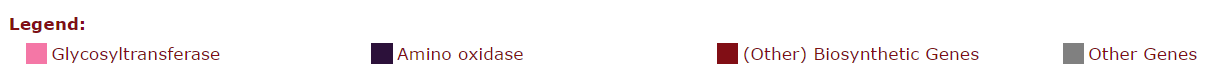
**

**
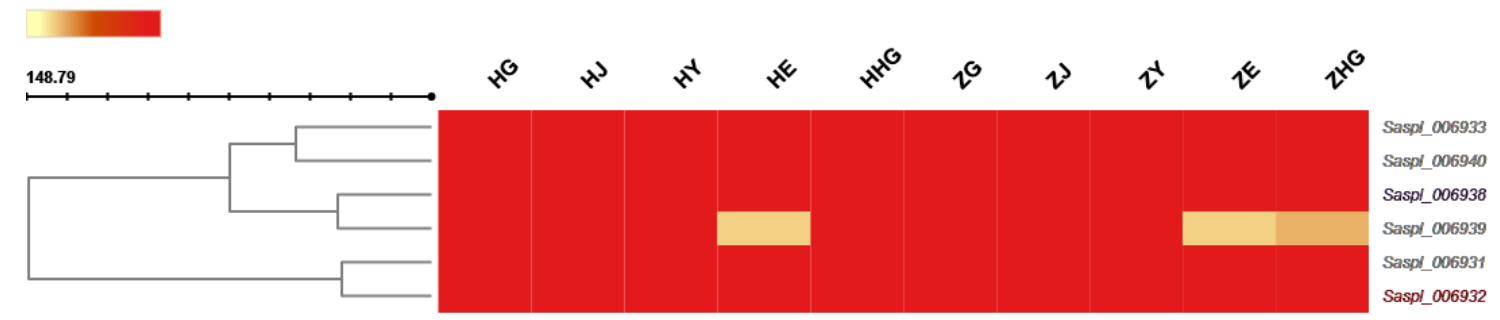
**

1. **scaffold131 - Cluster 15 – Alkaloid**

**
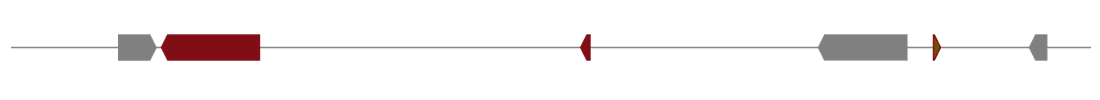
**

**
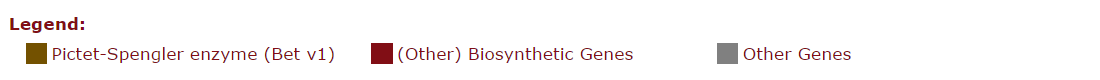
**

**
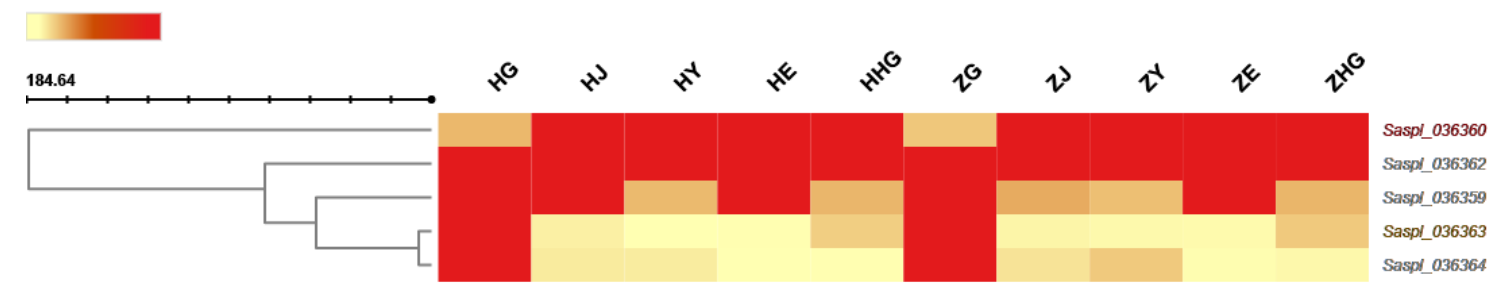
**

1. **scaffold148 - Cluster 16 – Saccharide**

**
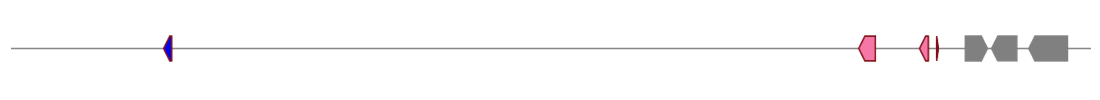
**

**
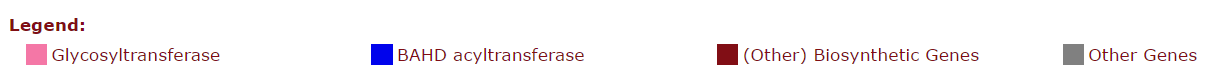
**

**
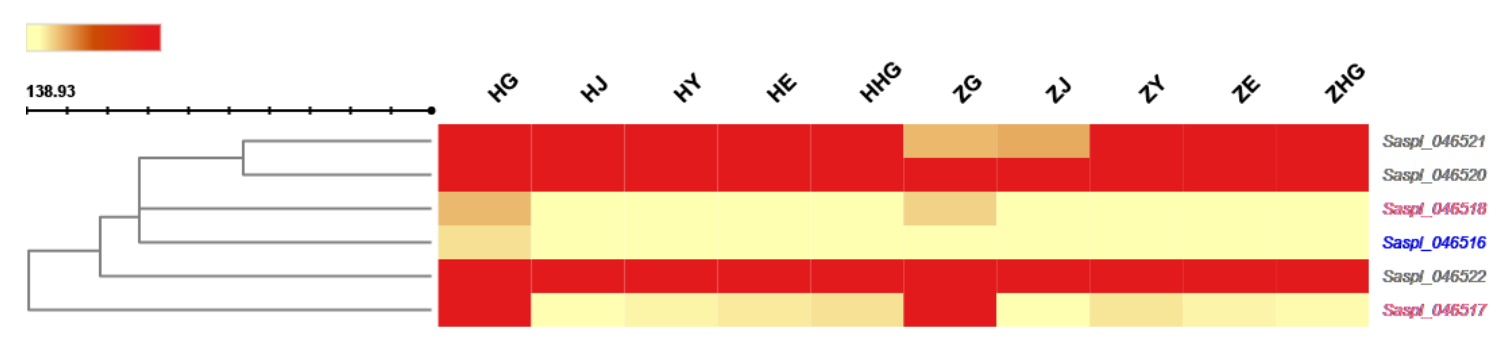
**

1. **scaffold148 - Cluster 17 – Terpene**

**
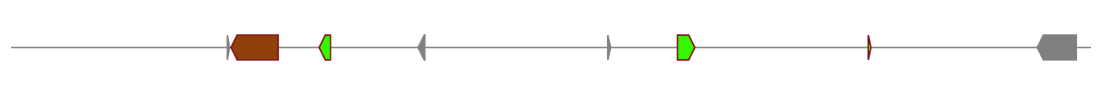
**

**
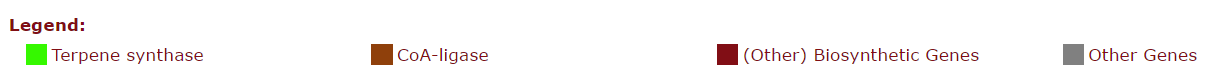
**

**
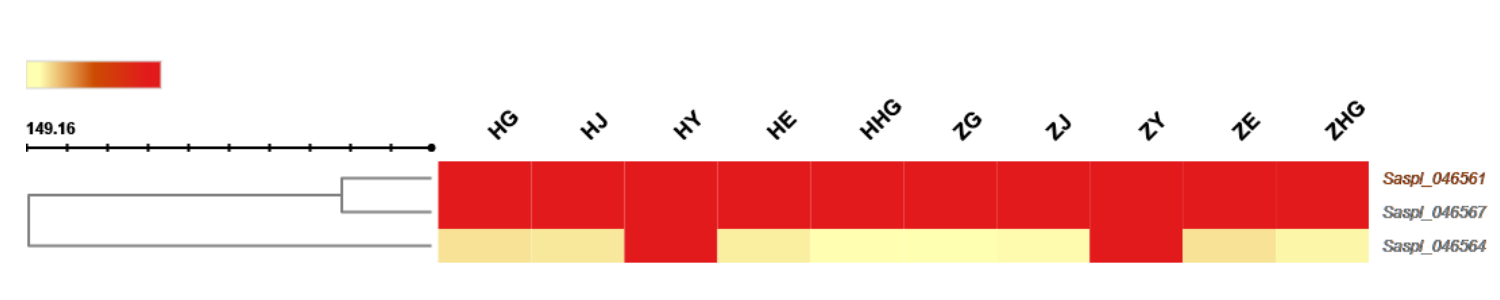
**

1. **scaffold157 - Cluster 18 – Terpene**

**
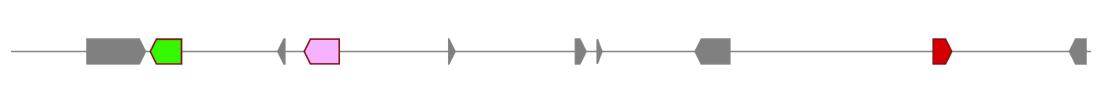
**

**
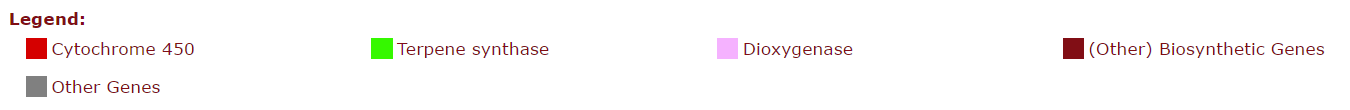
**

**
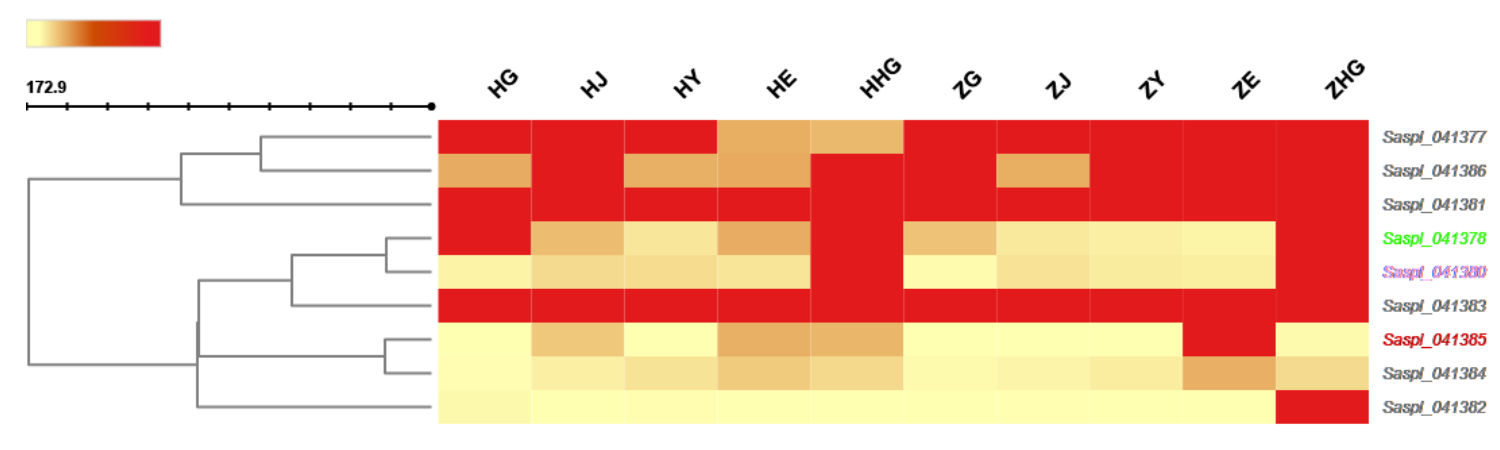
**

1. **scaffold159 - Cluster 19 - Terpene-alkaloid**

**
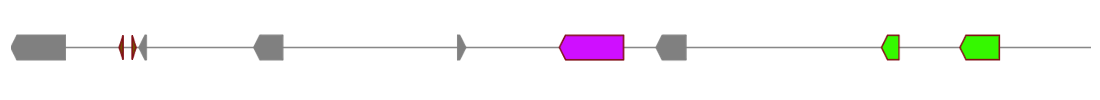
**

**
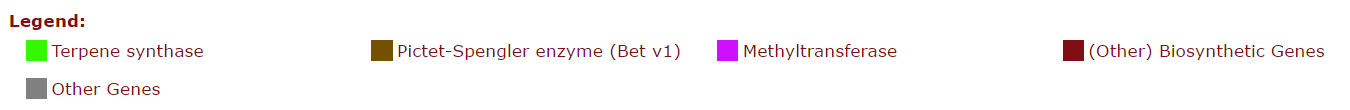
**

**
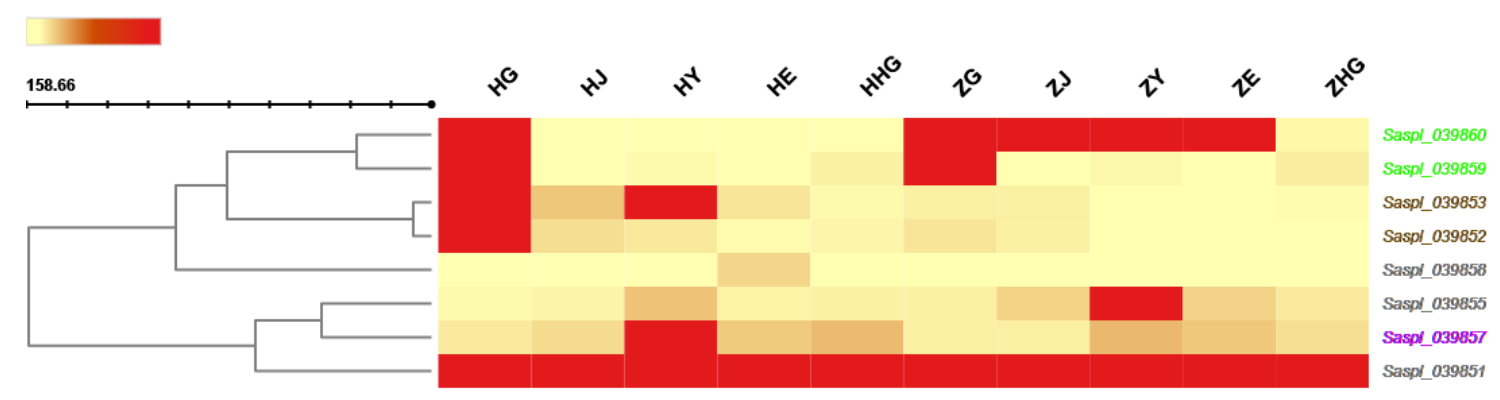
**

1. **scaffold159 - Cluster 20 - Terpene-polyketide**

**
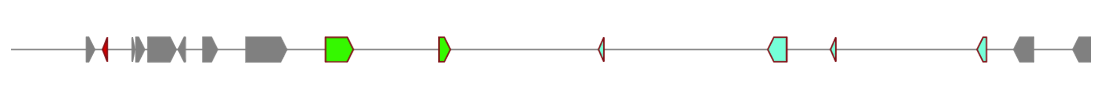
**

**
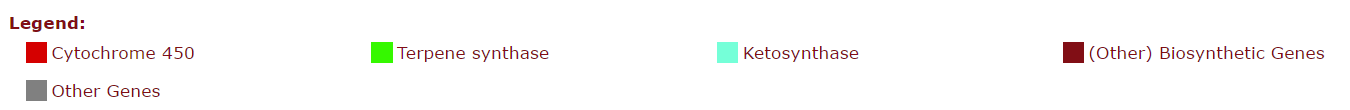
**

**
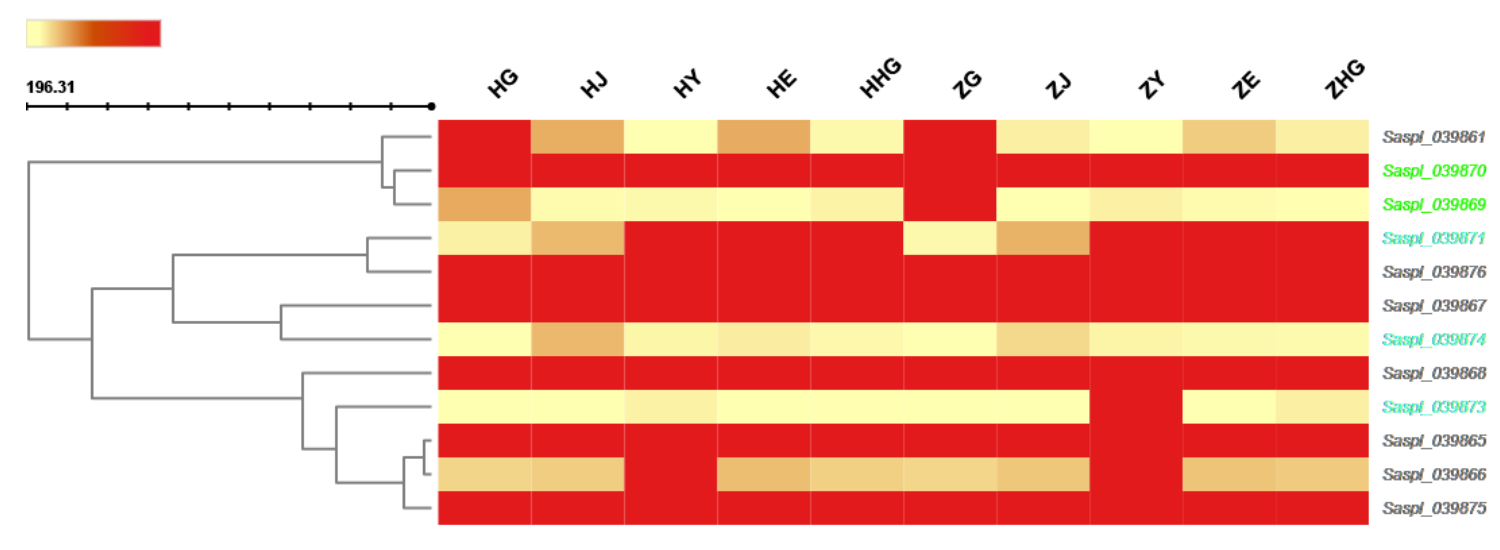
**

1. **scaffold173 - Cluster 21 – Putative**

**
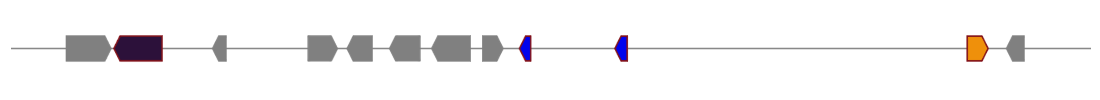
**

**
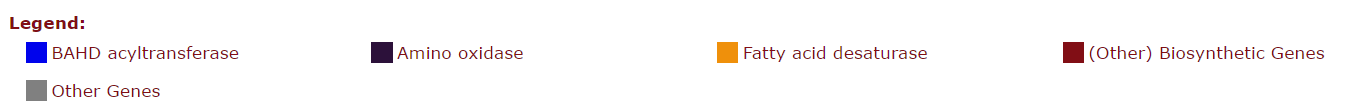
**

**
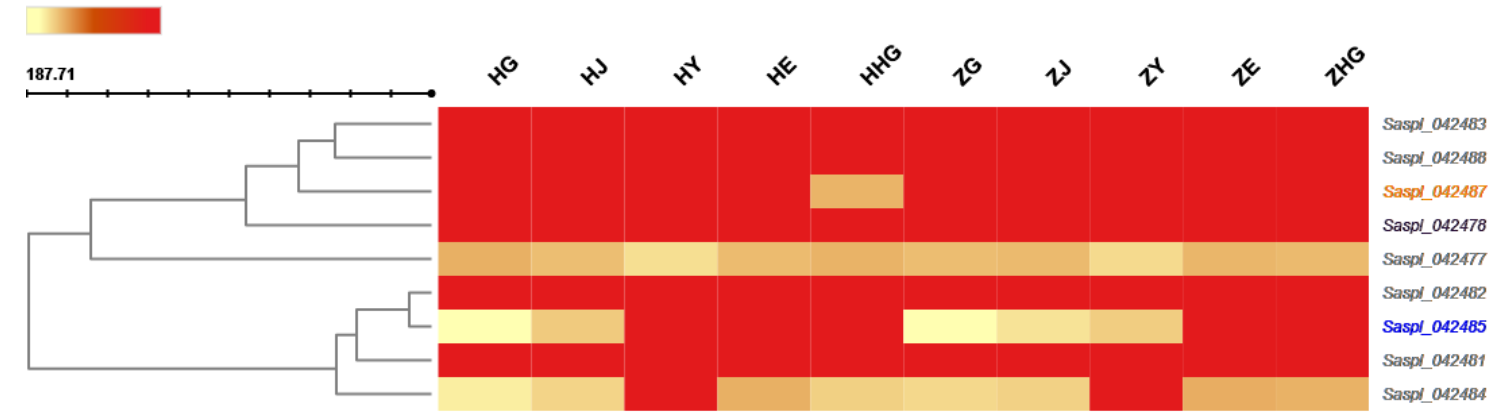
**

1. **scaffold174 - Cluster 22 – Terpene**

**
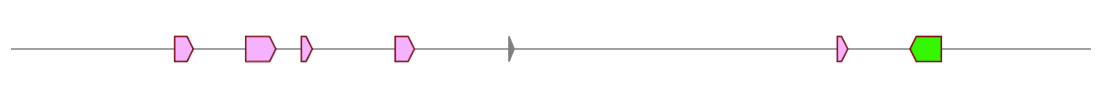
**

**
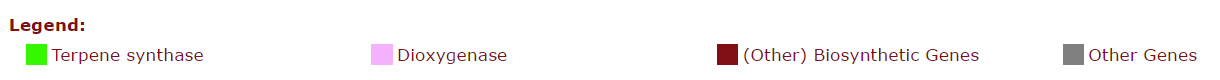
**

**
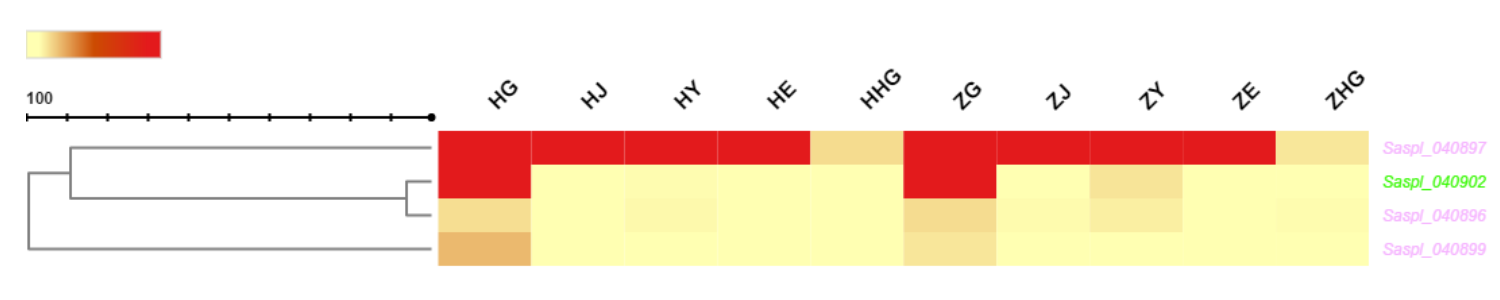
**

1. **scaffold19 - Cluster 23 – Lignan**

**
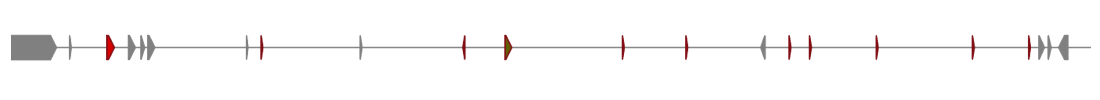
**

**
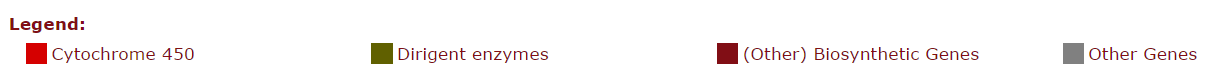
**

**
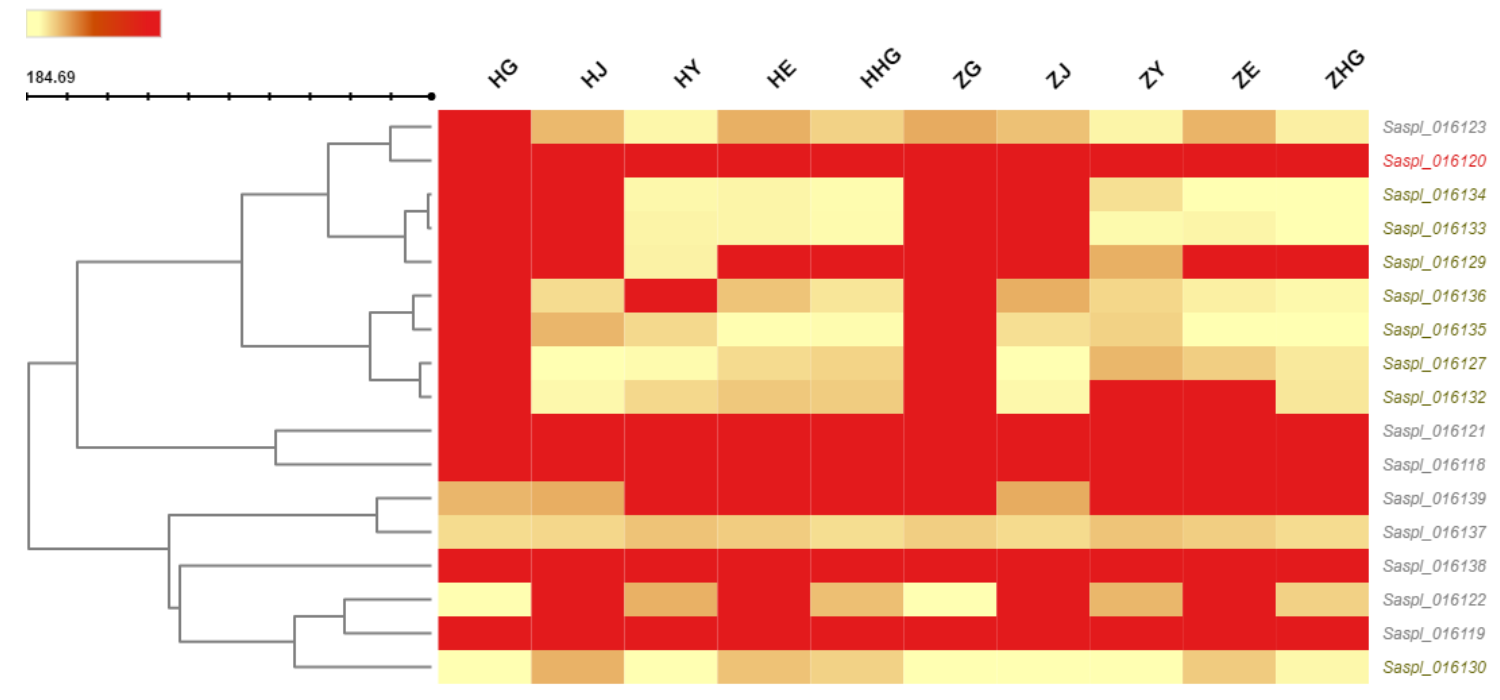
**

1. **scaffold198 - Cluster 24 – Saccharide**

**
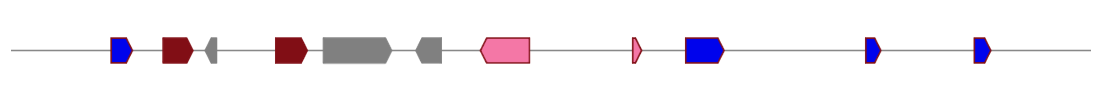
**

**
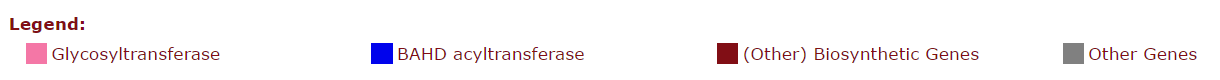
**

**
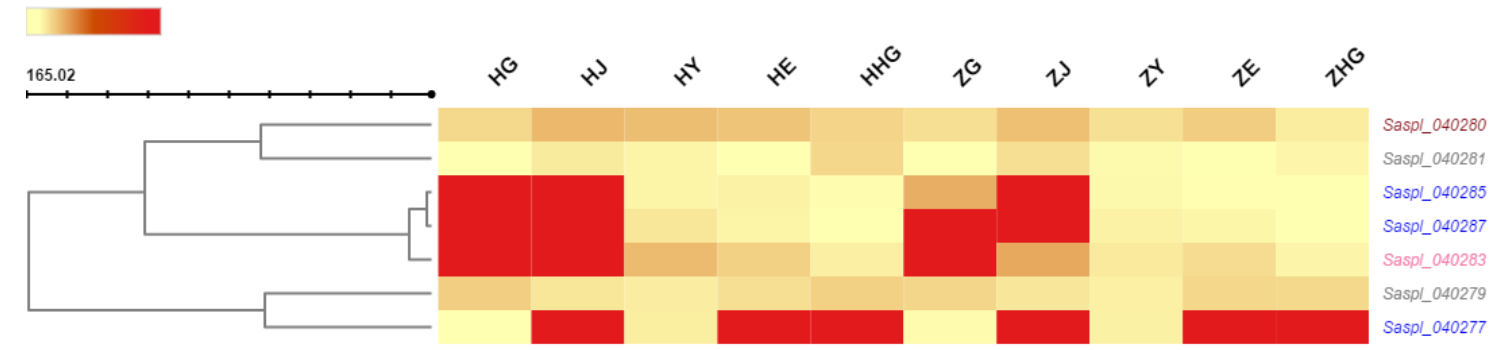
**

1. **scaffold2 - Cluster 25 – Putative**

**
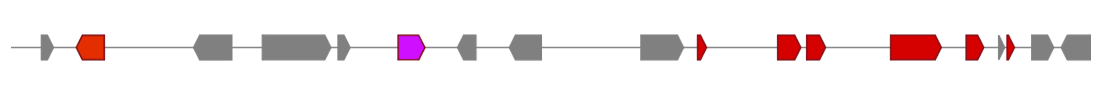
**

**
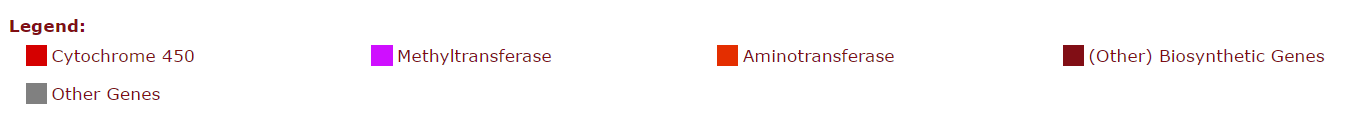
**

**
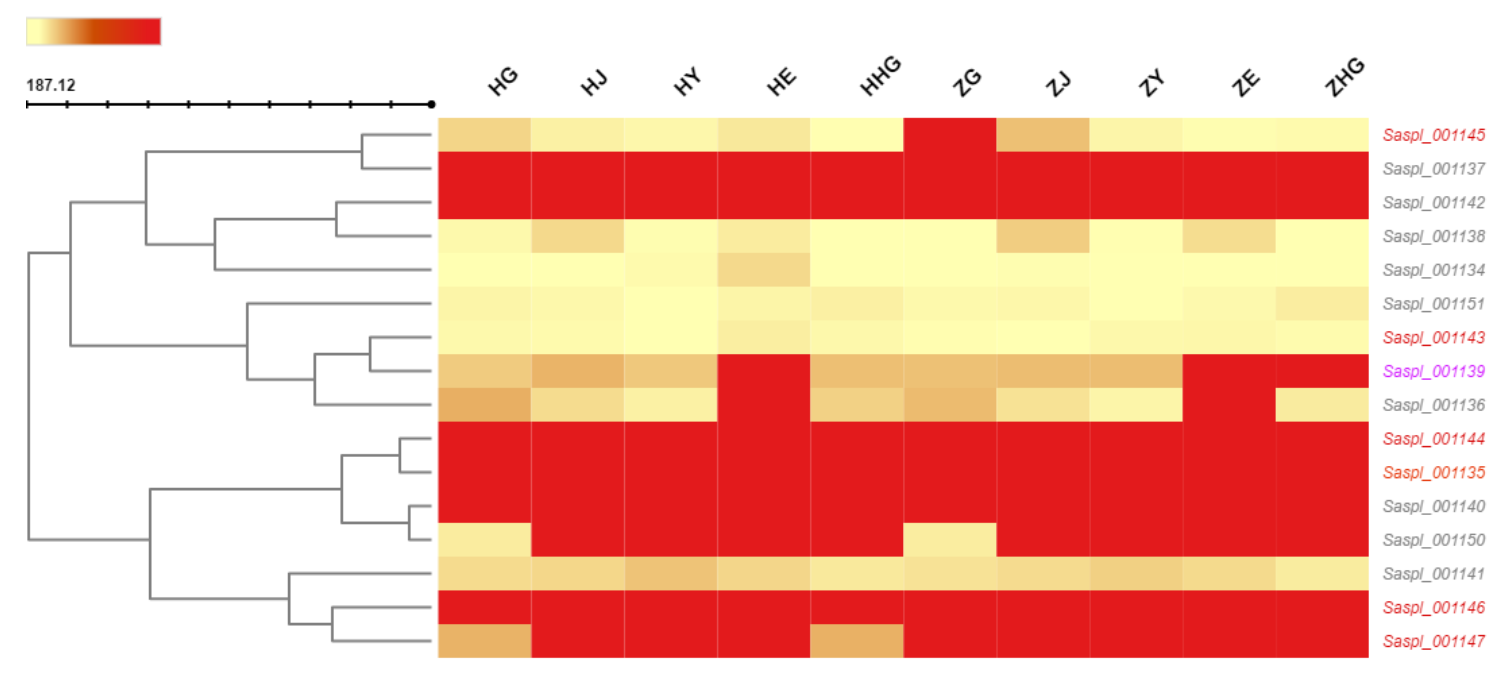
**

1. **scaffold20 - Cluster 26 - Terpene-lignan-polyketide**

**
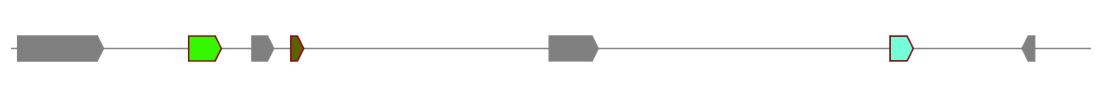
**

**
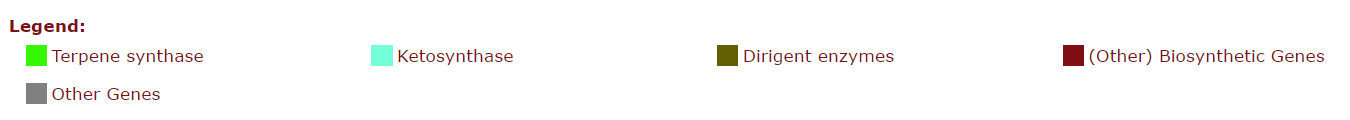
**

**
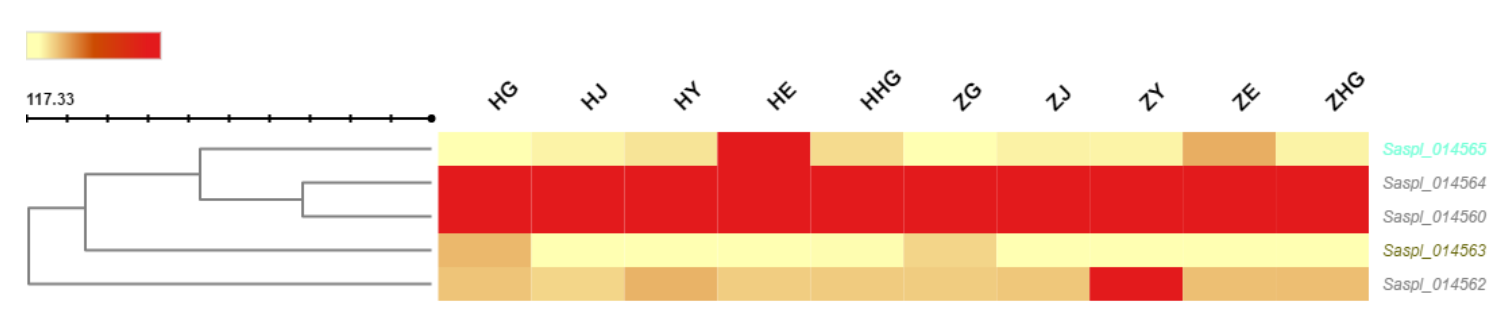
**

1. **scaffold21 - Cluster 27 – Lignan**

**
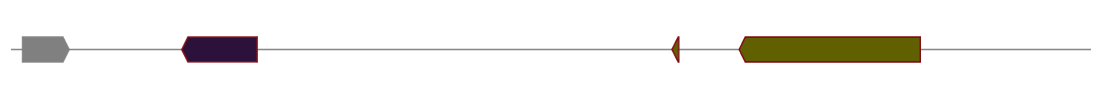
**

**
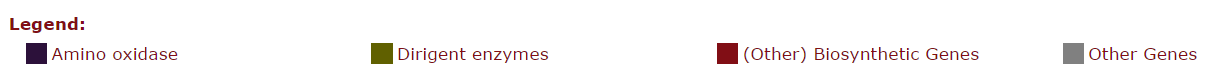
**

**
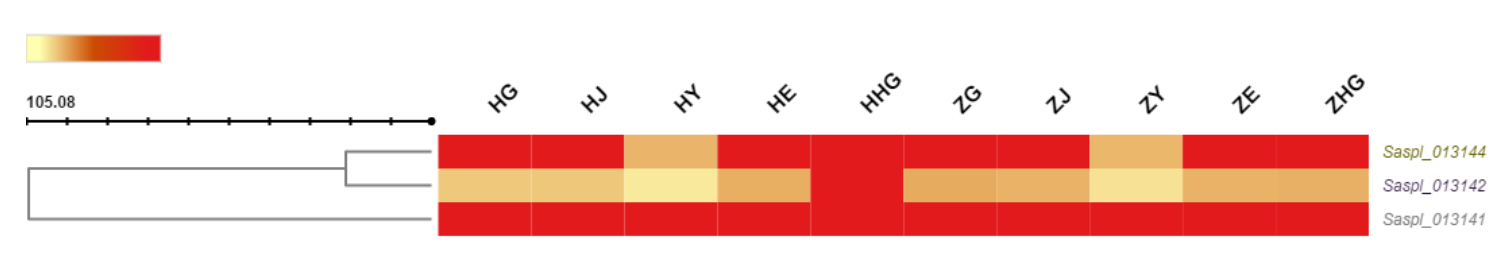
**

1. **scaffold21 - Cluster 28 – Putative**

**
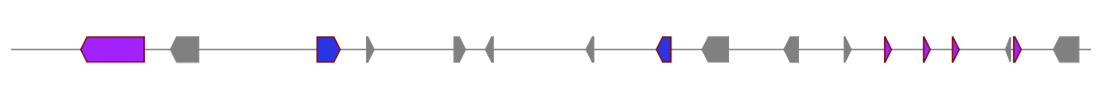
**

**
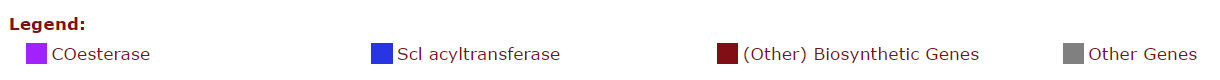
**

**
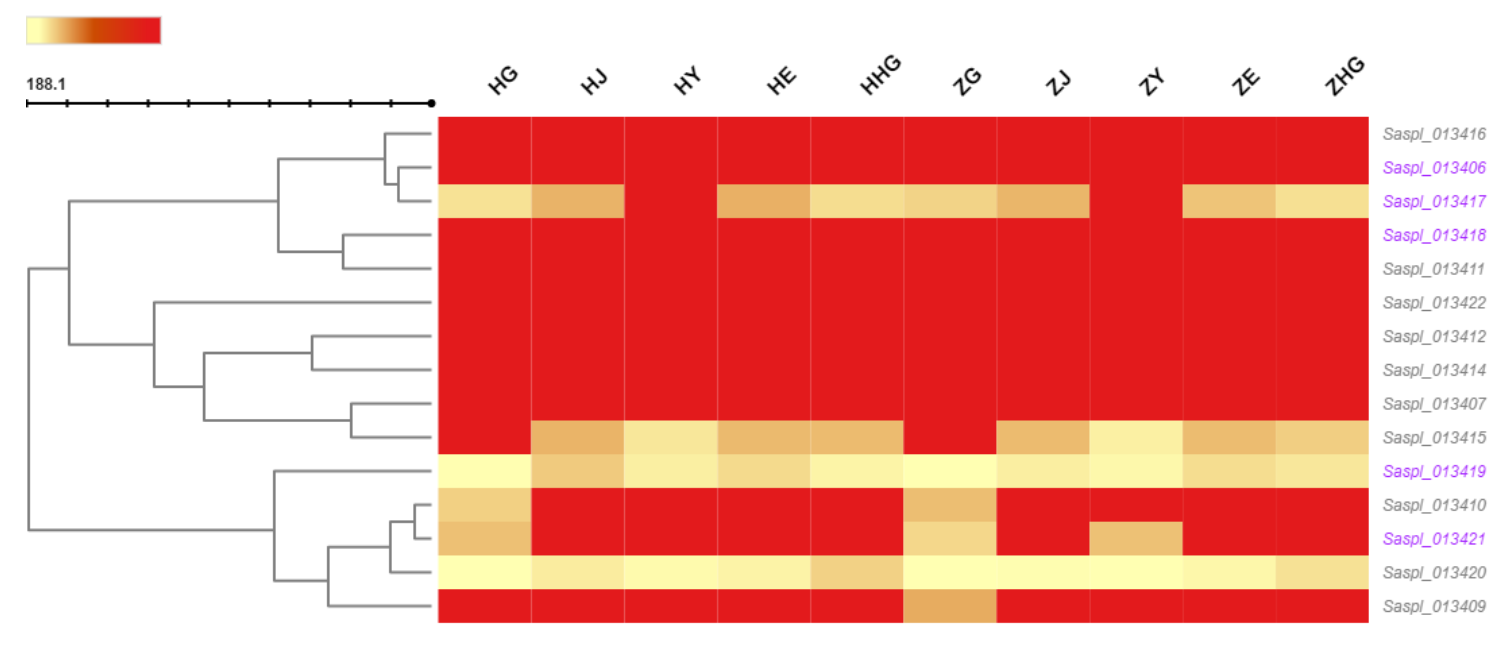
**

1. **scaffold21 - Cluster 29 – Saccharide**

**
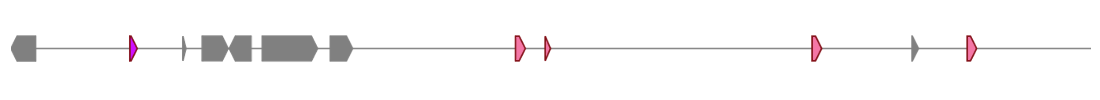
**

**
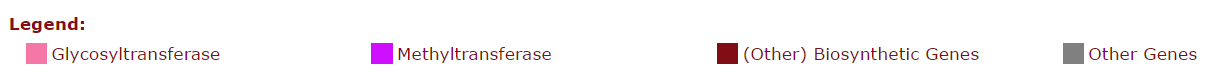
**

**
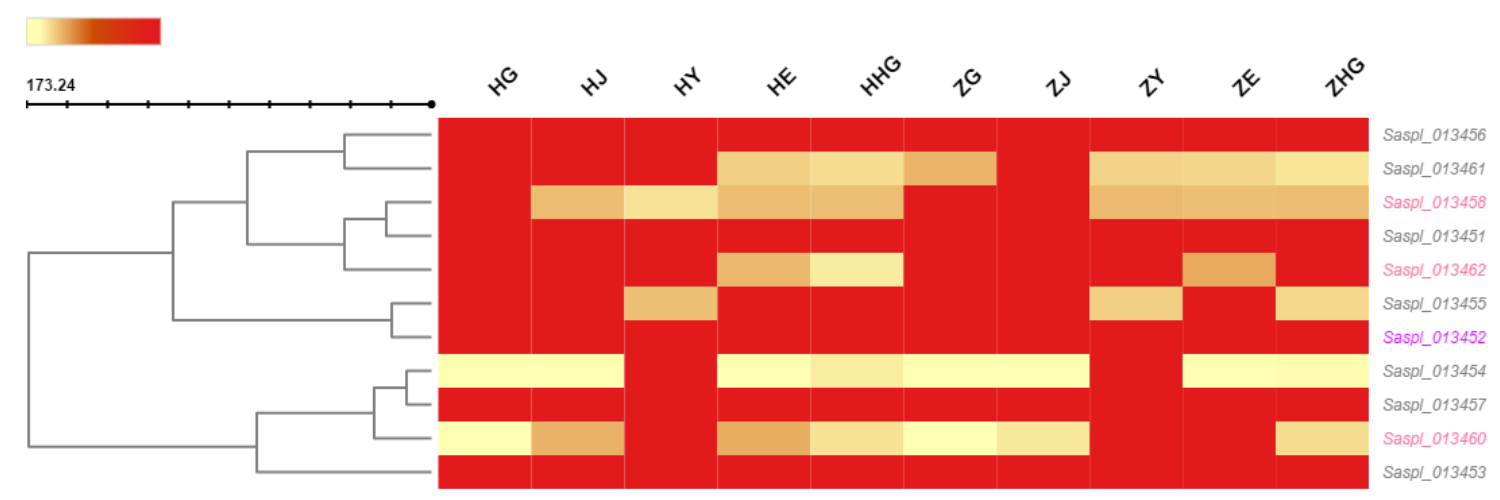
**

1. **scaffold22 - Cluster 30 – Alkaloid**

**
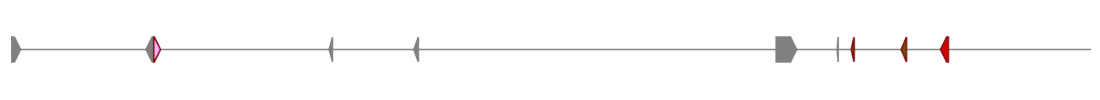
**

**
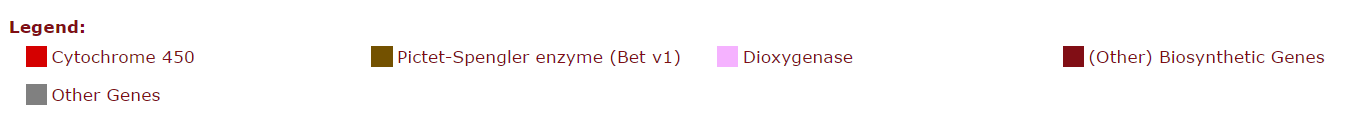
**

**
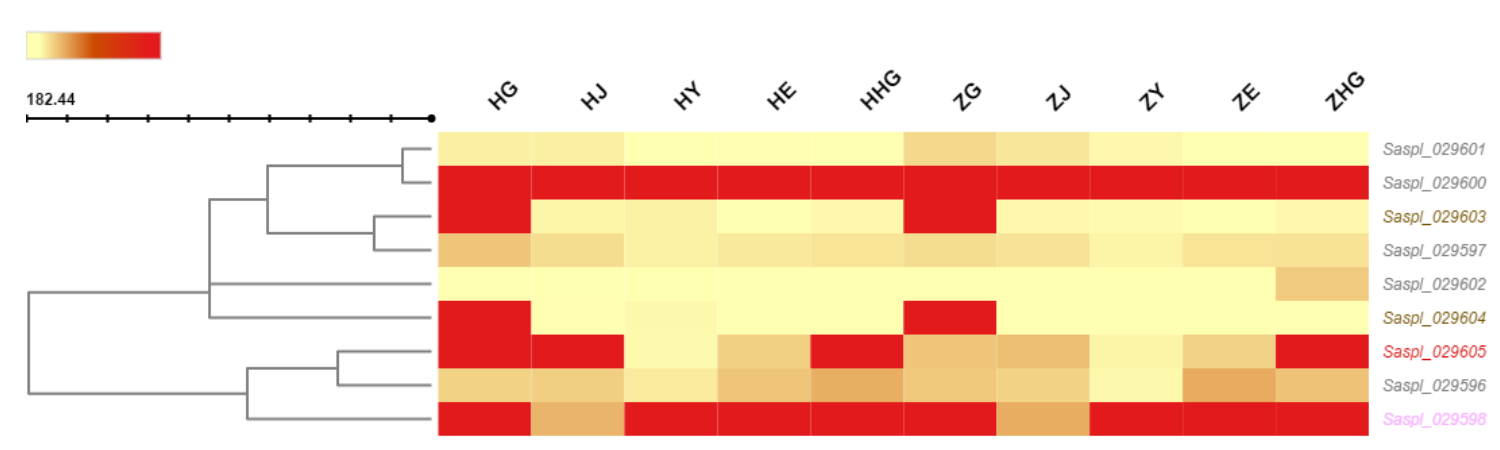
**

1. **scaffold227 - Cluster 31 – Saccharide**

**
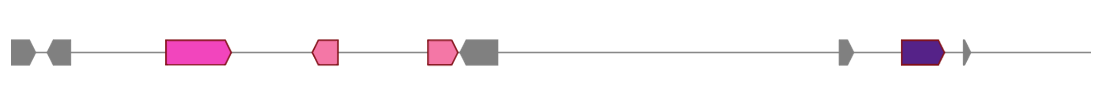
**

**
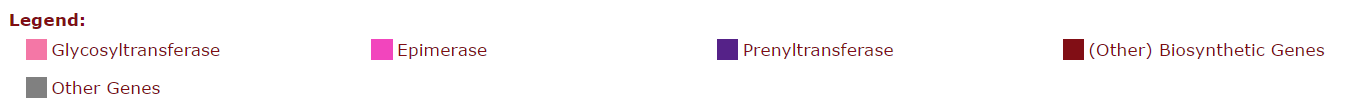
**

**
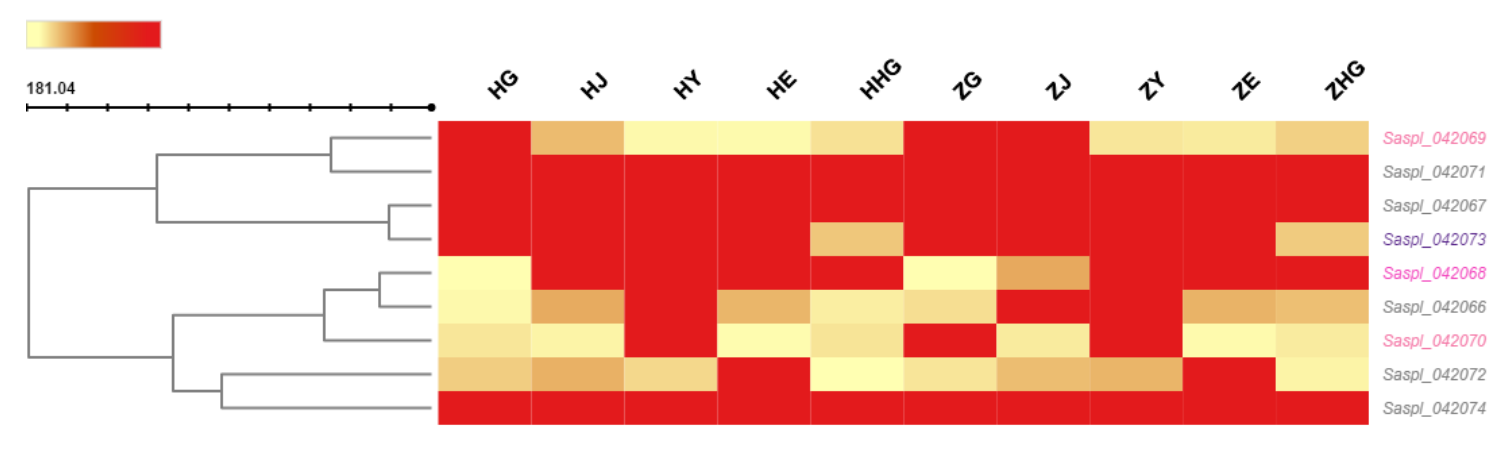
**

1. **scaffold266 - Cluster 32 – Saccharide**

**
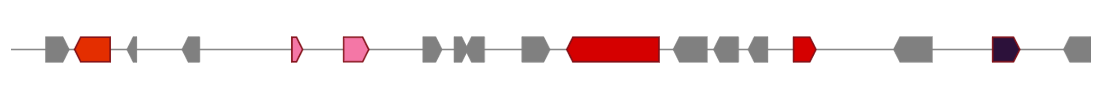
**

**
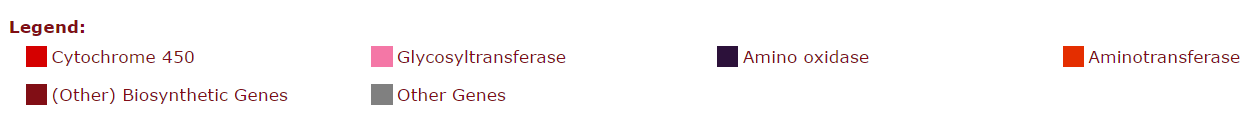
**

**
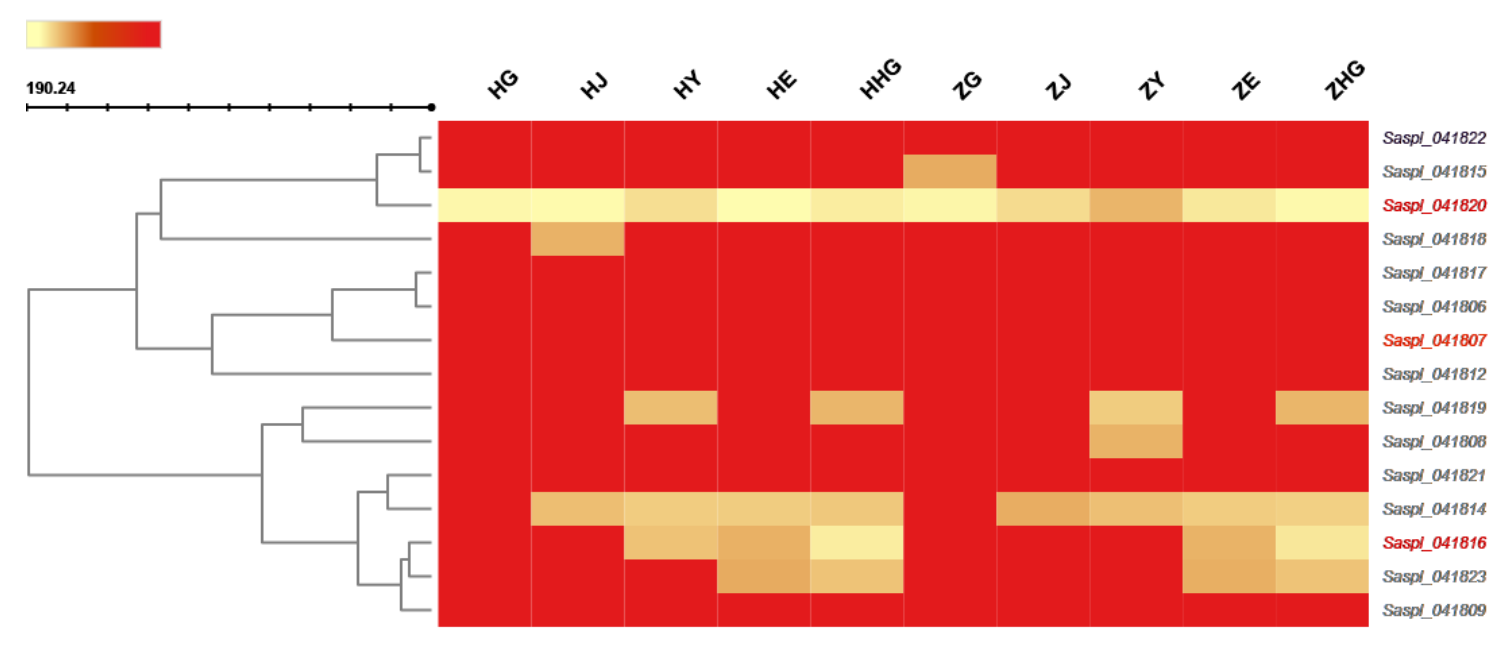
**

1. **scaffold28 - Cluster 33 - Putative**

**
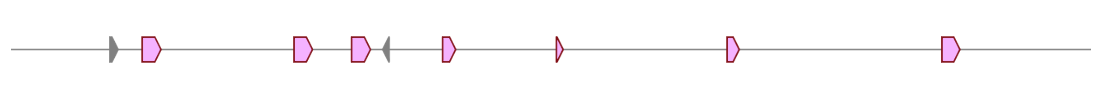
**

**
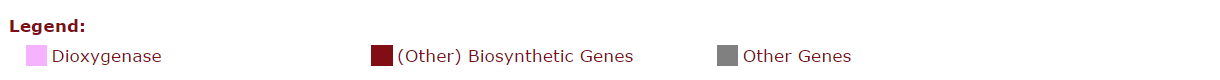
**

**
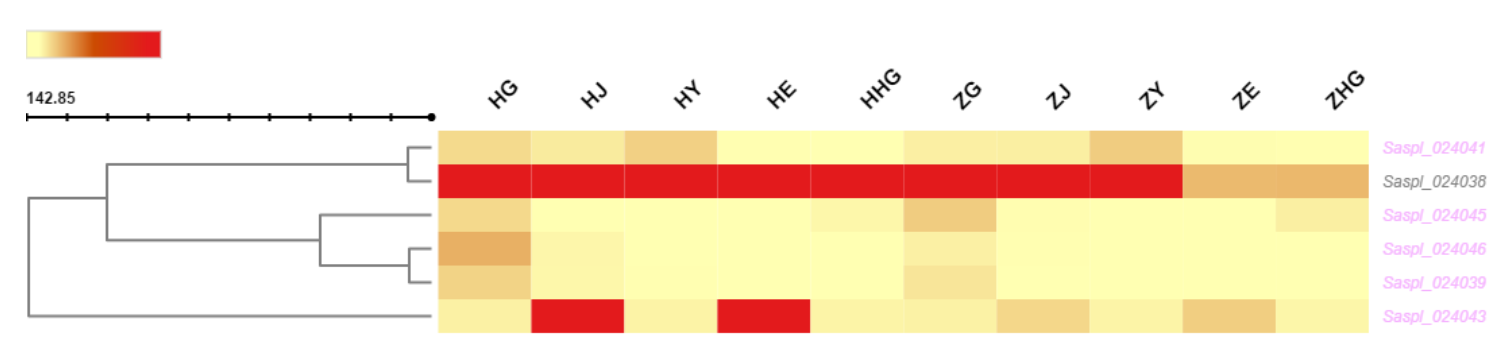
**

1. **scaffold28 - Cluster 34 – Lignan**

**
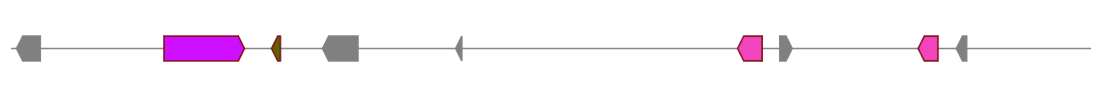
**

1. **scaffold29 - Cluster 35 – Alkaloid**

1. **scaffold3 - Cluster 36 - Putative**

1. **scaffold3 - Cluster 37 – Saccharide**

1. **scaffold30 - Cluster 38 – Saccharide**

1. **scaffold33 - Cluster 39 - Terpene-alkaloid**

1. **scaffold33 - Cluster 40 - Terpene-polyketide**

1. **scaffold33 - Cluster 41 – Putative**

1. **scaffold33 - Cluster 42 – Saccharide**

1. **scaffold37 - Cluster 43 – Saccharide**

1. **scaffold37 - Cluster 44 – Polyketide**

1. **scaffold39 - Cluster 45 – Alkaloid**

1. **scaffold4 - Cluster 46 – Terpene**

1. **scaffold40 - Cluster 47 - Saccharide**

1. **scaffold40 - Cluster 48 – Terpene**

1. **scaffold40 - Cluster 49 – Terpene**

1. **scaffold45 - Cluster 50 – Saccharide**

1. **scaffold45 - Cluster 51 - Terpene**

1. **scaffold48 - Cluster 52 - Saccharide-terpene**

1. **scaffold48 - Cluster 53 – Saccharide**

1. **scaffold49 - Cluster 54 - Alkaloid**

1. **scaffold52 - Cluster 55 – Terpene**

1. **scaffold52 - Cluster 56 – Polyketide**

1. **scaffold54 - Cluster 57 – Putative**

1. **scaffold55 - Cluster 58 - Lignan**

1. **scaffold59 - Cluster 59 – Lignan**

1. **scaffold6 - Cluster 60 - Alkaloid**

1. **scaffold6 - Cluster 61 – Saccharide**

1. **scaffold61 - Cluster 62 – Saccharide**

1. **scaffold62 - Cluster 63 – Terpene**

1. **scaffold62 - Cluster 64 – Saccharide**

1. **scaffold65 - Cluster 65 - Lignan-saccharide**

1. **scaffold65 - Cluster 66 – Saccharide**

1. **scaffold66 - Cluster 67 – Saccharide**

1. **scaffold66 - Cluster 68 – Putative**

1. **scaffold67 - Cluster 69 – Alkaloid**

1. **scaffold68 - Cluster 70 – Terpene**

1. **scaffold69 - Cluster 71 – Saccharide**

1. **scaffold69 - Cluster 72 - Saccharide-terpene**

1. **scaffold7 - Cluster 73 – Polyketide**

1. **scaffold72 - Cluster 74 - Saccharide**

1. **scaffold73 - Cluster 75 – Lignan**

1. **scaffold74 - Cluster 76 – Putative**

1. **scaffold8 - Cluster 77 – Saccharide**

1. **scaffold8 - Cluster 78 – Saccharide**

1. **scaffold89 - Cluster 79 – Saccharide**

1. **scaffold9 - Cluster 80 – Alkaloid**

1. **scaffold9 - Cluster 81 – Putative**

1. **scaffold9 - Cluster 82 – Lignan**

1. **scaffold9 - Cluster 83 – Saccharide**

1. **scaffold90 - Cluster 84 – Saccharide**

1. **scaffold98 - Cluster 85 – Alkaloid**
