# Supplementary material for: High-quality assembly of the reference genome for scarlet sage, Salvia splendens, an economically important ornamental plant
Source: Gigascience. 2018 Jun 19;7(7):giy068. doi: 10.1093/gigascience/giy068 (PMC6030905; doi:10.1093/gigascience/giy068)
Supplement: Additional Files [file giy068_supplemental_files.zip › Supplementary_File_3.docx]

1. **C220359 - Cluster 1 – Alkaloid**

**
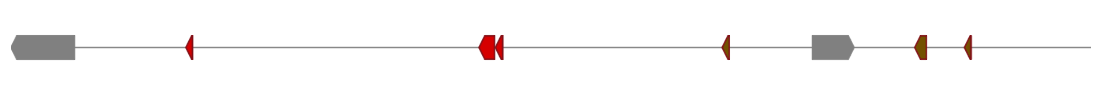
**

**
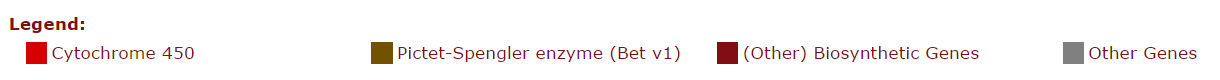
**

1. **C221085 - Cluster 2 – Saccharide**

**
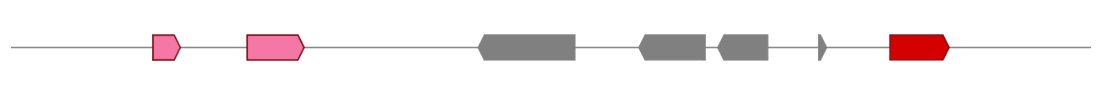
**

**
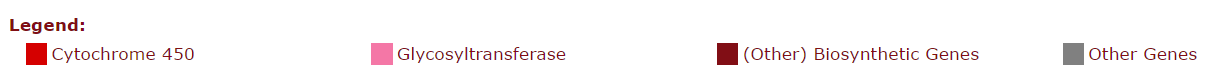
**

1. **C222127 - Cluster 3 – Alkaloid**

**
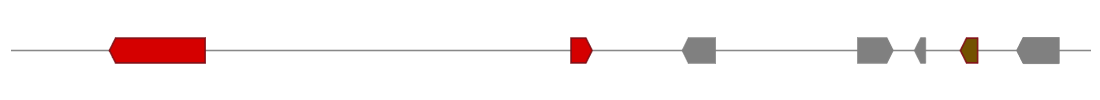
**

**
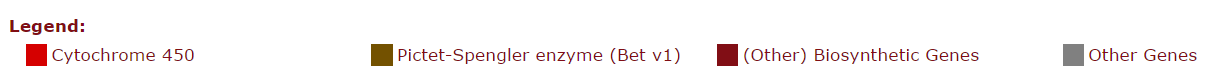
**

1. **scaffold10570 - Cluster 4 – Polyketide**

**
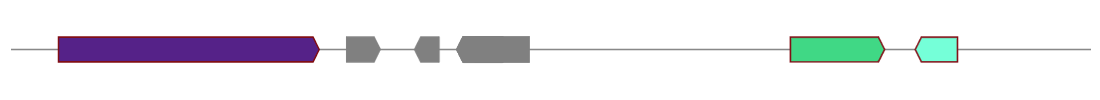
**

**
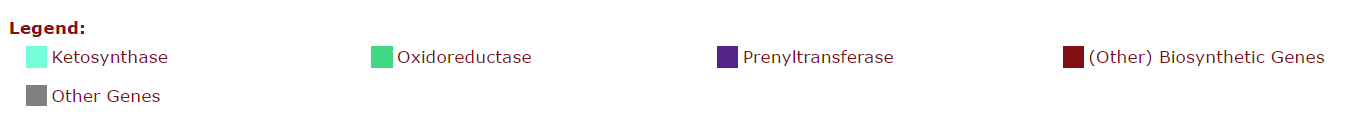
**

1. **scaffold1071 - Cluster 5 – Terpene**

**
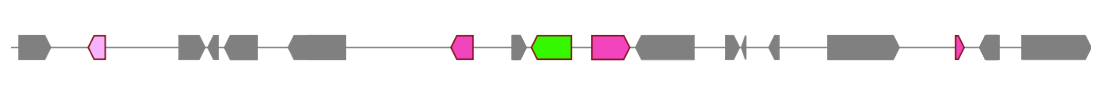
**

**
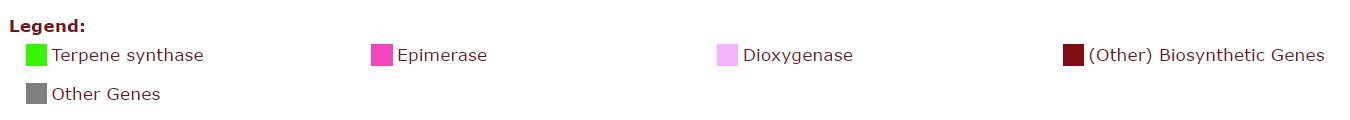
**

1. **scaffold11808 - Cluster 6 – Saccharide**

**
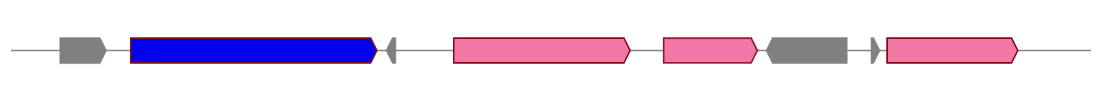
**

**
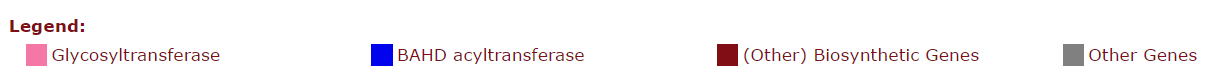
**

1. **scaffold11850 - Cluster 7 – Lignan**

**
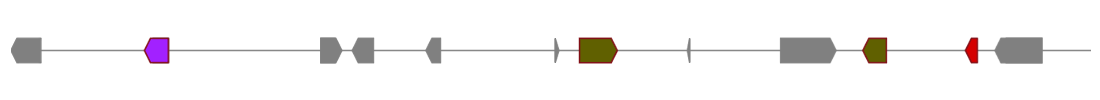
**

**
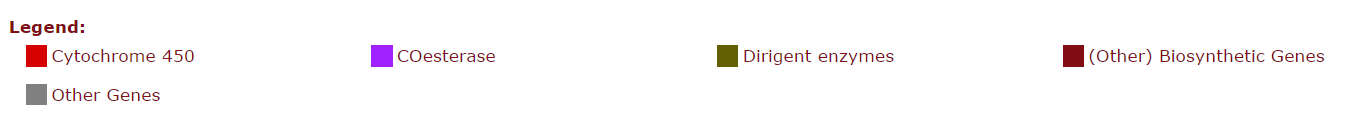
**

1. **scaffold12064 - Cluster 8 – Putative**

**
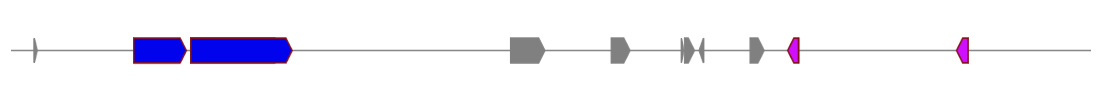
**

**
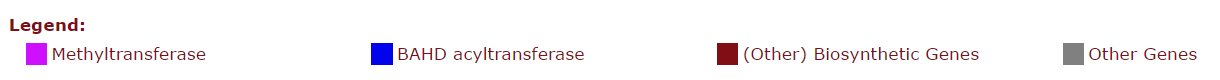
**

1. **scaffold12300 - Cluster 9 – Putative**

**
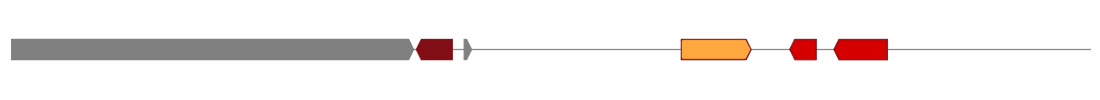
**

**
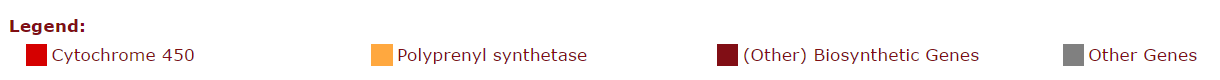
**

1. **scaffold12635 - Cluster 10 – Polyketide**

**
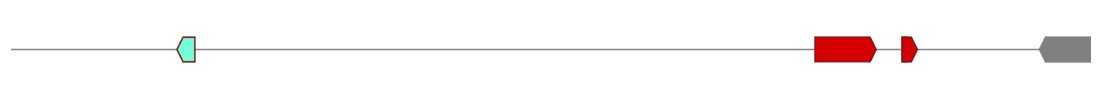
**

**
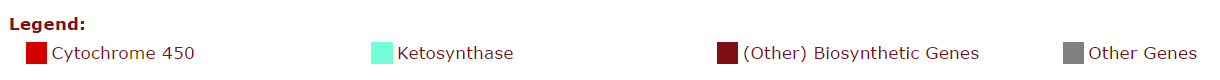
**

1. **scaffold1833 - Cluster 11 – Saccharide**

**
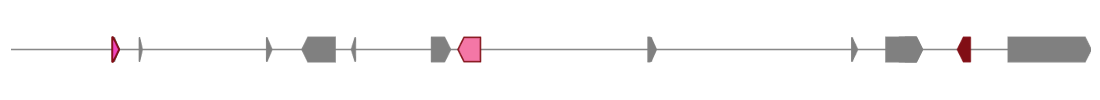
**

**
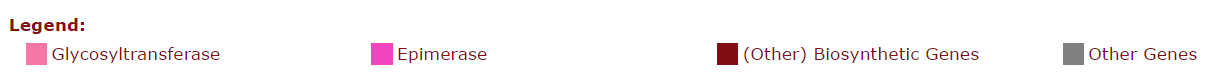
**

1. **scaffold2714 - Cluster 12 – Saccharide**

**
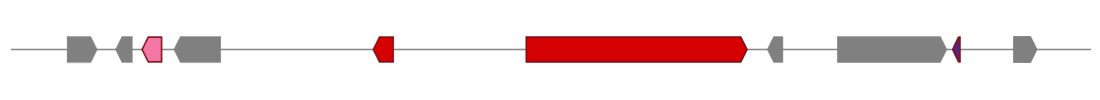
**

**
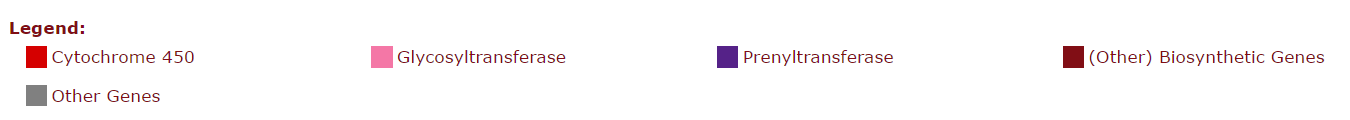
**

1. **scaffold2771 - Cluster 13 – Lignan**

**
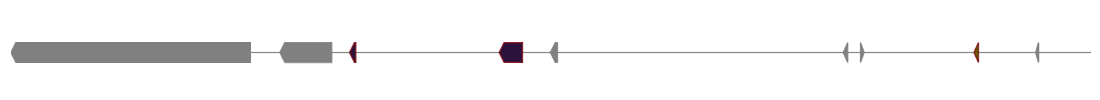
**

**
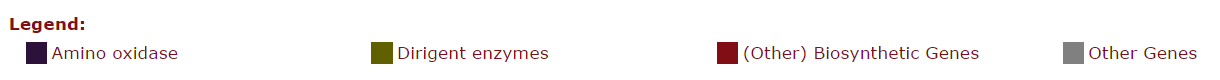
**

1. **scaffold3541 - Cluster 14 – Saccharide**

**
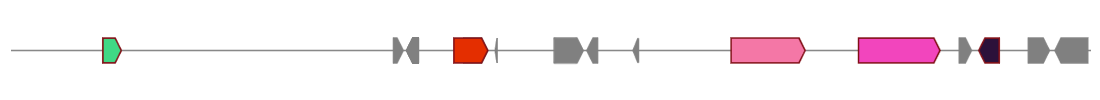
**

**
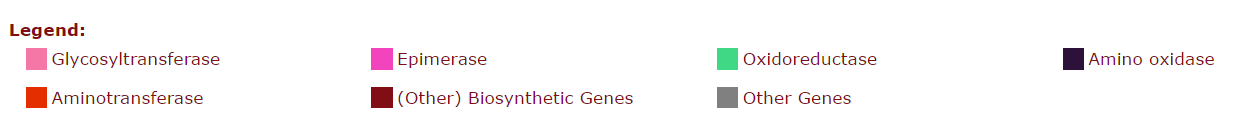
**

1. **scaffold4222 - Cluster 15 – Alkaloid**

**
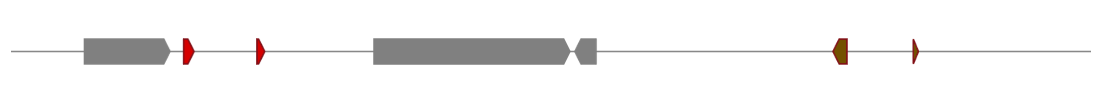
**

**
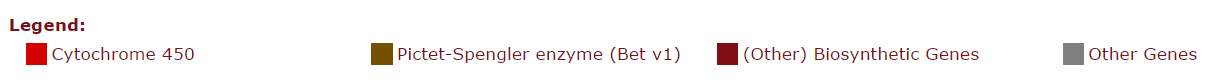
**

1. **scaffold431 - Cluster 16 – Saccharide**

**
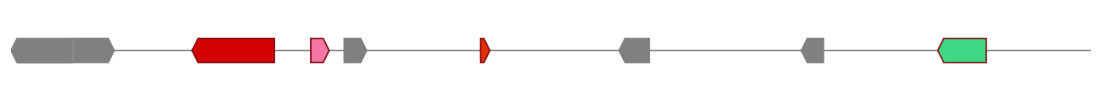
**

**
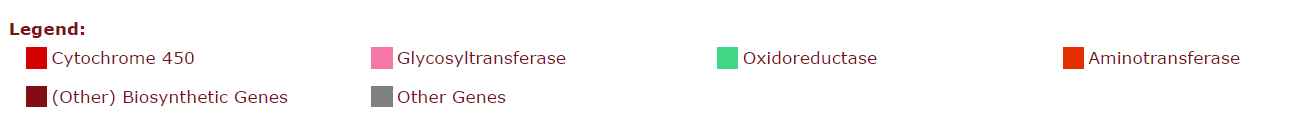
**

1. **scaffold4341 - Cluster 17 – Saccharide**

**
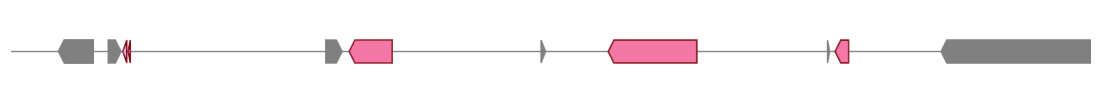
**

**
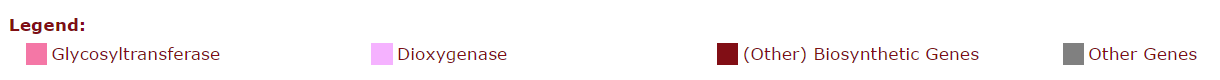
**

1. **scaffold4651 - Cluster 18 – Terpene**

**
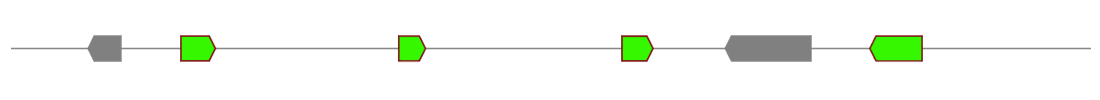
**

**
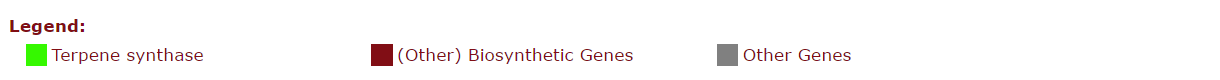
**

1. **scaffold6904 - Cluster 19 – Lignan**

**
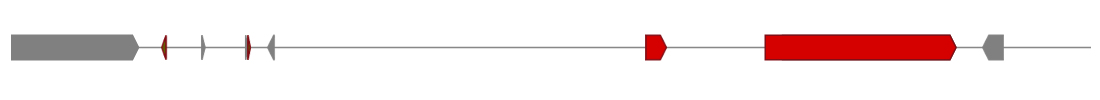
**

**
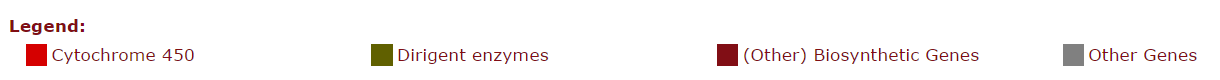
**

1. **scaffold726 - Cluster 20 – Polyketide**

**
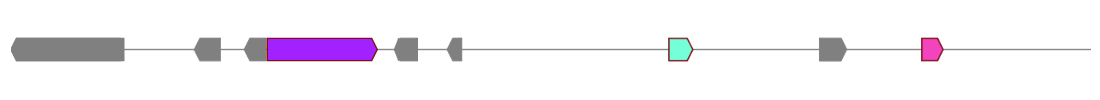
**

**
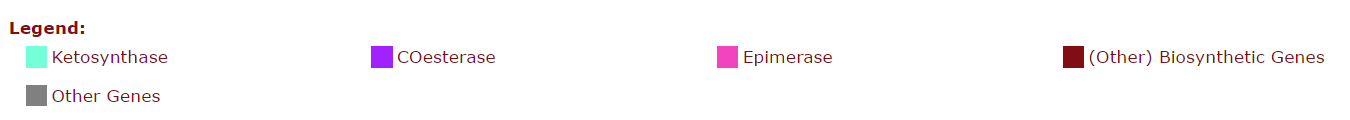
**

1. **scaffold7646 - Cluster 21 – Lignan**

**
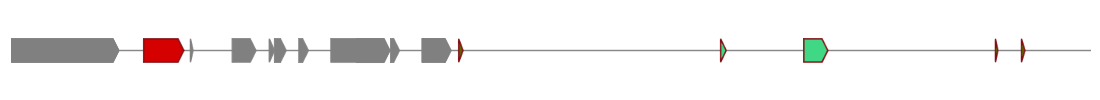
**

**
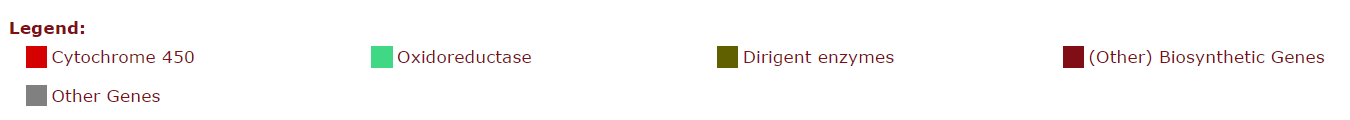
**

1. **scaffold775 - Cluster 22 – Putative**

**
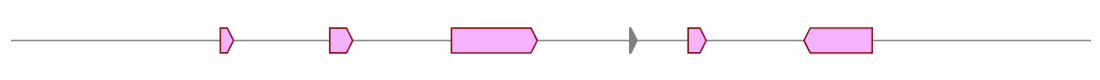
**

**
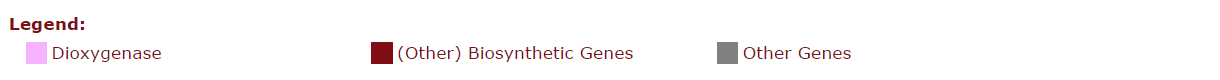
**

1. **scaffold8056 - Cluster 23 – Saccharide**

**
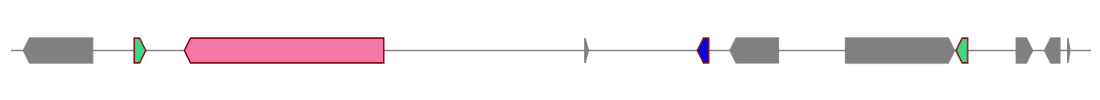
**

**
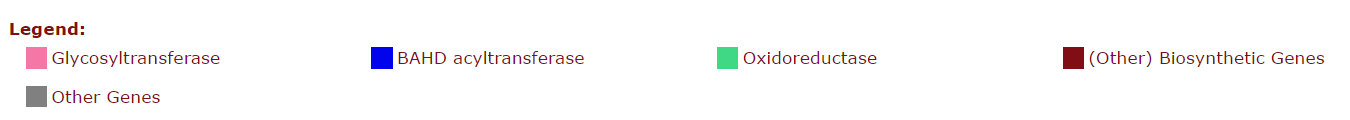
**
