# Supplementary material for: High-quality assembly of the reference genome for scarlet sage, Salvia splendens, an economically important ornamental plant
Source: Gigascience. 2018 Jun 19;7(7):giy068. doi: 10.1093/gigascience/giy068 (PMC6030905; doi:10.1093/gigascience/giy068)
Supplement: Additional Files [file giy068_supplemental_files.zip › Table_S10.docx]

| **Species** | **Unique groups** | **Unique genes** | **Single-copy groups** | **Duplicated groups** | **Duplicated genes** |
| --- | --- | --- | --- | --- | --- |
| *Salvia splendens* | 1,306 | 3,797 | 3,957 | 12,203 | 40,536 |
| *Salvia miltiorrhiza Xu* | 500 | 1,562 | 10,923 | 4,159 | 13,497 |
| *Salvia miltiorrhiza Zhang* | 717 | 1,949 | 11,127 | 3,624 | 9,534 |
| *Fraxinus excelsior* | 227 | 496 | 9,833 | 6,985 | 19,831 |
| *Olea europaea* | 1,410 | 4,973 | 8,288 | 9,818 | 31,794 |
| *Mimulus guttatus* | 466 | 1,513 | 10,726 | 4,239 | 13,249 |
| *Utricularia gibba* | 541 | 6,801 | 7,494 | 3,064 | 13,896 |
| *Sesamum indicum* | 351 | 2,490 | 9,931 | 4,122 | 13,399 |
| *Coffea canephora* | 524 | 1,607 | 11,508 | 2,763 | 9,135 |
| *Solanum lycopersicum* | 875 | 3,476 | 10,228 | 4,697 | 15,268 |
| *Daucus carota* | 1,138 | 4,639 | 8,676 | 5,304 | 17,144 |
| *Beta vulgaris* | 528 | 2,566 | 10,438 | 2,499 | 11,300 |
| *Vitis vinifera* | 629 | 1,757 | 10,416 | 2,970 | 8,730 |
| *Arabidopsis thaliana* | 776 | 2,729 | 8,698 | 4,701 | 14,454 |
| *Populus trichocarpa* | 1,087 | 3,337 | 6,129 | 9,104 | 26,886 |
| *Oryza sativa* | 2,082 | 9,091 | 7,947 | 5,419 | 20,527 |
