# Supplementary material for: High-quality assembly of the reference genome for scarlet sage, Salvia splendens, an economically important ornamental plant
Source: Gigascience. 2018 Jun 19;7(7):giy068. doi: 10.1093/gigascience/giy068 (PMC6030905; doi:10.1093/gigascience/giy068)
Supplement: Additional Files [file giy068_supplemental_files.zip › Table_S12.docx]

| **KO category** | **p_value** | **q_value** | **numEPInCat** | **numInCat** | **Pathway** | **Class** |
| --- | --- | --- | --- | --- | --- | --- |
| **ko00250** | **8.73E-05** | **0.01144** | **34** | **35** | **Alanine, aspartate and glutamate metabolism** | **Metabolism; Amino acid metabolism** |
| ko01200 | 0.003506263 | 0.162635 | 146 | 183 | Carbon metabolism | Metabolism; Overview |
| ko00920 | 0.003724468 | 0.162635 | 27 | 29 | Sulfur metabolism | Metabolism; Energy metabolism |
| ko00620 | 0.01028444 | 0.336815 | 50 | 59 | Pyruvate metabolism | Metabolism; Carbohydrate metabolism |
| ko00630 | 0.020367557 | 0.447536 | 43 | 51 | Glyoxylate and dicarboxylate metabolism | Metabolism; Carbohydrate metabolism |
| ko03015 | 0.024738388 | 0.447536 | 77 | 96 | mRNA surveillance pathway | Genetic Information Processing; Translation |
| ko00310 | 0.027705822 | 0.447536 | 31 | 36 | Lysine degradation | Metabolism; Amino acid metabolism |
| ko00562 | 0.027705822 | 0.447536 | 31 | 36 | Inositol phosphate metabolism | Metabolism; Carbohydrate metabolism |
| ko00450 | 0.030746754 | 0.447536 | 15 | 16 | Selenocompound metabolism | Metabolism; Metabolism of other amino acids |
| ko00860 | 0.040369251 | 0.528837 | 36 | 43 | Porphyrin and chlorophyll metabolism | Metabolism; Metabolism of cofactors and vitamins |
