# Supplementary material for: High-quality assembly of the reference genome for scarlet sage, Salvia splendens, an economically important ornamental plant
Source: Gigascience. 2018 Jun 19;7(7):giy068. doi: 10.1093/gigascience/giy068 (PMC6030905; doi:10.1093/gigascience/giy068)
Supplement: Additional Files [file giy068_supplemental_files.zip › Table_S2.docx]

| **K-mer value** | **Raw peak** | **Amount of**  **k-mer used** | **Coverage** | **Genome size** | **Heterozygosity rate (%)** | **Repeat rate (%)** | **Error rate (%)** |
| --- | --- | --- | --- | --- | --- | --- | --- |
| 17 | 31 | 2.00E+10 | 33.19 | 7.00E+08 | 0.06 | 47.99 | 0.27 |
