# Supplementary material for: High-quality assembly of the reference genome for scarlet sage, Salvia splendens, an economically important ornamental plant
Source: Gigascience. 2018 Jun 19;7(7):giy068. doi: 10.1093/gigascience/giy068 (PMC6030905; doi:10.1093/gigascience/giy068)
Supplement: Additional Files [file giy068_supplemental_files.zip › Table_S3.docx]

| **Versions of assembly** | **Strategy** | **Assembled**  **genome size (Mb)** | **Sequence number** | **N50** | **L50** | **Max. length (Mb)** | **Gene completeness (%)** |
| --- | --- | --- | --- | --- | --- | --- | --- |
| v0.1 | CANU | 808 | 2,306 | 2.06 Mb | 109 | 8.88 | 92.10 |
| v0.2 | MECAT | 790 | 2,597 | 856 Kb | 264 | 5.02 | 92.00 |
| v0.3 | CANU+FALCON | 827 | 4,480 | 1.09 Mb | 224 | 4.42 | NA |
| v0.4 | CANU+SMARTDENOVO | 747 | 1,999 | 728 Kb | 297 | 6.24 | NA |
| v1.0 | v0.1+arrow | 808 | 2,306 | 2.06 Mb | 109 | 8.88 | 92.20 |
| v1.1 | v1.0+SSPACE×2+gapcloser | 810 | 2,259/1,525* | 2.1/3.12 Mb* | 106/73* | 10.8/12.9* | 92.00 |
| v1.2 | v1.1+arrow+pilon×2 | 809 | 2,204/1,525* | 2.26/3.12 Mb* | 100/73* | 10.8/12.9* | 92.20 |

NA: not available; * statistics for contigs/scaffolds.
