# Supplementary material for: High-quality assembly of the reference genome for scarlet sage, Salvia splendens, an economically important ornamental plant
Source: Gigascience. 2018 Jun 19;7(7):giy068. doi: 10.1093/gigascience/giy068 (PMC6030905; doi:10.1093/gigascience/giy068)
Supplement: Additional Files [file giy068_supplemental_files.zip › Table_S4.docx]

| **Class** | **Family** | **Number** | **Length (bp)** | **Percentage (%)** |
| --- | --- | --- | --- | --- |
| LTR |  | 184,299 | 214,338,127 | 26.49 |
|  | Cassandra | 275 | 45,745 | 0.01 |
|  | Caulimovirus | 1,064 | 2,144,453 | 0.27 |
|  | Copia | 63,543 | 64,109,865 | 7.92 |
|  | ERV1 | 694 | 248,566 | 0.03 |
|  | Gypsy | 114,681 | 146,869,275 | 18.15 |
|  | Pao | 81 | 31,895 | 0 |
| LINE |  | 20,327 | 13,411,300 | 1.66 |
|  | L1 | 16,639 | 12,734,947 | 1.57 |
|  | L1-Tx1 | 2,733 | 516,876 | 0.06 |
|  | L2 | 955 | 159,477 | 0.02 |
| SINE |  | 1,150 | 185,852 | 0.02 |
|  | ID | 912 | 148,049 | 0.02 |
| DNA |  | 276,484 | 96,409,353 | 11.91 |
|  | CMC-EnSpm | 26,393 | 9,202,034 | 1.14 |
|  | IS3EU | 5,786 | 2,022,975 | 0.25 |
|  | Kolobok-T2 | 5,983 | 717,233 | 0.09 |
|  | MULE-MuDR | 7,158 | 2,058,592 | 0.25 |
|  | Maverick | 418 | 77,353 | 0.01 |
|  | MuLE-MuDR | 70,935 | 13,854,618 | 1.71 |
|  | Novosib | 1,651 | 2,704,122 | 0.33 |
|  | PIF-Harbinger | 65,532 | 39,811,962 | 4.92 |
|  | Sola-1 | 1,933 | 431,416 | 0.05 |
|  | TcMar-Stowaway | 43,320 | 8,118,066 | 1 |
|  | TcMar-Tc1 | 898 | 202,994 | 0.03 |
|  | hAT-Ac | 35,778 | 13,880,935 | 1.72 |
|  | hAT-Tag1 | 5,124 | 1,549,495 | 0.19 |
|  | hAT-Tip100 | 4,204 | 1,411,922 | 0.17 |
| RC |  | 9,935 | 2,139,972 | 0.26 |
|  | Helitron | 9,935 | 2,139,972 | 0.26 |
| Unknown |  | 446,828 | 128,843,956 | 15.92 |
| Simple_repeat | | 147,578 | 8,760,230 | 1.08 |
| Low_complexity | | 26,996 | 1,352,088 | 0.17 |
| Total |  | 1,113,597 | 465,440,878 | 57.52 |
