# Supplementary material for: High-quality assembly of the reference genome for scarlet sage, Salvia splendens, an economically important ornamental plant
Source: Gigascience. 2018 Jun 19;7(7):giy068. doi: 10.1093/gigascience/giy068 (PMC6030905; doi:10.1093/gigascience/giy068)
Supplement: Additional Files [file giy068_supplemental_files.zip › Table_S5.docx]

| **Pipelines** | **Genes identified** | **Transcripts** | **Length of the assemblied genes (bp)** | **Length of the assemblied transcripts (bp)** | **N50 of the transcripts (bp)** | **Average length of the genes (bp)** | **Average length of the transcripts (bp)** |
| --- | --- | --- | --- | --- | --- | --- | --- |
| Trinity denovo | 75,977 | 140,181 | 56,344,561 | 136,273,498 | 1,542 | 741.60 | 972.13 |
| Trinity genome-guided | 81,035 | 114,506 | 105,835,162 | 172,732,297 | 2,055 | 1,306.04 | 1,508.50 |
| StringTie | 84,554 | 149,810 | 231,411,572 | 330,734,262 | 3,032 | 2,736.85 | 2,207.69 |
| Cufflinks | 71,176 | 99,198 | 176,635,225 | 216,876,515 | 2,675 | 2,481.67 | 2,186.30 |
| Merged transcriptome | NA | 192,169 | NA | 434,461,665 | 2,981 | NA | 2,260.83 |
