# Supplementary material for: High-quality assembly of the reference genome for scarlet sage, Salvia splendens, an economically important ornamental plant
Source: Gigascience. 2018 Jun 19;7(7):giy068. doi: 10.1093/gigascience/giy068 (PMC6030905; doi:10.1093/gigascience/giy068)
Supplement: Additional Files [file giy068_supplemental_files.zip › Table_S6.docx]

| **Gene number** | **Gene number**  **(AED < 0.5)** | **Average length of gene region (bp)** | **Average gene length (bp)** | **Average CDS length (bp)** | **Average exons per gene** | **Average exon length (bp)** | **Average intron length (bp)** |
| --- | --- | --- | --- | --- | --- | --- | --- |
| 54,008 | 52,338 (96.90%) | 3,430.43 | 1,696.34 | 1,293.62 | 6.38 | 265.94 | 323.4 |
