# Supplementary material for: High-quality assembly of the reference genome for scarlet sage, Salvia splendens, an economically important ornamental plant
Source: Gigascience. 2018 Jun 19;7(7):giy068. doi: 10.1093/gigascience/giy068 (PMC6030905; doi:10.1093/gigascience/giy068)
Supplement: Additional Files [file giy068_supplemental_files.zip › Table_S7.docx]

|  | **BUSCO groups** | **Percentage (%)** |
| --- | --- | --- |
| Complete BUSCOs | 1,326 | 92.08 |
| Complete and single-copy BUSCOs | 466 | 32.36 |
| Complete and duplicated BUSCOs | 860 | 59.72 |
| Fragmented BUSCOs | 35 | 2.43 |
| Missing BUSCOs | 79 | 5.49 |
| Total BUSCO groups searched | 1,440 | 100.00 |
