# Supplementary material for: High-quality assembly of the reference genome for scarlet sage, Salvia splendens, an economically important ornamental plant
Source: Gigascience. 2018 Jun 19;7(7):giy068. doi: 10.1093/gigascience/giy068 (PMC6030905; doi:10.1093/gigascience/giy068)
Supplement: Additional Files [file giy068_supplemental_files.zip › Table_S8.docx]

|  | **Databases** | **Count** | **Percentage (%)** |
| --- | --- | --- | --- |
| Total genes |  | 54,008 | 100 |
| Annotated | NR | 51,081 | 94.60 |
|  | Swiss_Prot | 34,234 | 63.40 |
|  | TrEMBL | 50,478 | 93.50 |
|  | Pfam | 44,326 | 82.10 |
|  | KOG | 48,900 | 90.50 |
|  | GO | 42,571 | 78.80 |
|  | KO | 20,199 | 37.40 |
| Unannotated |  | 2,882 | 5.34 |
