# Supplementary material for: High-quality assembly of the reference genome for scarlet sage, Salvia splendens, an economically important ornamental plant
Source: Gigascience. 2018 Jun 19;7(7):giy068. doi: 10.1093/gigascience/giy068 (PMC6030905; doi:10.1093/gigascience/giy068)
Supplement: Additional Files [file giy068_supplemental_files.zip › Table_S9.docx]

| **Species** | **Links** | **Version** | **Genome size (Mb)** | **Scaffold N50 (Mb)** | **Genes** | **Reference** |
| --- | --- | --- | --- | --- | --- | --- |
| *Salvia miltiorrhiza* | http://www.ndctcm.org/shujukujieshao/2015-04-23/27.html | version 3 | 538 | 0.05 | 30,478 | [1] |
| *Salvia miltiorrhiza* | http://www.herbal-genome.cn | EVM%20prediction | 660 | 1.20 | 34,598 | [2] |
| *Fraxinus excelsior* | http://www.ashgenome.org/ | BATG-0.5 | 867 | 0.10 | 38852 | [3] |
| *Olea europaea* | https://phytozome.jgi.doe.gov | v1.0 | 1,140 | 0.36 | 50,684 | [4] |
| *Mimulus guttatus* | https://phytozome.jgi.doe.gov | v2.0 | 313 | 21.20 | 28140 | [5] |
| *Utricularia gibba* | <https://genomevolution.org/coge/GenomeInfo.pl?gid=29027> | 1.1 | 100 | 3.40 | 29,666 | [6] |
| *Sesamum indicum* | <http://ocri-genomics.org/Sinbase/> | v1.0 | 274 | 2.10 | 27,148 | [7] |
| *Coffea canephora* | http://coffee-genome.org/ | - | 569 | 1.26 | 25,574 | [8] |
| *Solanum lycopersicum* | https://phytozome.jgi.doe.gov | ITAG2.4 | 823 | 66.00 | 34,727 | [9] |
| *Daucus carota* | https://phytozome.jgi.doe.gov | v2.0 | 421 | 12.70 | 32,113 | [10] |
| *Vitis vinifera* | https://phytozome.jgi.doe.gov | Genoscope.12X | 486 | 23.00 | 26,346 | [11] |
| *Arabidopsis thaliana* | https://phytozome.jgi.doe.gov | TAIR10 | 135 | 22.40 | 27,416 | [12] |
| *Populus trichocarpa* | https://phytozome.jgi.doe.gov | v3.0 | 423 | 19.50 | 41,335 | [13] |
| *Oryza sativa* | https://phytozome.jgi.doe.gov | MSU_v7.0 | 372 | 28.60 | 42,189 | [14] |
| *Beta_vulgaris* | ftp://ftp.ncbi.nlm.nih.gov/genomes/all/GCF/000/511/025/GCF_000511025.2_RefBeet-1.2.2 | RefBeet-1.2.2 | 566 | 2.10 | 40,406 | [15] |

-, no data.

1. Xu H, Song J, Luo H, Zhang Y, Li Q, Zhu Y, et al. Analysis of the Genome Sequence of the Medicinal Plant *Salvia miltiorrhiza*. Mol Plant. 2016;9 6:949-52. doi:10.1016/j.molp.2016.03.010.

2. Zhang G, Tian Y, Zhang J, Shu L, Yang S, Wang W, et al. Hybrid de novo genome assembly of the Chinese herbal plant danshen (*Salvia miltiorrhiza* Bunge). GigaScience. 2015;4 1:62. doi:10.1186/s13742-015-0104-3.

3. Sollars ESA, Harper AL, Kelly LJ, Sambles CM, Ramirez-Gonzalez RH, Swarbreck D, et al. Genome sequence and genetic diversity of European ash trees. Nature. 2016;541:212. doi:10.1038/nature20786.

4. Unver T, Wu Z, Sterck L, Turktas M, Lohaus R, Li Z, et al. Genome of wild olive and the evolution of oil biosynthesis. Proceedings of the National Academy of Sciences. 2017;114 44:E9413-E22. doi:10.1073/pnas.1708621114.

5. Hellsten U, Wright KM, Jenkins J, Shu S, Yuan Y, Wessler SR, et al. Fine-scale variation in meiotic recombination in Mimulus inferred from population shotgun sequencing. Proceedings of the National Academy of Sciences. 2013;110 48:19478-82. doi:10.1073/pnas.1319032110.

6. Lan T, Renner T, Ibarra-Laclette E, Farr KM, Chang T-H, Cervantes-Pérez SA, et al. Long-read sequencing uncovers the adaptive topography of a carnivorous plant genome. Proceedings of the National Academy of Sciences. 2017;114 22:E4435-E41. doi:10.1073/pnas.1702072114.

7. Wang L, Yu S, Tong C, Zhao Y, Liu Y, Song C, et al. Genome sequencing of the high oil crop sesame provides insight into oil biosynthesis. Genome Biology. 2014;15 2:R39. doi:10.1186/gb-2014-15-2-r39.

8. Denoeud F, Carretero-Paulet L, Dereeper A, Droc G, Guyot R, Pietrella M, et al. The coffee genome provides insight into the convergent evolution of caffeine biosynthesis. Science. 2014;345 6201:1181-4. doi:10.1126/science.1255274.

9. The Tomato Genome C. The tomato genome sequence provides insights into fleshy fruit evolution. Nature. 2012;485:635. doi:10.1038/nature11119.

10. Iorizzo M, Ellison S, Senalik D, Zeng P, Satapoomin P, Huang J, et al. A high-quality carrot genome assembly provides new insights into carotenoid accumulation and asterid genome evolution. Nature Genetics. 2016;48:657. doi:10.1038/ng.3565.

11. The French–Italian Public Consortium for Grapevine Genome C. The grapevine genome sequence suggests ancestral hexaploidization in major angiosperm phyla. Nature. 2007;449:463. doi:10.1038/nature06148.

12. Cheng C-Y, Krishnakumar V, Chan AP, Thibaud-Nissen F, Schobel S and Town CD. Araport11: a complete reannotation of the *Arabidopsis thaliana* reference genome. The Plant Journal. 2017;89 4:789-804. doi:10.1111/tpj.13415.

13. Tuskan GA, DiFazio S, Jansson S, Bohlmann J, Grigoriev I, Hellsten U, et al. The Genome of Black Cottonwood, *Populus trichocarpa* (Torr. &amp; Gray). Science. 2006;313 5793:1596-604. doi:10.1126/science.1128691.

14. Ouyang S, Zhu W, Hamilton J, Lin H, Campbell M, Childs K, et al. The TIGR Rice Genome Annotation Resource: improvements and new features. Nucleic Acids Research. 2007;35 suppl_1:D883-D7. doi:10.1093/nar/gkl976.

15. Dohm JC, Minoche AE, Holtgräwe D, Capella-Gutiérrez S, Zakrzewski F, Tafer H, et al. The genome of the recently domesticated crop plant sugar beet (*Beta vulgaris*). Nature. 2013;505:546. doi:10.1038/nature12817.
