# Supplementary figures and images for: High-quality assembly of the reference genome for scarlet sage, Salvia splendens, an economically important ornamental plant
Source: Gigascience. 2018 Jun 19;7(7):giy068. doi: 10.1093/gigascience/giy068 (PMC6030905; doi:10.1093/gigascience/giy068)

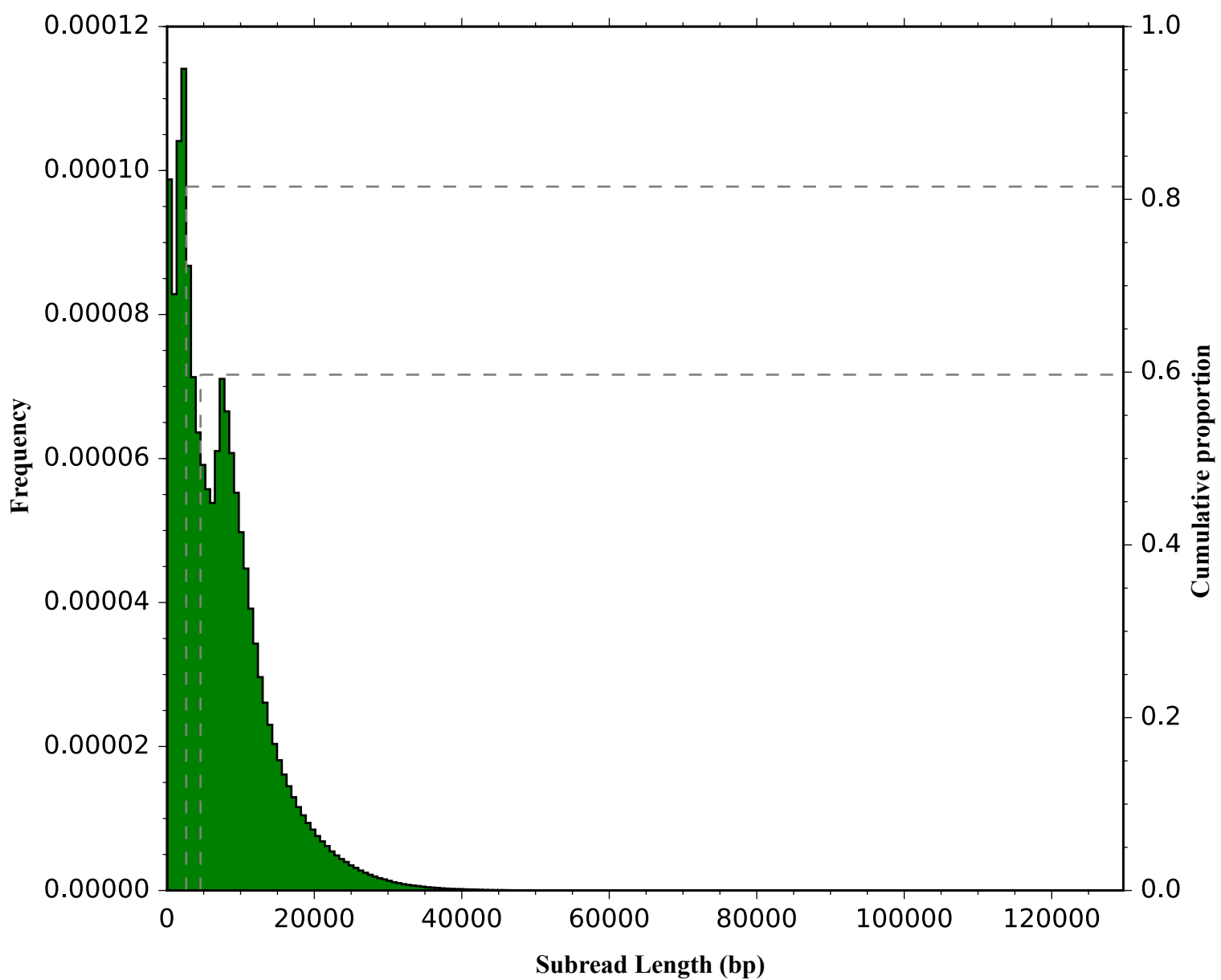

Supplement: Additional Files [file giy068_supplemental_files.zip › Fig_S1.pdf]

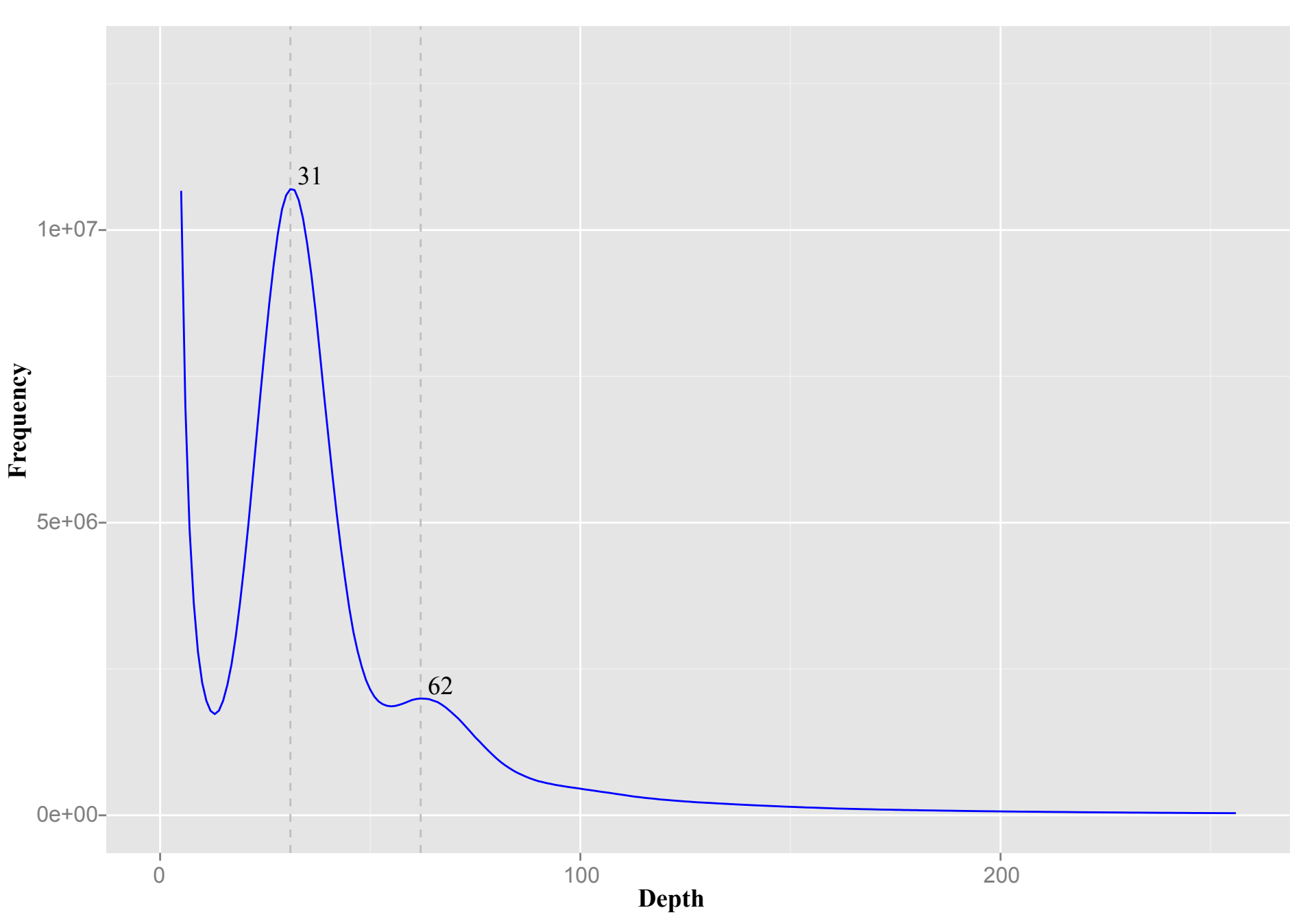

Supplement: Additional Files [file giy068_supplemental_files.zip › Fig_S2.pdf]

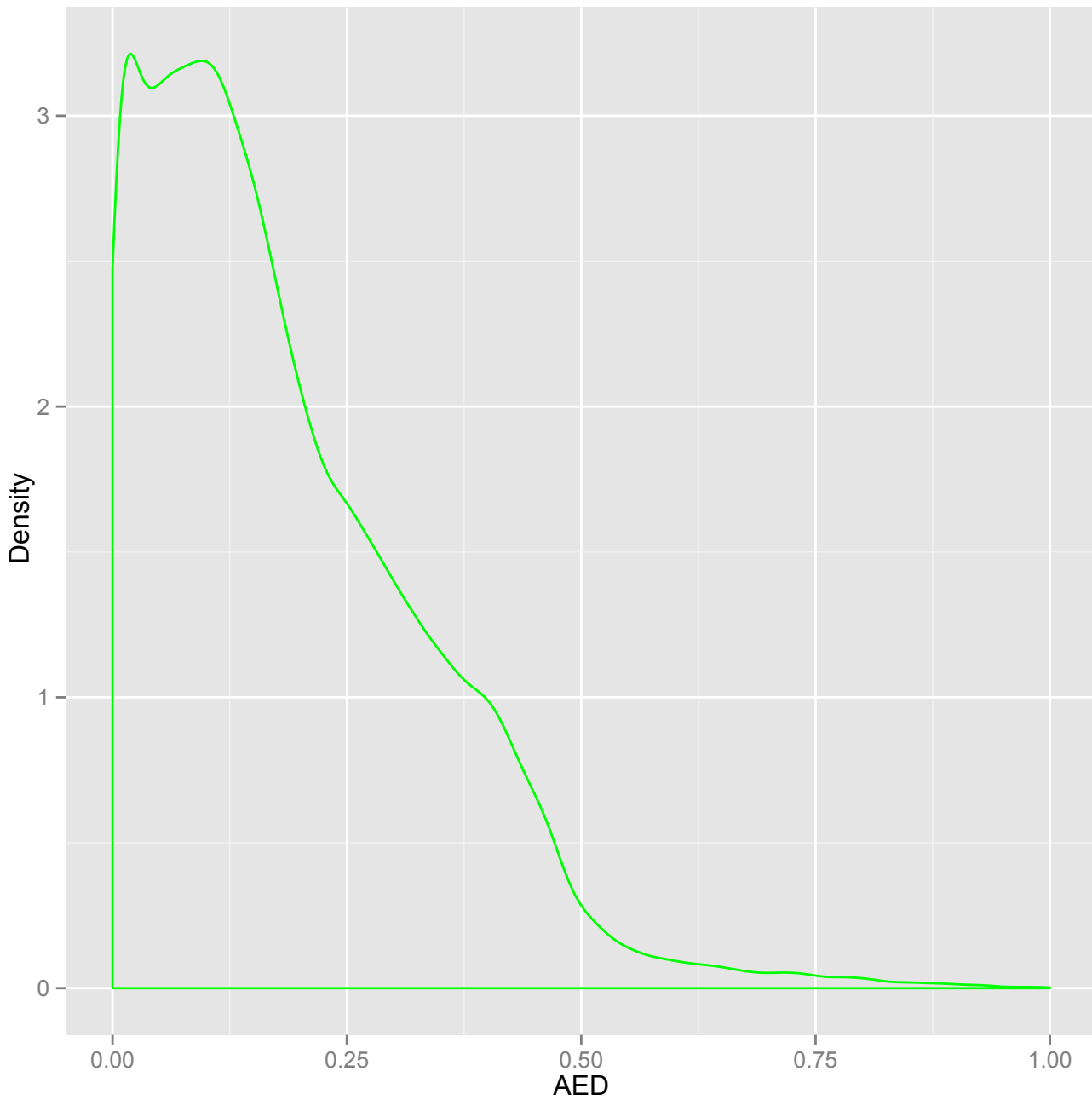

Supplement: Additional Files [file giy068_supplemental_files.zip › Fig_S3.pdf]

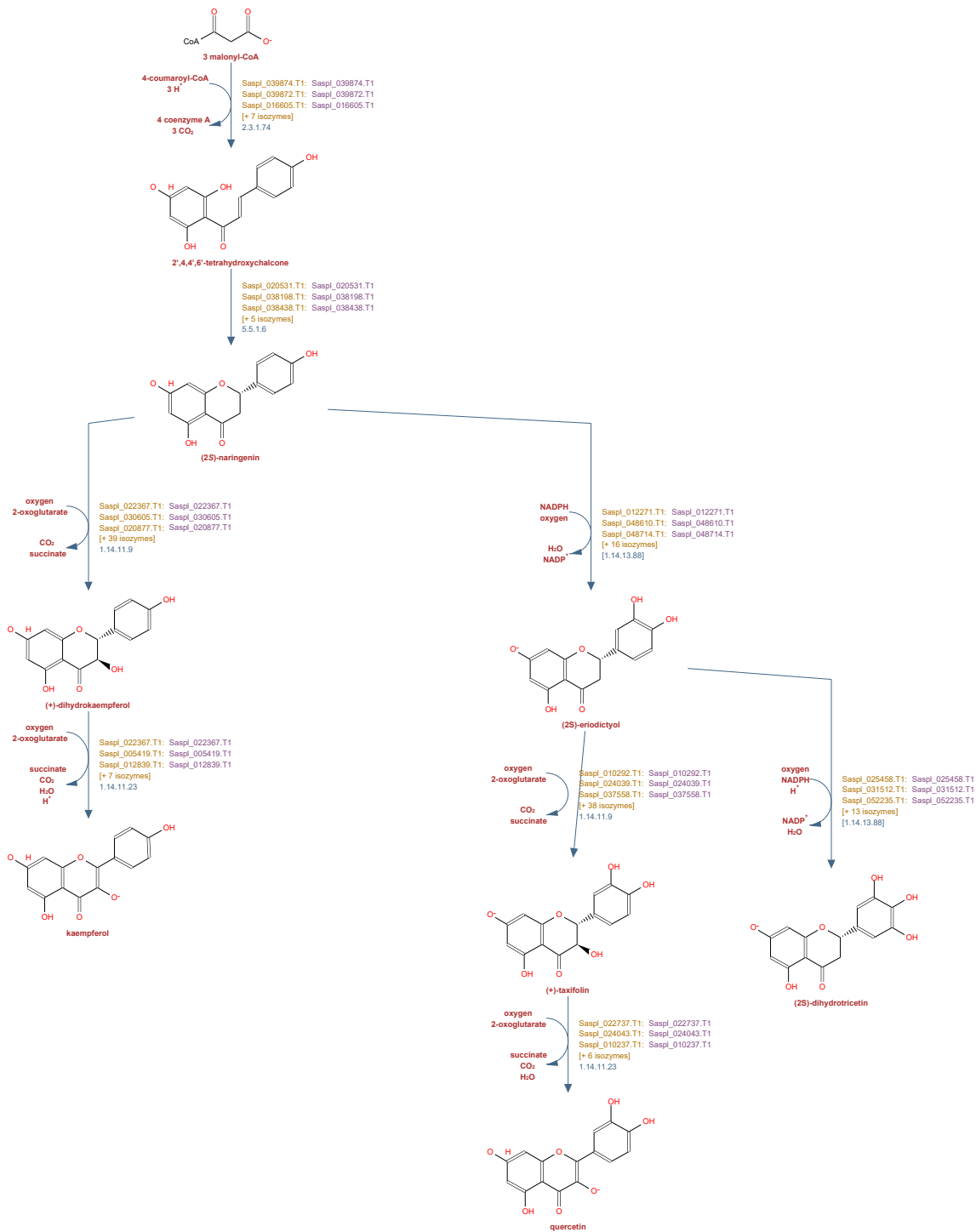

Supplement: Additional Files [file giy068_supplemental_files.zip › Fig_S5.pdf]

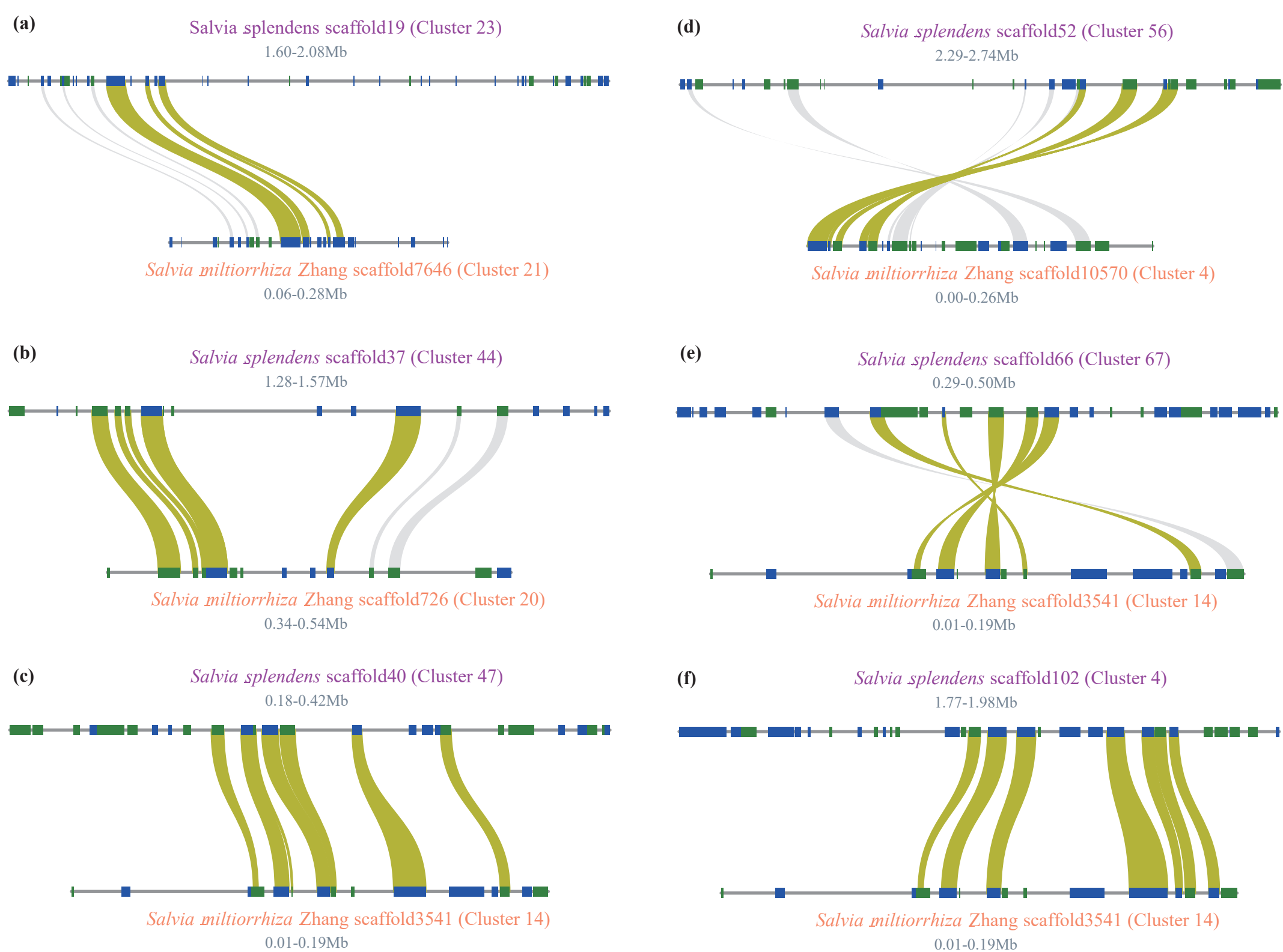

Supplement: Additional Files [file giy068_supplemental_files.zip › Fig_S7.pdf]
