# Supplementary material for: High-quality assembly of the reference genome for scarlet sage, Salvia splendens, an economically important ornamental plant
Source: Gigascience. 2018 Jun 19;7(7):giy068. doi: 10.1093/gigascience/giy068 (PMC6030905; doi:10.1093/gigascience/giy068)
Supplement: Response_to_Reviewer_Comments_Original_Submission.pdf [file giy068_response_to_reviewer_comments_original_submission.pdf]

Author's Response To Reviewer Comments

Close

We provide our comments directly underneath the points raised by you and within the three reviewers' reports as follows:

AE: Please pay particular attention to reviewer #2's comment number 3: "Since the genomes of Salvia miltiorrhiza (Zhang et al. and Xu et al.) and Mentha longifolia have been published, a more detailed analysis about differences between Salvia splendens and the other two plants should be conducted, so as to highlight the importance of Salvia splendens. "
R: We provided synteny analyses among detected metabolic gene cluster between the Salvia genomes. One section of comparative genomics was added (also see Figure S7 for synteny blocks). However, even mentha genome has been published, its gene annotation data are not publicly available. We wrote two emails to the corresponding authors for two times, we did not get any response. So mentha genome was not included in out comparative genomic studies.

AE: Your manuscript is under consideration as a Data Note, and although we do not require in-depth exploration of biological questions for this article type, I fully agree with the referee that it is crucially important that you provide some detailed context regarding the other published Salvia and Mentha genomes - what are similarities and differences, and what are unique features of Salvia splendens.
R: please see answer provided above.

AE: Please also clarify a number of technical issues mentioned by reviewer 3, e.g. regarding your scaffolding approach, as well as the use of Pilon and BUSCO.
R: Please see answers to Reviewer 3.

AE: As an editorial point, I notice that you indicate 4 "equally contributing" first authors. Please note that we allow a maximum of 3 co-first authors (and only if their contributions are really absolutely equal). Please revise the author role indications accordingly.
R: Revised. Now we have 3 co-first authors.

Reviewer #1: The authors of "High quality assembly of the reference genome for scarlet sage, Salvia splendens, an economically important ornamental plant" describe their efforts in generating a reference sequence for the plant Salvia splendens that is spread out in multiple gardens. Overall the authors relied mainly on Pacbio to obtain a high quality reference genome sequence using state of the art methods. Furthermore, they annotated the genome using RNA-Seq reads and state of the art methods such as maker, Augustus etc. Thus, I don't have any comments or concerns.
R: Thank you.

Reviewer #2: This manuscript described the construction of genome sequence and annotation for Salvia splendens Ker-Gawler. A hybrid approach using PacBio Single-Molecule Real-Time (SMRT) and Illumina HiSeq sequencing platforms was employed. Finally, a genome of 808Mb and 54,008 protein-coding genes were reported. The genome should be pretty completed because 1) the genome size is already bigger than the k-mer estimated genome size; 2) supported by BUSCO results and 3) satisfactory N50 and contig /

scaffold number. However, this is not the first species of the same genus and more functional information should be included to improve the novelty and usefulness of this piece of work. Otherwise, this will be only another genome sequence deposited in the database.
R: Thank you. Regarding more functional information provision from genomic data, please see our comments immediately below.

Reviewer #2: Comments and suggestions:
2.1. As mentioned in the introduction, many species of this genus are extensively used for culinary purposes, essential oil production and Chinese herbal remedies. Therefore, it is expected that the active ingredients of the plant responsible for its biological and therapeutic functions should be quite well known. If the metabolic pathways responsible for the production of these ingredients could be dissected, the information reported could be more useful for researchers working on this plant species.
R: One section (lines 284-332) involving description and analysis of metabolic pathways, gene clusters and comparative genomics was added. Two pathways of flavonoid and menthol biosynthesis were constructed by homolog mapping with the help of the Plant Metabolic Network (PMN v12.5, https://www.plantcyc.org/). Results were summarized in Figure S5 and S6, Supplementary_File_1.

2.2. Regarding the transcriptome analysis, results had been generated using tissues obtained from roots, shoots, leaves, calyxes and corollas. For gene discovery, mixing all the datasets to generate the transcript set is reasonable. However, to highlight the therapeutic value of particular part(s) of the plant, differential expression analysis and gene clustering would be expected.
R: Yes, this true. Our immediate intention was to identify the overall metabolic gene clusters for the two Salvia genomes, and related gene co-expression profiles were further examined among the co-localized genes. These gene clusters were summarized in Table S13, and genomic composition of gene clusters and gene expression were detailed in Supplementary File 2 and 3 (lines 284-321). A follow-up study could now target more specifically the genes of interest that promise to be correlated with variation in the therapeutic value of certain compounds and in the different plant parts and confidently identify those with the highest value.

2.3. Since the genomes of Salvia miltiorrhiza (Zhang et al. and Xu et al.) and Mentha longifolia have been published, a more detailed analysis about differences between Salvia splendens and the other two plants should be conducted, so as to highlight the importance of Salvia splendens. Moreover, the functional significance of such differences should be extensively explored and discussed. Finally, certain experiments should be done if necessary.
R: We provided synteny analyses among the detected metabolic gene cluster between the Salvia genomes. One section (lines 284-332) of comparative genomics was added to our manuscript (also see Figure S7). However, even though the mentha genome has been published, curiously, its gene annotation data is not publicly available! We wrote two emails to the corresponding authors, but we did not get any response. Thus, at this time, unfortunately, the mentha genome could not be included in our comparative genomic studies.

Reviewer #3: Dong et al. provide a near complete reference genome for the ornamental crop Salvia splendens using a PacBio sequencing approach. The assembly is high quality and will

be useful for the plant comparative genomics community. The approaches are technically sound and adequate details on the assembly and annotation of this genome are provided. I have a few minor concerns I feel should be addressed before this manuscript is published.
R: Thanks.

Reviewer #3: Comments and suggestions:
3.1 The assembly metrics of the Salvia genome are exceptionally good and the near completeness of this assembly will make it useful for the comparative genomics community. The scaffolding is potentially problematic given the short read lengths of the Illumina data and the lack of an additional set of PacBio data that was not utilized in the initial assembly. The authors used 4-5 different scaffolding algorithms on the same datasets, potentially introducing errors. Most of these scaffolding and gap filling programs were designed to utilize mate pair data to bridge repeats and not the short insert libraries produced by the authors. The Illumina data could falsely bridge gaps creating chimeric, misassembled scaffolds.
R: Indeed, we used two sets of PacBio reads from two individual plants, and just one set of Illumina reads. Genome assembly was processed in two main steps in this study as follows: We firstly generated the primary assemblies with different algorithms based on one set of PacBio reads. Then, the other set of PacBio reads was utilized in a further scaffolding step starting from the best assembly from the primary step. We provided a detailed description for genome assembly in this revision now to avoid ambiguity in the method description. We were trying to explore extra information from the Illumina short reads in the second scaffolding step, while taking care of the potential false bridge. In fact, Illumina did provide us only few values.

3.3 Line 162. The aligner used to map the Illumina reads to the Salvia genome for Pilon based polishing should be provided. Parameters for Pilon and the number of corrected indels/SNPs should also be listed.
R: Yes, we did it. Pl. see lines 164-170.

3.4 Line 216 and Line 225: It is unclear why two different BUSCO datasets were used to verify the completeness of the genome assembly/annotation.
R: We assured that only one BUSCO dataset (1,440 single copy orthologs of the Viridiplantae database) was used in this study. We wrongly input the description for BUSCO dataset. Now we corrected it throughout the text.

3.5 It would be interesting to include more downstream comparative genomics analyses for this species, but I suspect this is beyond the scope of this manuscript.
R: We did further provide functional analyses according to the second reviewer. However, no real comparative genomic analyses were provided as published genomes of Salvia miltiorrhiza (Zhang et al. and Xu et al.) and Mentha longifolia are really low quality or no protein annotation has yet been released which prevented further comparative study.

Close
