# Supplementary material for: High-quality assembly of the reference genome for scarlet sage, Salvia splendens, an economically important ornamental plant
Source: Gigascience. 2018 Jun 19;7(7):giy068. doi: 10.1093/gigascience/giy068 (PMC6030905; doi:10.1093/gigascience/giy068)
Supplement: Reviewer_2_Report_(Original_Submission) -- Stephen Tsui — 2/22/2018 Reviewed [file giy068_reviewer_2_report_(original_submission).pdf]

Reviewer Report

Title: High quality assembly of the reference genome for scarlet sage, Salvia splendens, an economically important ornamental plant

Version: Original Submission **Date:** 2/22/2018

Reviewer name: Stephen Tsui

Reviewer Comments to Author:

This manuscript described the construction of genome sequence and annotation for Salvia splendens Ker-Gawler. A hybrid approach using PacBio Single-Molecule Real-Time (SMRT) and Illumina HiSeq sequencing platforms was employed. Finally, a genome of 808Mb and 54,008 protein-coding genes were reported. The genome should be pretty completed because 1) the genome size is already bigger than the k-mer estimated genome size; 2) supported by BUSCO results and 3) satisfactory N50 and contig / scaffold number. However, this is not the first species of the same genus and more functional information should be included to improve the novelty and usefulness of this piece of work. Otherwise, this will be only another genome sequence deposited in the database.Comments and suggestions:1. As mentioned in the introduction, many species of this genus are extensively used for culinary purposes, essential oil production and Chinese herbal remedies. Therefore, it is expected that the active ingredients of the plant responsible for its biological and therapeutic functions should be quite well known. If the metabolic pathways responsible for the production of these ingredients could be dissected, the information reported could be more useful for researchers working on this plant species.2. Regarding the transcriptome analysis, results had been generated using tissues obtained from roots, shoots, leaves, calyxes and corollas. For gene discovery, mixing all the datasets to generate the transcript set is reasonable. However, to highlight the therapeutic value of particular part(s) of the plant, differential expression analysis and gene clustering would be expected. 3. Since the genomes of Salvia miltiorrhiza (Zhang et al. and Xu et al.) and Mentha longifolia have been published, a more detailed analysis about differences between Salvia splendens and the other two plants should be conducted, so as to highlight the importance of Salvia splendens. Moreover, the functional significance of such differences should be extensively explored and discussed. Finally, certain experiments should be done if necessary.

Level of Interest

Please indicate how interesting you found the manuscript: An article whose findings are important to those with closely related research interests

Quality of Written English

Please indicate the quality of language in the manuscript: Needs some language corrections before being published

Declaration of Competing Interests

Please complete a declaration of competing interests, considering the following questions:

- Have you in the past five years received reimbursements, fees, funding, or salary from an organisation that may in any way gain or lose financially from the publication of this manuscript, either now or in the future?

- Do you hold any stocks or shares in an organisation that may in any way gain or lose financially from the publication of this manuscript, either now or in the future?
- Do you hold or are you currently applying for any patents relating to the content of the manuscript?
- Have you received reimbursements, fees, funding, or salary from an organization that holds or has applied for patents relating to the content of the manuscript?
- Do you have any other financial competing interests?
- Do you have any non-financial competing interests in relation to this paper?

If you can answer no to all of the above, write 'I declare that I have no competing interests' below. If your reply is yes to any, please give details below.

I declare that I have no competing interests.

I agree to the open peer review policy of the journal. I understand that my name will be included on my report to the authors and, if the manuscript is accepted for publication, my named report including any attachments I upload will be posted on the website along with the authors' responses. I agree for my report to be made available under an Open Access Creative Commons CC-BY license (http://creativecommons.org/licenses/by/4.0/). I understand that any comments which I do not wish to be included in my named report can be included as confidential comments to the editors, which will not be published.

I agree to the open peer review policy of the journal

To further support our reviewers, we have joined with Publons, where you can gain additional credit to further highlight your hard work (see: https://publons.com/journal/530/gigascience). On publication of this paper, your review will be automatically added to Publons, you can then choose whether or not to claim your Publons credit. I understand this statement. Yes
