# Supplementary material for: High-quality assembly of the reference genome for scarlet sage, Salvia splendens, an economically important ornamental plant
Source: Gigascience. 2018 Jun 19;7(7):giy068. doi: 10.1093/gigascience/giy068 (PMC6030905; doi:10.1093/gigascience/giy068)
Supplement: Reviewer_3_Report_(Original_Submission) -- Robert van Buren — 2/26/2018 Reviewed [file giy068_reviewer_3_report_(original_submission).pdf]

Reviewer Report

Title: **High quality assembly of the reference genome for scarlet sage, Salvia splendens, an economically important ornamental plant**

Version: **Original Submission** **Date:** 2/26/2018

Reviewer name: **Robert van Buren**

Reviewer Comments to Author:

Dong et al. provide a near complete reference genome for the ornamental crop Salvia splendens using a PacBio sequencing approach. The assembly is high quality and will be useful for the plant comparative genomics community. The approaches are technically sound and adequate details on the assembly and annotation of this genome are provided. I have a few minor concerns I feel should be addressed before this manuscript is published. The assembly metrics of the Salvia genome are exceptionally good and the near completeness of this assembly will make it useful for the comparative genomics community. The scaffolding is potentially problematic given the short read lengths of the Illumina data and the lack of an additional set of PacBio data that was not utilized in the initial assembly. The authors used 4-5 different scaffolding algorithms on the same datasets, potentially introducing errors. Most of these scaffolding and gap filling programs were designed to utilize mate pair data to bridge repeats and not the short insert libraries produced by the authors. The Illumina data could falsly bridge gaps creating chimeric, misassembled scaffolds. Line 162. The aligner used to map the Illumina reads to the Salvia genome for Pilon based polishing should be provided. Parameters for Pilon and the number of corrected indels/SNPs should also be listed.Line 216 and Line 225: It is unclear why two different BUSCO datasets were used to verify the completeness of the genome assembly/annotation. It would be interesting to include more downstream comparative genomics analyses for this species, but I suspect this is beyond the scope of this manuscript. The versions of each bioinformatics program should be provided.

Level of Interest

Please indicate how interesting you found the manuscript: An article whose findings are important to those with closely related research interests

Quality of Written English

Please indicate the quality of language in the manuscript: Acceptable

Declaration of Competing Interests

Please complete a declaration of competing interests, considering the following questions:

- Have you in the past five years received reimbursements, fees, funding, or salary from an organisation that may in any way gain or lose financially from the publication of this manuscript, either now or in the future?
- Do you hold any stocks or shares in an organisation that may in any way gain or lose financially from the publication of this manuscript, either now or in the future?

- Do you hold or are you currently applying for any patents relating to the content of the manuscript?
- Have you received reimbursements, fees, funding, or salary from an organization that holds or has applied for patents relating to the content of the manuscript?
- Do you have any other financial competing interests?
- Do you have any non-financial competing interests in relation to this paper?

If you can answer no to all of the above, write 'I declare that I have no competing interests' below. If your reply is yes to any, please give details below.

I declare that I have no competing interests

I agree to the open peer review policy of the journal. I understand that my name will be included on my report to the authors and, if the manuscript is accepted for publication, my named report including any attachments I upload will be posted on the website along with the authors' responses. I agree for my report to be made available under an Open Access Creative Commons CC-BY license (http://creativecommons.org/licenses/by/4.0/). I understand that any comments which I do not wish to be included in my named report can be included as confidential comments to the editors, which will not be published.

I agree to the open peer review policy of the journal

To further support our reviewers, we have joined with Publons, where you can gain additional credit to further highlight your hard work (see: https://publons.com/journal/530/gigascience). On publication of this paper, your review will be automatically added to Publons, you can then choose whether or not to claim your Publons credit. I understand this statement. Yes
